# π-Clamp-Mediated Homo- and Heterodimerization of Single-Domain Antibodies via Site-Specific Homobifunctional Conjugation

**DOI:** 10.1021/jacs.2c04747

**Published:** 2022-07-14

**Authors:** Ross J. Taylor, Mauricio Aguilar Rangel, Michael B. Geeson, Pietro Sormanni, Michele Vendruscolo, Gonçalo J. L. Bernardes

**Affiliations:** † Centre for Misfolding Diseases, Yusuf Hamied Department of Chemistry, University of Cambridge, Lensfield Road, Cambridge CB2 1EW, U.K.; ‡ Instituto de Medicina Molecular João Lobo Antunes, Faculdade de Medicina, Universidade de Lisboa, Avenida Professor Egas Moniz, 1649-028 Lisboa, Portugal

## Abstract

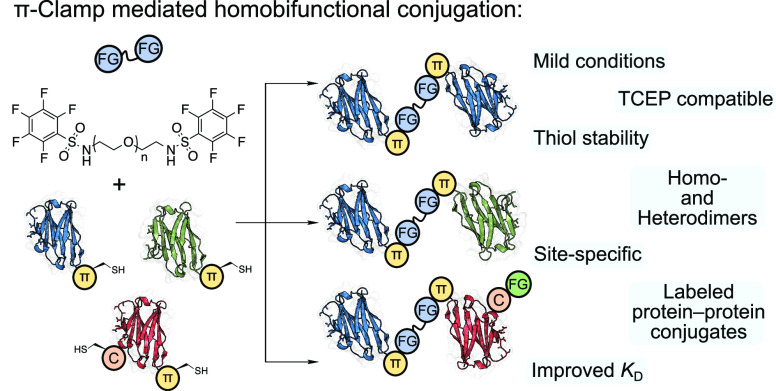

Post-translational protein–protein conjugation produces bioconjugates that are unavailable via genetic fusion approaches. A method for preparing protein–protein conjugates using π-clamp-mediated cysteine arylation with pentafluorophenyl sulfonamide functional groups is described. Two computationally designed antibodies targeting the SARS-CoV-2 receptor binding domain were produced (*K*_D_ = 146, 581 nM) with a π-clamp sequence near the C-terminus and dimerized using this method to provide a 10–60-fold increase in binding (*K*_D_ = 8–15 nM). When two solvent-exposed cysteine residues were present on the second protein domain, the π-clamp cysteine residue was selectively modified over an Asp-Cys-Glu cysteine residue, allowing for subsequent small-molecule conjugation. With this strategy, we build molecule–protein–protein conjugates with complete chemical control over the sites of modification.

The generation of protein–protein conjugates plays an important role in advancing the fields of biotechnology and biopharmaceutical research. Their applications include uses as therapeutic bispecific antibodies, bioimaging reagents, and bifunctional enzymes.^1−4^ These conjugates are typically accessed by recombinantly expressing proteins fused at the genetic level.^5,6^ Limitations of fusion proteins include incorrect folding of the constituent domains, poor stability, incompatibility of some conjugation partners, and the necessity for N-to-C terminal fusion.^5,6^ These limitations have spurred a search for alternative approaches.^7^

Post-translational protein–protein conjugation strategies are alternatives that permit greater diversity in the resulting conjugates, such as N-to-N, C-to-C, or even internally linked conjugates.^8^ Strategies to achieve this include chemically installed click handles,^9−12^ enzymatic conjugation, tag-based and noncanonical amino acid-based methods,^8,13−19^ heterobifunctional linking,^20−23^ and homobifunctional linking.^24−30^ The latter of these has an inherently simple workflow (Figure 1); the linkers are easily synthesized, and diverse linker motifs can be incorporated into protein–protein conjugates.

**Figure 1:**
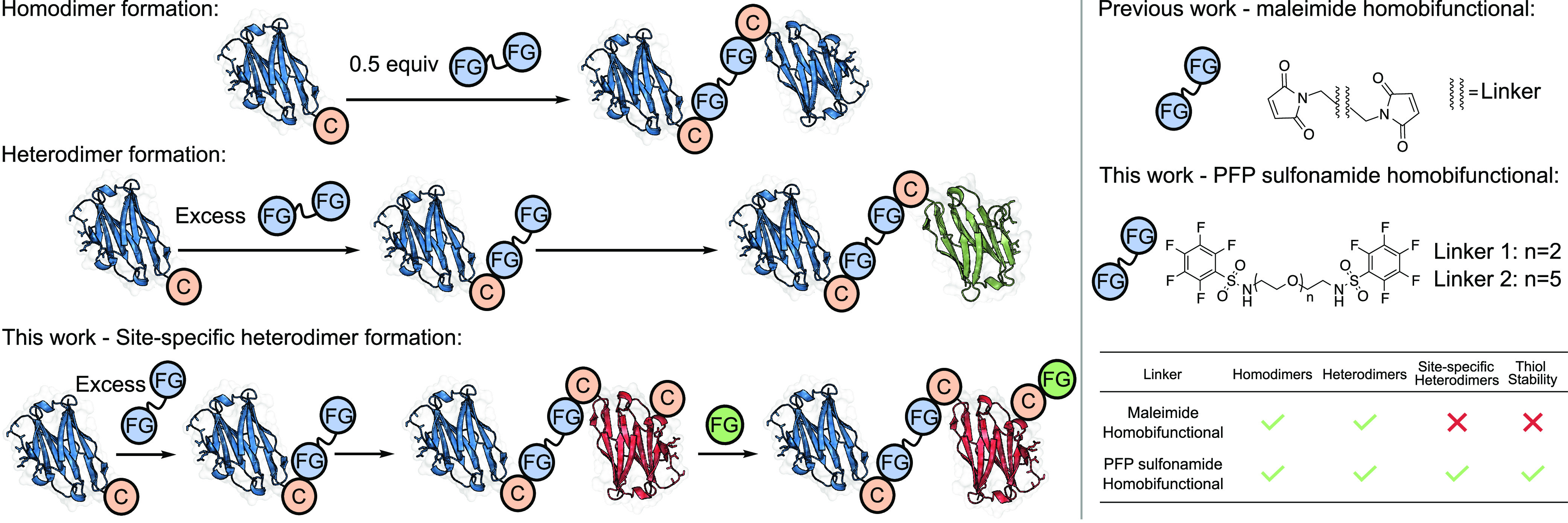
Left: Workflows for producing homodimers, heterodimers, and site-specifically labeled heterodimers. Right: Comparison of maleimide-based and pentafluorophenyl sulfonamide-based homobifunctional reagents. FG: functional group. C: cysteine residue.

Cysteine-reactive bismaleimide molecules linked by polyethylene glycol (PEG) units are the prototypical reagents for homobifunctional linking (Figure 1).^24,27,31,32^ Although they exhibit fast kinetics, the resulting linkages can undergo retro-Michael addition and subsequent thiol exchange with endogenous thiols found in serum.^33,34^ While attempts to overcome these challenges have been made,^28−30,35^ these examples have other drawbacks such as required hydrolysis at 37 °C, limited potential for linker diversification, or exclusive targeting of disulfide bonds.

We describe a homobifunctional linking strategy that bypasses these caveats, based on cysteine arylation with perfluoroaromatic reagents, resulting in stable S–C(sp^2^) bonds via S_N_Ar.^36−39^ Pentafluorophenyl (PFP) sulfonamide derivatives are reactive toward solvent-exposed cysteine residues under mild aqueous conditions, attributable to activation of the fluoride substituent para to the electron-withdrawing sulfonamide group.^36^ The π-clamp sequence (Phe-Cys-Pro-Phe), previously described by Pentelute et al.,^40,41^ significantly enhances the reactivity of the included cysteine with perfluoroaromatic compounds and has been used to generate antibody–drug conjugates (ADCs) and in PROTAC development.^40,42,43^ Here we show that the π-clamp motif is essential for producing protein–protein dimers via cysteine arylation with PFP-sulfonamide linkers and describe the first molecule–protein–protein conjugates site-specifically generated using π-clamp chemistry (Figure 1).

This linking strategy generated homo- and heterodimers of computationally designed antibodies (desAbs) targeting two distinct epitopes on the receptor binding domain (RBD) of the SARS-CoV-2 spike protein, named **C1** and **C2** (Figure 2). These antibodies possess modest *K*_D_ values (146 and 581 nM, respectively), making them an ideal system for testing affinity maturation via dimerization.^44^ The small size of single-domain antibodies (sdAbs) used as the desAb scaffold makes them susceptible to rapid clearance in vivo, so an increase in size and binding affinity could improve the half-life.^45^ Additionally, in the context of sdAbs, the paratope is sometimes proximal to the N-terminus, which means that traditional N-to-C ligation can impede binding to the target epitopes.^25^ Post-translational conjugation, however, enables conjugation of monomers through their C-terminal regions.

**Figure 2:**
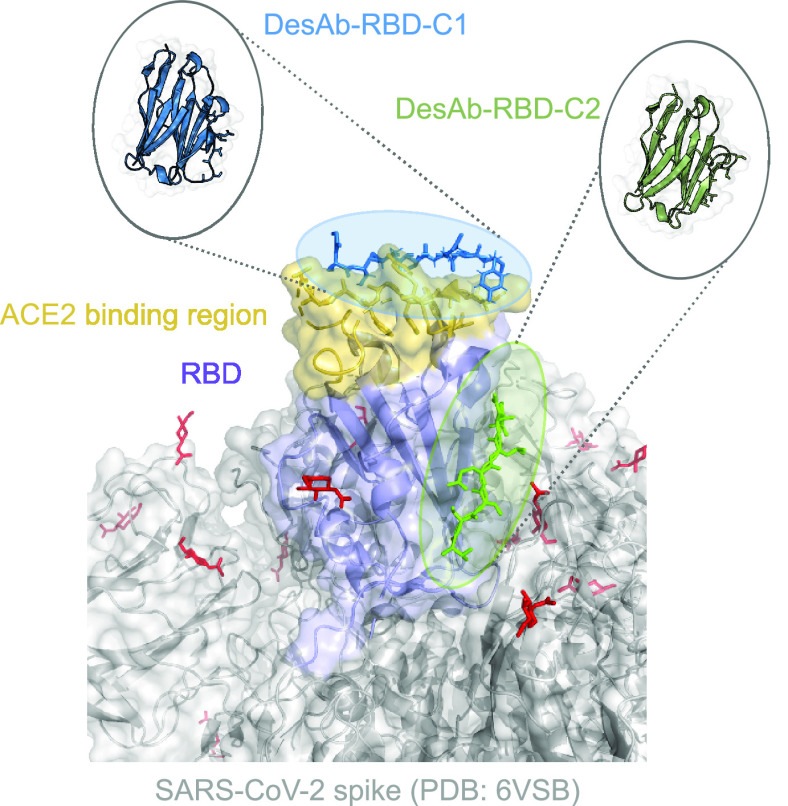
Representation of computationally designed antibodies bound to the RBD of the SARS-CoV-2 spike protein.^46^

We initially focused on using a homobifunctional reagent featuring cysteine-reactive PFP-sulfonamide groups on either end of a flexible and water-soluble PEG-2 linker, **L1** (Figure 3). A solvent-exposed cysteine residue was engineered into the **C1** antibody in an Asp-Cys-Glu motif immediately before a C-terminal tobacco etch virus (TEV) cleavage site and His tag (**C1-DCE**). It quickly became evident that the cysteine residue of **C1-DCE** was not sufficiently reactive to observe dimerization after extended incubation with **L1** (0.5 equiv) for 24 h under forcing conditions (Tris-HCl, 20 mM, pH 8.5, 37 °C, 24 h) (Figure 3a and Table S3). In addition, the reactivity of the cysteine residue in **C1-DCE** was not sufficient for conversion to a fully modified monomer after incubation with an excess of **L1** for 24 h under several sets of conditions (Tables S1 and S2).

**Figure 3:**
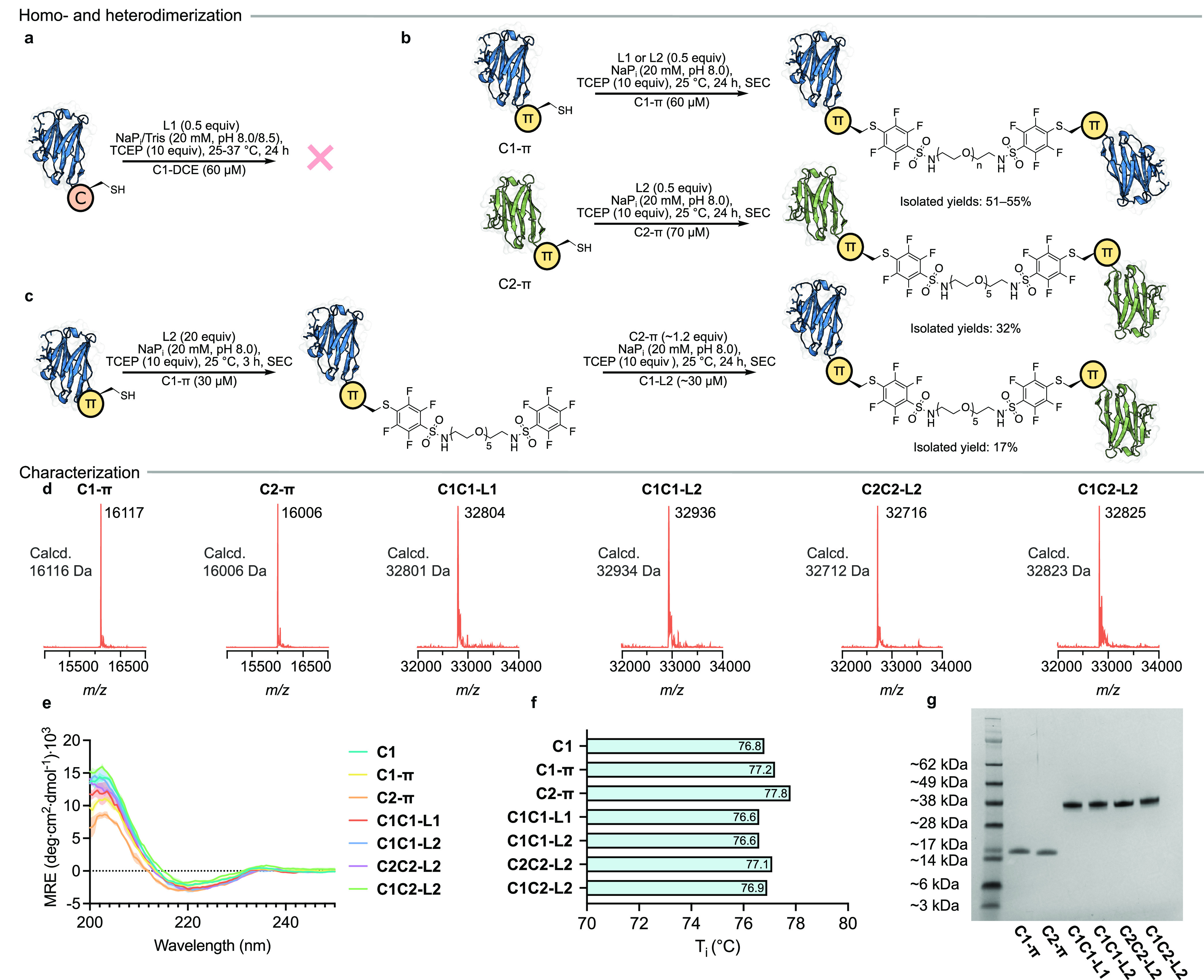
Preparation of desAb dimers using PFP-sulfonamide linkers: (a) **C1-DCE**; (b) homodimers; (c) heterodimers. Characterization of products by (d) LC–MS, (e) circular dichroism, (f) Tycho thermal denaturation, and (g) SDS-PAGE.

We rationalized that the π-clamp mediated rate enhancement observed with perfluoroaromatics would also hold with more reactive PFP-sulfonamides. Therefore, given the poor reactivity of **C1-DCE**, we engineered a π-clamp sequence into the **C1** antibody to produce **C1-π** (Figure 1). **C1-π** was significantly more reactive than **C1-DCE** and underwent full conversion to the modified monomer (**C1-L1**) within 3–5 h under mild conditions (Figure 3b and Table S4). A control alanine variant of **C1-π** (**C1-ACPA**, Ala-Cys-Pro-Ala) was prepared to confirm the π-clamp as the source of increased reactivity. Accordingly, **C1-ACPA** did not undergo dimerization under conditions otherwise identical to those used for **C1-π** (Figure S22), even in the presence of excess **L1** at both pH 8.0 and 8.5 (Table S4). These results demonstrate the π-clamp is essential for dimerization and is also important for efficiency of the initial conjugation reaction.

Upon incubation of **C1-π** in the presence of **L1** (0.5 equiv) for 24 h, homodimer **C1C1-L1** was efficiently produced (Figure 3b). Purification by size-exclusion chromatography (SEC) allowed **C1C1-L1** to be isolated in 55% yield. The purity of **C1C1-L1** was confirmed by LC–MS (Figure 3d) and SDS-PAGE analyses (Figure 3g). The related family of antibodies **C1**, **C1-π**, and **C1C1-L1** were characterized by a suite of biophysical techniques, including circular dichroism and Tycho thermal denaturation (Figure 3e,f), which assessed the impact of conjugation upon the secondary structure and thermal stability, respectively. Negligible structural perturbation was observed among **C1C1-L1**, **C1-π**, and **C1**, highlighting the mild nature of the conjugation conditions.

The homobifunctional linking strategy was initially devised to overcome the reversibility of traditional maleimide linkers. To assess this, both **C1C1-L1** and an analogous dimer produced using a bismaleimide reagent, **C1C1-M** (Figure S33), were incubated with excess glutathione at 37 °C for 3 days. Time points taken over this period showed decomposition of **C1C1-M** to its constituent monomers, while **C1C1-L1** remained intact, confirming the stability of PFP-sulfonamide-linked antibodies compared with the maleimide equivalents (Figure S36).

Linker length is an important parameter for modulating the binding properties of bioconjugates, and it is usually tuned by increasing the number of amino acids in genetic fusion or PEG units in post-translational modification, respectively. Therefore, we synthesized **L2**, a PFP-sulfonamide linker featuring a PEG-5 linker. **L2** was used to generate **C1C1-L2** in a manner identical to that for **C1C1-L1**, and **C1C1-L2** was isolated in 51% yield (Figure 3b). An equivalent π-clamp sequence was inserted near the C-terminus of **C2** to prepare **C2-π**, which was subsequently used to produce **C2C2-L2** in an isolated yield of 32% (Figure 3b). These conjugates were found to have high purity and stability as well as biophysical characteristics consistent with those of their parent monomers (Figure 3d–g).

Because of the trimeric nature of the spike protein, homodimeric bivalent antibodies could theoretically bind once to each spike protein through two RBDs (Figure 2). However, it has been shown that the spike protein is in dynamic equilibrium with “up” and “down” RBD conformations.^46^ It was unclear whether the epitopes would be fully accessible in the “down” conformation, rendering the bivalent homodimers unable to bind twice to the same spike. With this in mind, the biparatopic heterodimer (**C1C2-L2**), which is capable of binding to both exposed epitopes of a single RBD in the “up” conformation, was produced. Sequential conjugation steps were carried out to access the heterodimer **C1C2-L2** (Figure 3c). First, **C1-π** was incubated with an excess of **L2** for 3 h prior to purification via SEC and isolated in 48% yield. In a second step, purified **C1-L2** was incubated with **C2-π** (∼1.2 equiv) for 24 h to produce and isolate **C1C2-L2** in 38% yield (over two steps: 17%) with high purity and stability (Figure 3d–g).

Intrigued by the necessity for the π-clamp cysteine residue in the second conjugation reaction, we investigated the specificity for the π-clamp cysteine when more than one solvent-exposed cysteine environment was present in the second domain. To do this, we devised an experiment in which the second **C1** antibody to be sequentially conjugated had a π-clamp sequence upstream of a TEV cleavage site and an Asp-Cys-Glu motif downstream (**C1-π-DCE**). This antibody allowed the production of the heterodimer **C1-π/C1-π-DCE-L2** with a single solvent-exposed cysteine residue, which was isolated in 33% yield (over two steps: 21%) (Figure 4a,b). The solvent-exposed, reactive cysteine residue was subsequently treated with Alexa-647 maleimide to functionalize the dimer with a fluorophore (Figure 4b,c). Following this, the TEV recognition sequence was cleaved by TEV protease, concomitantly releasing the Asp-Cys-Glu sequence and thus the Alexa-647 dye. The resulting mixture was purified by immobilized metal affinity chromatography to remove the His-tagged peptides. Upon analysis of the pre- and post-TEV-cleavage mixtures by LC–MS (Figures S34 and S35) and SDS-PAGE, visualized using an Alexa-647 filter or Coomassie stain (Figure 4c), only dimeric antibody species were observed. This clearly indicated that dimerization must have proceeded solely through the π-clamp. If dimerization had occurred through the Asp-Cys-Glu motif, TEV cleavage would have resulted in deconjugation of the antibody domains and observation of monomer labeled with Alexa-647 through the π-clamp cysteine residue. Monomeric antibody species were not present after TEV cleavage, demonstrating the specificity of the second conjugation step for the π-clamp cysteine residue.

**Figure 4:**
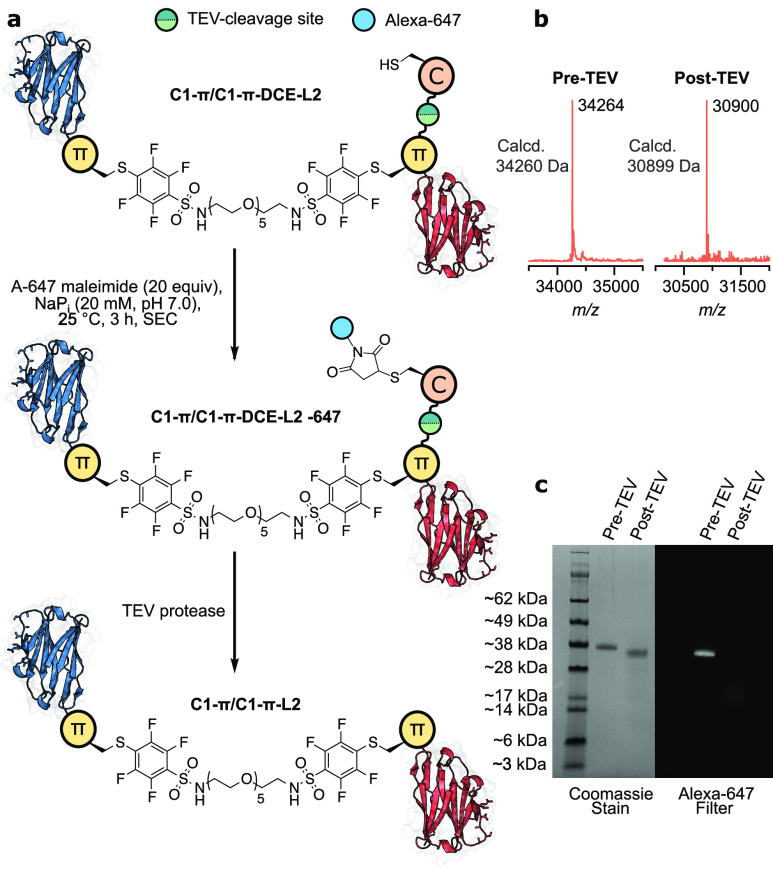
(a) Generation of a heterodimer functionalized with Alexa-647 maleimide. Characterization and validation of the modification site after TEV cleavage by (b) LC–MS and (c) SDS-PAGE.

After confirming the purity, stability, and site specificity of the conjugation strategy, we assessed the effect of dimerization on the apparent antibody *K*_D_. Initially, the *K*_D_ values for all of the homodimer constructs were measured by microscale thermophoresis (MST), a technique that relies on equilibrium thermodynamics in solution. The homodimers displayed a 10–60-fold improvement in apparent *K*_D_ with respect to their constituent monomers (Figure 5a). This is in line with expectation for a biparatopic conjugate accessing proximal epitopes on the spike, as it results in slower dissociation of the binder due to the “forced proximity effect”, i.e., increased avidity.^47^ All of the homodimers had comparable avidities, a surprising effect given the initial difference in monomer affinities. In addition, the linker length had a negligible effect on the avidity of **C1C1** constructs. Interestingly, the heterodimeric and biparatopic **C1C2-L2** did not show any further improvement compared to the bivalent homodimer constructs (Figure 5a), suggesting a similar mode of binding for all of the antibody dimers produced. As a negative control, the binding of a computationally designed anti-human serum albumin antibody (**P2**) to the spike protein was assessed by MST, and no binding was observed (Figure 5a).^44^ In addition, the PFP-sulfonamide linkers had no effect on *K*_D_ compared to the bismaleimide linker of **C1C1-M** (Figure 5a).

**Figure 5:**
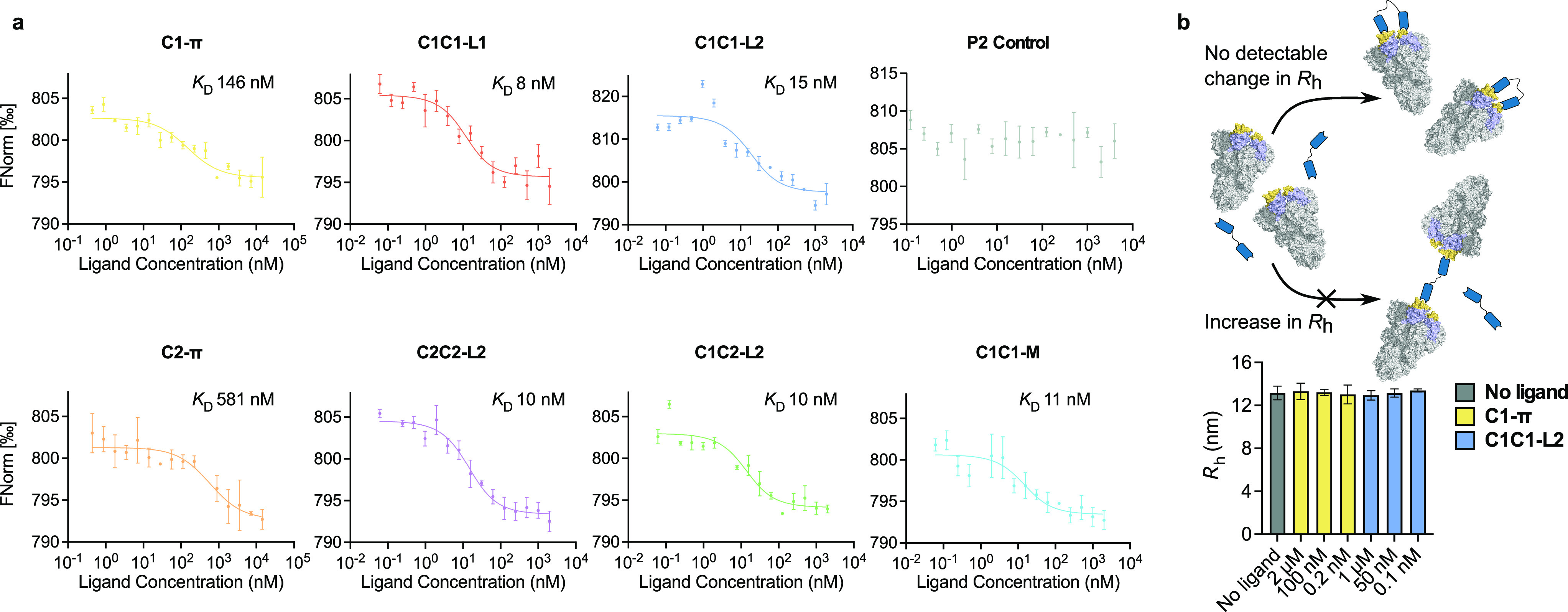
(a) Determination of monomer and dimer *K*_D_ values by microscale thermophoresis (MST data for **C1-π**, **C2-π**, and **P2** are from ref (44)). (b) Schematic showing the expected change in *R*_h_ of free spike protein arising from complexation with **C1C1-L2** and **C1-π**.

Because of the aforementioned equilibrium of “up” and “down” conformations of the RBD, the mode of binding of the homodimeric, bivalent antibodies to the spike protein was investigated. We envisioned two possible scenarios: (i) both units of the dimer bind to two RBDs on the same spike; (ii) each unit of the dimer binds to an RBD on a different spike molecule. The second scenario would result in a dramatically increased hydrodynamic radius (*R*_h_) of the complex (Figure 5b). To assess this, the spike protein was incubated with varying concentrations of **C1C1-L2**, and *R*_h_ was measured by microfluidic diffusional sizing. Minimal variation in *R*_h_ for the bound and unbound states was observed (Figure 5b),^48^ indicating that the entropically favored bidentate mode of binding to the spike protein is operational. As a control experiment, the **C1-π** monomer produced an invariant *R*_h_ value (Figure 5b).

PFP-sulfonamide-based linking reagents enabled the generation of bivalent and biparatopic antibody homo- and heterodimers of **C1** and **C2**. This mild conjugation approach maintained the structural integrity of the constituent domains while generating conjugates with enhanced stability compared with bismaleimide conjugation. The antibody dimers produced an order of magnitude improvement in binding to the spike protein relative to the parent monomers, due to avidity effects. In addition, the specificity of the PFP-sulfonamide functionality for π-clamp cysteine residues allowed labeling of other solvent-exposed cysteine residues. This novel feature of the PFP-sulfonamide linkers will enable the generation of bivalent, biparatopic, or bispecific ADCs, which have the potential to be powerful therapeutics.

## Supporting Information Available

Detailed methods, characterization data, and additional figures (PDF)
