# Supplementary material for: π-Clamp-Mediated Homo- and Heterodimerization of Single-Domain Antibodies via Site-Specific Homobifunctional Conjugation
Source: J Am Chem Soc. 2022 Jul 14;144(29):13026–31. doi: 10.1021/jacs.2c04747 (PMC9335888; doi:10.1021/jacs.2c04747)
Supplement: Supplementary file 1 — ja2c04747_si_001.pdf [file ja2c04747_si_001.pdf]

Supporting Information:

π-Clamp-Mediated Homo- and Heterodimerization of Single Domain Antibodies via Site-Specific Homobifunctional Conjugation

Ross J. Taylor,[†] Mauricio Aguilar Rangel,[†] Michael B. Geeson,[†] Pietro Sormanni,[†] Michele Vendruscolo,[*,†] and Gonçalo J. L. Bernardes[*,†,‡]

†*Centre for Misfolding Diseases, Yusuf Hamied Department of Chemistry, University of Cambridge, Lensfield Road, CB2 1EW Cambridge, UK*

‡*Instituto de Medicina Molecular João Lobo Antunes, Faculdade de Medicina, Universidade de Lisboa, Avenida Professor Egas Moniz, 1649-028, Lisboa, Portugal*

E-mail: mv245@cam.ac.uk; gb453@cam.ac.uk

Contents

1 General experimental procedures **S-3**

2 Synthetic procedures **S-4**

3 Cloning of desAb variants **S-7**

3.1 RF cloning primers S-7

4 Designed antibody amino acid sequences **S-9**

5 Expression and purification of desAb variants **S-11**

6 General LC–MS protocols **S-12**

7 Protein conjugation experiments **S-13**
7.1 General Protein Preparation S-13
7.2 LC–MS spectra of unmodified proteins S-14
7.3 Test reactions S-20
7.4 General homodimerisation procedure S-39
7.5 Heterodimerisation of C1 and C2 . . . . S-39
7.6 Homodimerisation using bis-maleimide linker S-40
7.7 SDS–PAGE and LC–MS analysis of homo- and heterodimerisation reactions S-41
7.7.1 SDS–PAGE analysis S-41
7.7.2 LC–MS analysis after 24 h incubation S-42
7.7.3 LC–MS analysis after SEC purification S-48
7.8 Heterodimerisation of double cysteine containing variant S-53
7.9 Glutathione stability assay S-56

8 Linker extinction coefficient determination **S-57**

9 Biophysical characterisation **S-58**
9.1 Circular dichroism S-58
9.2 Tycho thermal denaturation assays S-60

10 Antibody binding assays **S-61**
10.1 Alexa-647 NHS ester labelling of the SARS-CoV-2 Spike protein for MST and R_h measurements S-61
10.2 Microscale thermophoresis (MST) binding affinity measurements S-62
10.3 Hydrodynamic radius measurements using Fluidity One W S-63

11 ^{1}H, ^{13}C and ^{19}F NMR Spectra **S-64**

References **S-69**

1 General experimental procedures

All solvents and reagents were purchased from commercial sources and used without further purification. Petroleum ether 40-60, *n*-hexane, EtOAc and dichloromethane were distilled on site. Commercially obtained LC–MS hypergrade acetonitrile and MQ water prepared on site were used as solvents for the mobile phase in LC–MS experiments. NMR: ^{1}H, ^{13}C and ^{19}F NMR spectra were recorded on Bruker 400-Avance III HD or Bruker 700-Avance II+ TXO cryoprobe NMR machines. Chemical shifts were reported in parts per million (ppm) with spectra calibrated to the residual solvent peaks (^{1}H NMR: $CDCl_3$ δ_H 7.26 ppm, ^{13}C NMR: $CDCl_3$ δ_C 77.16 ppm). MestReNova software (v. 14.2.0) was used for spectral processing. Chromatography: Purification via flash chromatography was performed over silica gel (Geduran silica gel 60, 40-63 µm; Merck). Reaction progress and elution of products during chromatography were monitored by TLC (silica gel 60 on glass, with indicator F254; Merck) and visualised under UV light, or by staining with suitable solution prepared by known procedures. All buffers for bioconjugation were prepared on site. TCEP and reduced glutathione were prepared in the relevant buffer for each reaction and the pH adjusted as required.

2 Synthetic procedures

N,N′-((ethane-1,2-diylbis(oxy))-bis(ethane-2,1-diyl))bis(2,3,4,5,6-pentafluorobenzenesulfonamide) (Linker 1)

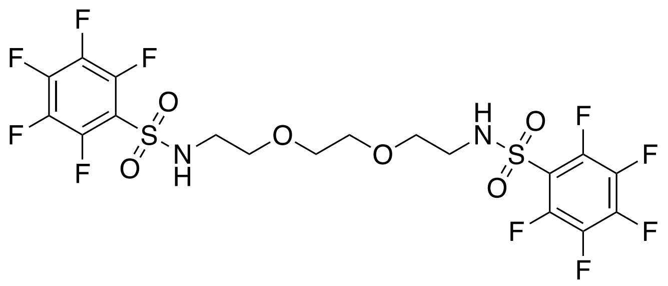

Pentafluorobenzenesulfonyl chloride (150 mg, 0.56 mmol, 1 equivalents) was dissolved in DCM (3 mL) and added dropwise with stirring at 0 °C to a solution of 2,2′-(Ethylenedioxy)bis-(ethylamine) (41.7 mg, 0.28 mmol, 0.5 equivalents) and triethylamine (170.9 mg, 1.69 mmol, 3 equivalents) in DCM (3 mL) and allowed to come to room temperature. The reaction was stirred at room temperature for 16 h before being concentrated under reduced pressure, dissolved in DCM (10 mL) and washed with saturated $NaHCO_3$ (3 x 10 mL) and brine (1 x 10 mL). The organic layer was dried of $MgSO_4$ and concentrated to afford the title compound as a white solid (90 mg, 62%) which was used without further purification.

^{1}H NMR δ_H (700 MHz,$CDCl_3$) 6.04 (2H, s, br, NH), 3.65 (4H, t, J = 5.0 Hz), 3.62 (4H, s), 3.36 (4H, t, J = 5.1 Hz); ^{13}C NMR δ_C (101 MHz, $CDCl_3$) 70.8, 69.7, 43.5; ^{19}F NMR δ_F (376 MHz, $CDCl_3$) -137.86 – -138.19 (2F, m), -147.02 – -147.21 (1F, m), -159.57– -159.84 (2F, m); HRMS (ESI+): $[M+H]^+$ Calculated: ($C_{18}H_{15}F_{10}N_6O_2S_2$) 609.0212 Found: 609.0225

*Aromatic C–F carbons are not detected because of the high multiplicity derived from C–F coupling.

3,6,9,12,15-pentaoxaheptadecane-1,17-diamine

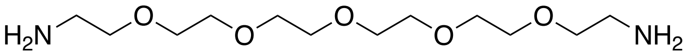

3,6,9,12,15-pentaoxaheptadecane-1,17-diazide (690 mg, 2.08 mmol) was dissolved in MeOH (80 mL) and 10% Pd/C (ca. 10 mol%) was added with vigorous stirring. The reaction flask was flushed with H_2 and subsequently left to stir for 16 h at r.t. The reaction mixture was filtered through celite and concentrated to afford the title compound as a colourless oil (551 mg, 94%).

^{1}H NMR δ_H (400 MHz, $CDCl_3$) 3.62 – 3.54 (20H, m), 3.45 (4H, t, J = 5.1 Hz), 2.80 (4H, t, J = 5.2 Hz), 2.20 (6H, s); ^{13}C NMR δ_C (101 MHz, $CDCl_3$) 73.0, 70.5, 70.2, 41.5.

N,N′-(3,6,9,12,15-pentaoxaheptadecane-1,17-diyl)bis(2,3,4,5,6-pentafluorobenzenesulfonamide) (Linker 2)

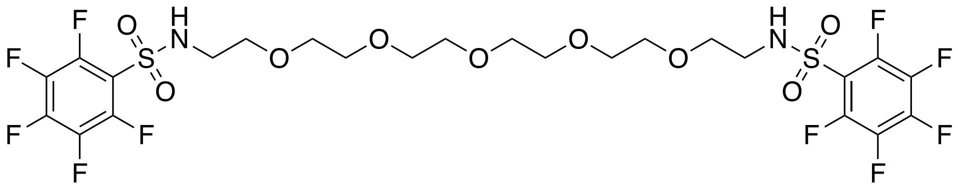

Pentafluorobenzenesulfonyl chloride (400 mg, 1.50 mmol, 1 triethylamine (313 µL, 2.25 mMol, 3 equivalents) was dissolved in DCM (5 mL) and added dropwise with stirring at 0°C to a solution of 3,6,9,12,15-pentaoxaheptadecane-1,17-diamine (210 mg, 0.75 mMol, 0.5 equivalents) and triethylamine (313 µL, 2.25 mM, 3 equivalents) in DCM (10 mL) and allowed to come to room temperature. The reaction was stirred at room temperature for 1 h before being concentrated under reduced pressure. The crude residue was purified via flash chromatography EtOAc:Petrol (80:20) to afford the title compound as a colourless oil (324.4 mg, 58%)

^{1}H NMR δ_H (700 MHz, $CDCl_3$) 7.08 (2H, s, NH), 3.64–3.54 (20 H, m, 3.33–3.28 (4H, m); ^{13}C NMR δ_C (176 MHz, $CDCl_3$) 145.2, 144.3, 143.7, 142.8, 138.5, 137.1, 117.0, 70.5, 70.4, 70.3, 70.1, 69.2, 43.4; ^{19}F NMR δ_F (376 MHz, $CDCl_3$) -136.66– -136.89 (2F, m), -147.09 – -147.29 (1F, m), -159.26–159.53 (2F, m); HRMS (ESI+): $[M+H]^+$ Calculated: ($C_{24}H_{27}F_{10}N_2O_9S_2$) 741.0998 Found: 741.0994

3 Cloning of desAb variants

The parent plasmids of pD444-C1 and C2 were readily available from previous research in the group.[S1] The insertion of tag sequences into these constructs was carried out using the previously described RF cloning method, a variation on the QuikChange II site directed mutagenesis protocol.[S2] The following primers were designed using RF-cloning.org and used to generate the recombinant DNA sequences for C1, C1-DCE, C1-π, C1-ACPA, C1-π-DCE, C2-π and P2 (C1 in this work corresponds to TEV-site containing constructs).

3.1 RF cloning primers

C1 and P2 (ENLYFQG)*

Forward::

5′–CCTGGTCACGGTGAGCAGCGACGAAAACCTGTACTTCCAGGTC–3′

Reverse:

5′–ACCTTAGTGGTGATGATGATGGTGGTGACCCTGGAAGTACAGGTTTTC–3′

C1-DCE (DCEENLYFQG)*

Forward::

5′–CCTGGTCACGGTGAGCAGCGACGACTGCGAGGAAAACCTGTATTTCCAG–3′

Reverse:

5′–ACCTTAGTGGTGATGATGATGGTGGTGACCCTGGAAATACAGGTTTTCCTCG–3′

C1-π and C2-π (FCPFENLYFQG)*

Forward::

5′–CCTGGTCACGGTGAGCAGCGACTTCTGCCCGTTTGAAAACCTGTACTTCC–3′

Reverse:

5′–ACCTTAGTGGTGATGATGATGGTGGTGACCCTGGAAGTACAGGTTTCTCAAAGG–3′

C1-ACPA (ACPAENLYFQG)*

Forward::

5′–CCTGGTCACGGTGAGCAGCGACGCTTGTCCGGCTGAAAACCTGTATTTC–3′

Reverse:

5′–ACCTTAGTGGTGATGATGATGGTGGTGACCCTGGAAATACAGGTTTTCAGCCGG–3′

C1-π-DCE (FCPFENLYFQGDCE)*

Forward::

5′ –CCTGGTCACGGTGAGCAGCGACTTCTGTCCGTTCGAAAACCTGTACTTTCAG-GGT–3′

Reverse:

5′–ACCTTAGTGGTGATGATGATGGTGGTGTTCGCAATCACCCTGAAAGTACAGGT-TTTCG–3′

*Denotes inserted amino acid sequence.

4 Designed antibody amino acid sequences

C1

MEVQLEESGGGLVQPGGSLRLSCAASGFNIKDTYIGWVRQAPGKGEEWVASIYPTS
GYTRYADSVKGRFTISADTSKNTAYLQMNSLRAEDTAVYYCAAGSGSSATEVYGSE
EEFDYWGQGTLVTVSSDENLYFQGHHHHHHH

Calculated molecular weight: 15621.99 Da

Theoretical extinction coefficient: 31525 $M^{-1}cm^{-1}$

C1-DCE

MEVQLEESGGGLVQPGGSLRLSCAASGFNIKDTYIGWVRQAPGKGEEWVASIYPTS
GYTRYADSVKGRFTISADTSKNTAYLQMNSLRAEDTAVYYCAAGSGSSATEVYGSE
EEFDYWGQGTLVTVSSDDCEENLYFQGHHHHHHH

Calculated molecular weight: 15969.34 Da

Theoretical extinction coefficient: 31525 $M^{-1}cm^{-1}$

C1-π

MEVQLEESGGGLVQPGGSLRLSCAASGFNIKDTYIGWVRQAPGKGEEWVASIYPTS
GYTRYADSVKGRFTISADTSKNTAYLQMNSLRAEDTAVYYCAAGSGSSATEVYGSE
EEFDYWGQGTLVTVSSDFCPFENLYFQGHHHHHHH

Calculated molecular weight: 16116.60 Da

Theoretical extinction coefficient: 31525 $M^{-1}cm^{-1}$

C1-ACPA

MEVQLEESGGGLVQPGGSLRLSCAASGFNIKDTYIGWVRQAPGKGEEWVASIYPTS
GYTRYADSVKGRFTISADTSKNTAYLQMNSLRAEDTAVYYCAAGSGSSATEVYGSE
EEFDYWGQGTLVTVSSDACPAENLYFQGHHHHHHH

Calculated molecular weight: 15964.41 Da

Theoretical extinction coefficient: 31525 $M^{-1}cm^{-1}$

C2-π

MEVQLEESGGGLVQPGGSLRLSCAASGFNIKDTYIGWVRQAPGKGEEWVASIYPTS
GYTRYADSVKGRFTISADTSKNTAYLQMNSLRAEDTAVYYCAAGSVVADLSVGSEE
EFDYWGQGTLVTVSSDFCPFENLYFQGHHHHHHH

Calculated molecular weight: 16005.59 Da

Theoretical extinction coefficient: 30035 $M^{-1}cm^{-1}$

C1-π-DCE

MEVQLEESGGGLVQPGGSLRLSCAASGFNIKDTYIGWVRQAPGKGEEWVASIYPTS
GYTRYADSVKGRFTISADTSKNTAYLQMNSLRAEDTAVYYCAAGSGSSATEVYGSE
EEFDYWGQGTLVTVSSDFCPFENLYFQGDCEHHHHHHH

Calculated molecular weight: 16463.95 Da

Theoretical extinction coefficient: 31650 $M^{-1}cm^{-1}$

P2

MEVQLEESGGGLVQPGGSLRLSCAASGFNIKDTYIGWVRQAPGKGEEWVASIYPTS
GYTRYADSVKGRFTISADTSKNTAYLQMNSLRAEDTAVYYCAAGSAQAGNAEEAE
EEFDYWGQGTLVTVSSDENLYFQGHHHHHHH

Calculated molecular weight: 15524.88 Da

Theoretical extinction coefficient: 30035 $M^{-1}cm^{-1}$

5 Expression and purification of desAb variants

The recombinant pD444 plasmids for C1, C1-DCE, C1-π, C1-ACPA, C1-π-DCE, C2-π, and P2 were transformed into SHuffle T7 competent *E. coli* (New England Biolabs: C3026J) and an overnight culture of these were grown in LB media (5 mL) containing ampicillin (100 µg/mL). C1, C1-DCE, C1-π, C1-ACPA, C1-π-DCE, C2-π and P2 cultures were used to inoculate 0.5 L of LB medium containing 100 µg/mL of ampicillin in 2 L flasks to reach an initial OD_{600} of 0.02. These were incubated at 37 °C, 180 rpm until an OD_{600} of 0.8 was reached. At which point, expression was induced upon addition of IPTG (500 µM). The temperature was reduced to 30 °C and the cultures left to incubate overnight at 180 rpm.

The expression media was subsequently collected after centrifugation at 8000 rpm and 4 °C for 30 minutes. The pellet was discarded, and the supernatant filtered through a 0.45 µm filter under moderate vacuum to remove cellular debris. The supernatant was loaded onto Ni-NTA resin equilibrated with PBS (pH 7.4) and passed over the resin 3x before the bound protein was eluted with an imidazole gradient The imidazole gradient consisted of 10 mM (30 mL), 40 mM (15 mL), 200 mM (5 mL) and 200 mM (10 mL) (all imidazole solutions were in PBS pH 7.4). The fractions were collected and analysed by SDS–PAGE before combining pure protein containing fractions.

Following combination of fractions, all samples were desalted using HiPrep^{TM} 26/10 Desalting column (Cytiva) eluting with PBS (pH 7.4). Protein concentrations were subsequently determined by the absorption at 280 nm using a NanoDrop 2000c UV-Vis spectrophotometer. Theoretical extinction coefficients were calculated by ProtParam-Tool ExPASy (http://expasy.org/tools/protparam.html) using the proteins' amino acid sequences.

6 General LC–MS protocols

LC–MS analysis of protein samples was carried out using a Waters SQD2 mass spectrometer using inlet methods A and B, or a Waters Xevo G2-S TOF mass spectrometer using inlet method C, in combination with an Acquity UPLC system with an Acquity UPLC BEH300 C4 column (130 Å1.7 µm, 2.1 X 50 mm). The SQD2 mass spectrometer mobile phase consisted of solvent A (99.9% water with 0.1% formic acid), solvent B (71% acetonitrile, 28.9% water and 0.1% formic acid) and the following gradients were programmed. Inlet method A: 100% A for 2 min, then 100% A to 100% B in 9 min, then 100% B for 5 min folloerd by 100% A for 4 min. Inlet method B: 100% A for 2 min, then 85% A to 100% B in 6 min, then 100% B for 2.5 min followed by 85% A for 3.5 min. The Xevo G2-S TOF mass spectrometer mobile phase consisted of solvent A (99.9% water with 0.1% formic acid), solvent B (95% MeCN and 5% water with 0.1% formic acid) and the following gradients were programmed. Inlet method C: 95% A for 0.93 min, then 95% A to 100% B in 4.28 min, then 100% B for 1.04 min, 100% B to 95% A for 1.04 min. The capillary voltage of the electrospray source for the Waters SQD2 mass spectrometer was 3.0 kV with a cone voltage of 30 V and the desolvation gas used was nitrogen, with a flow rate of 800 L h^{-1}. For the Waters Xevo G2-S TOF mass spectrometer the capillary voltage of the electrospray source was 2.0 kV with a cone voltage of 40 V and the desolvation gas used was nitrogen, with a flow rate of 850 L h^{-1}. The ion series was obtained through integration of the major peaks of the chromatogram. Following this, the total mass spectra were reconstructed using the MaxEnt1 algorithm on the MassLynx software (v. 4.1), according to manufacturer's guidelines.

7 Protein conjugation experiments

7.1 General Protein Preparation

Fresh batches of cysteine contaning desAbs in PBS (pH 7.4), were reduced using TCEP (20 equivalents) to remove gutathione and homocysteine capping which occured during expression, and concentrated using an amicon 3 kDa MWCO spin filter. Buffer exchange of reduced protein into NaPi (20 mM pH 8.0) or Tris-HCl (20 mM pH 8.5) was carried out using a 7 kDa MWCO Zeba^{TM} Spin Desalting Column equilibrated with aqueous NaPi (20 mM pH 8.0) or Tris-HCl (20 mM pH 8.5). DesAb monomer purity was assessed by LC–MS spectrometry (Figures S1-S6)

7.2 LC–MS spectra of unmodified proteins

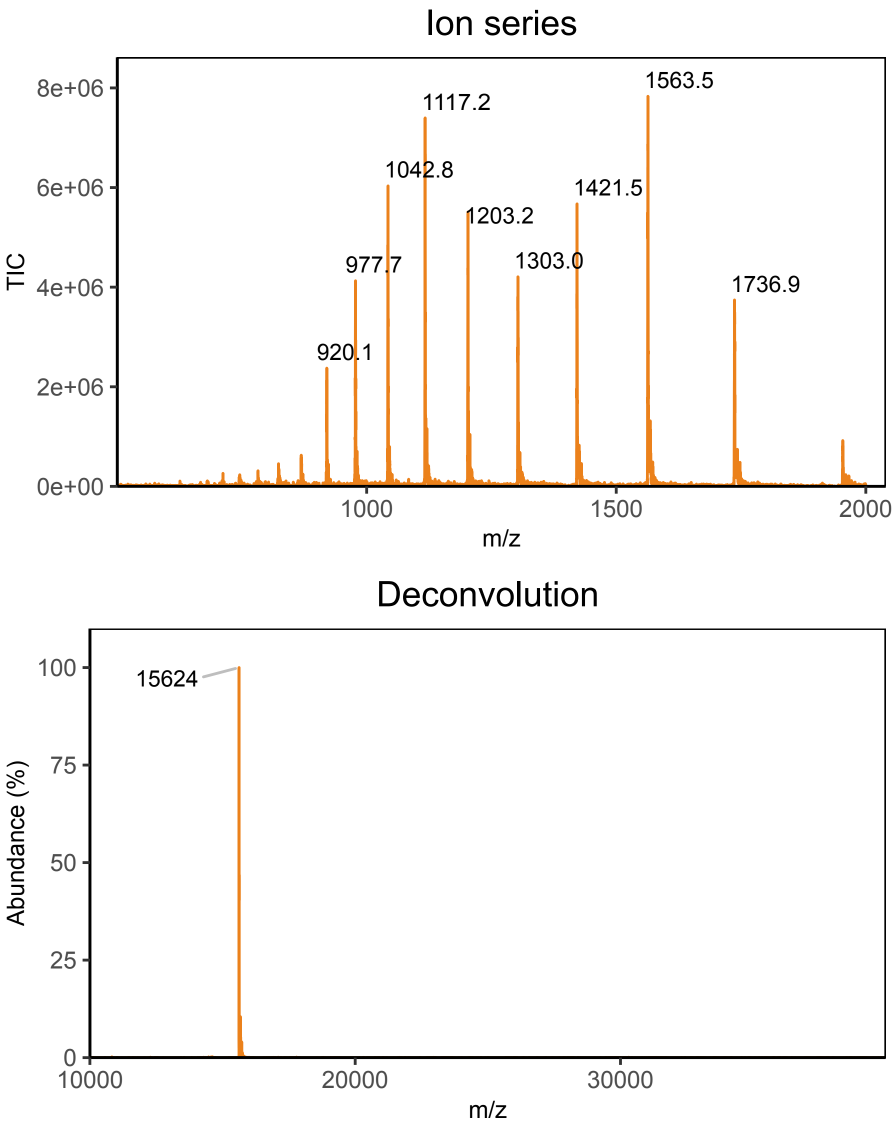

Figure S1: LC–MS spectra of C1; ion series and deconvoluted spectra (Calculated mass: 15622 kDa).

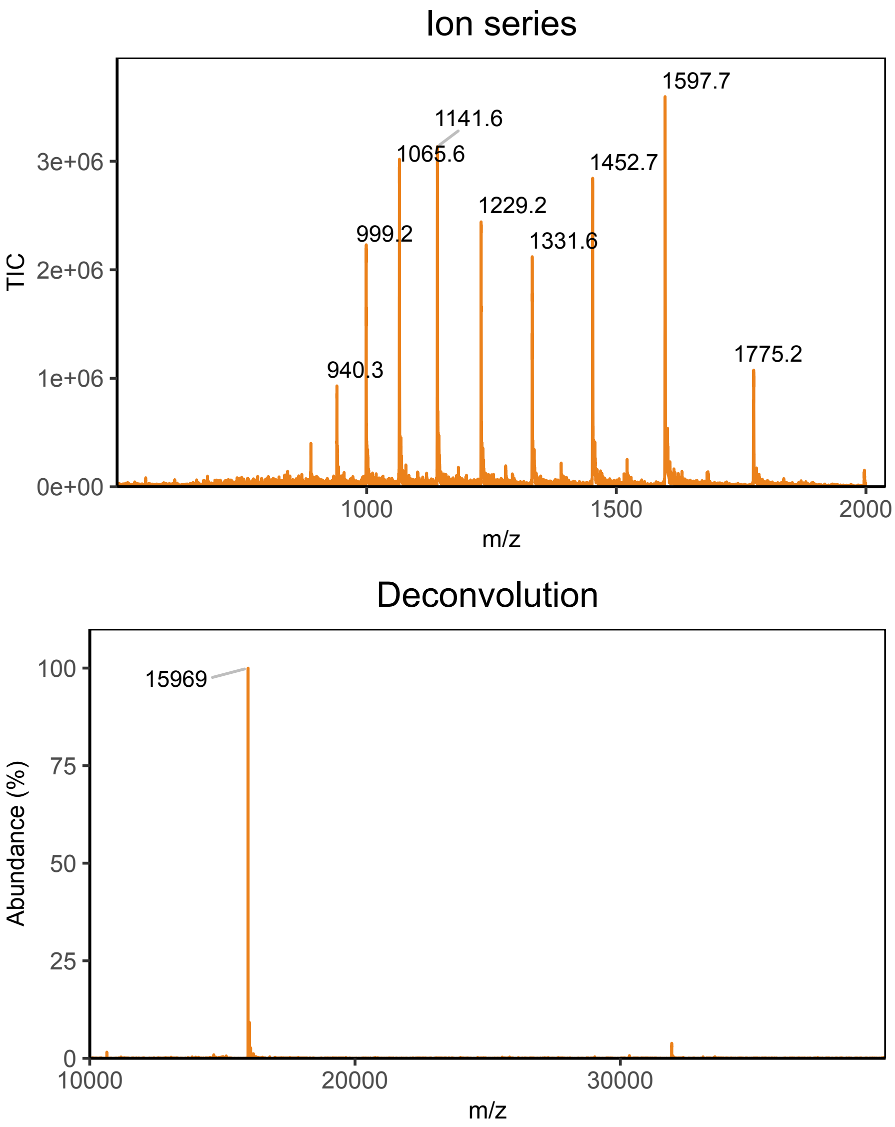

Figure S2: LC–MS spectra of C1-DCE; ion series and deconvoluted spectra (Calculated mass: 15969 kDa).

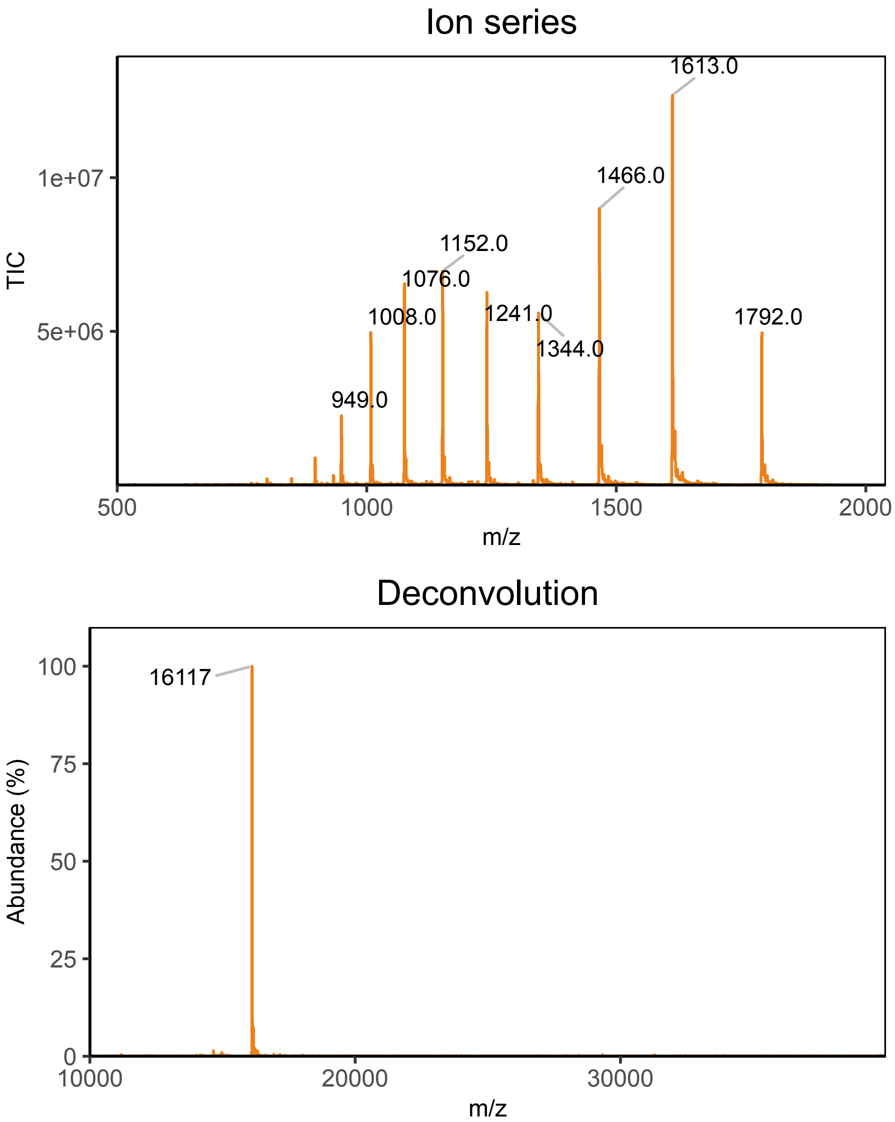

Figure S3: LC–MS spectra of C1-π; ion series and deconvoluted spectra (Calculated mass: 16117 kDa).

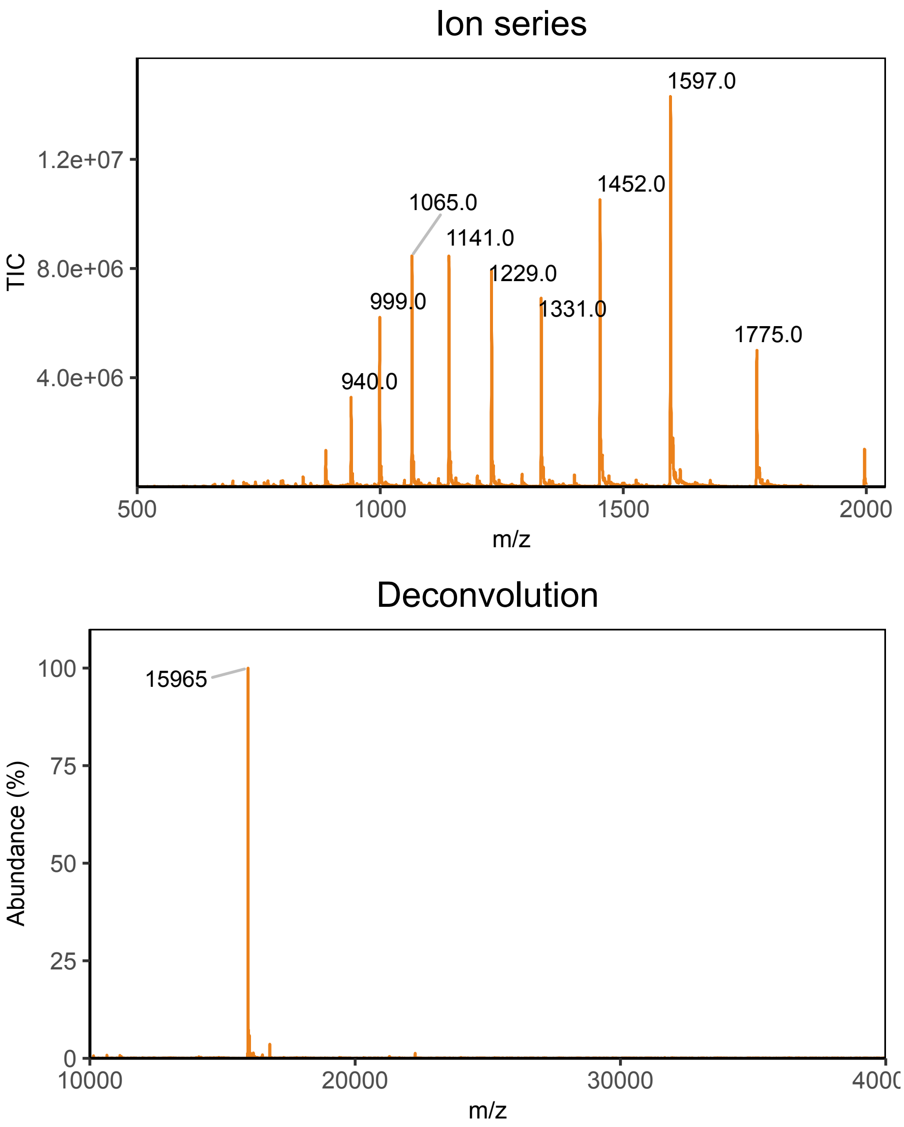

Figure S4: LC–MS spectra of C1-ACPA; ion series and deconvoluted spectra (Calculated mass: 15964 kDa).

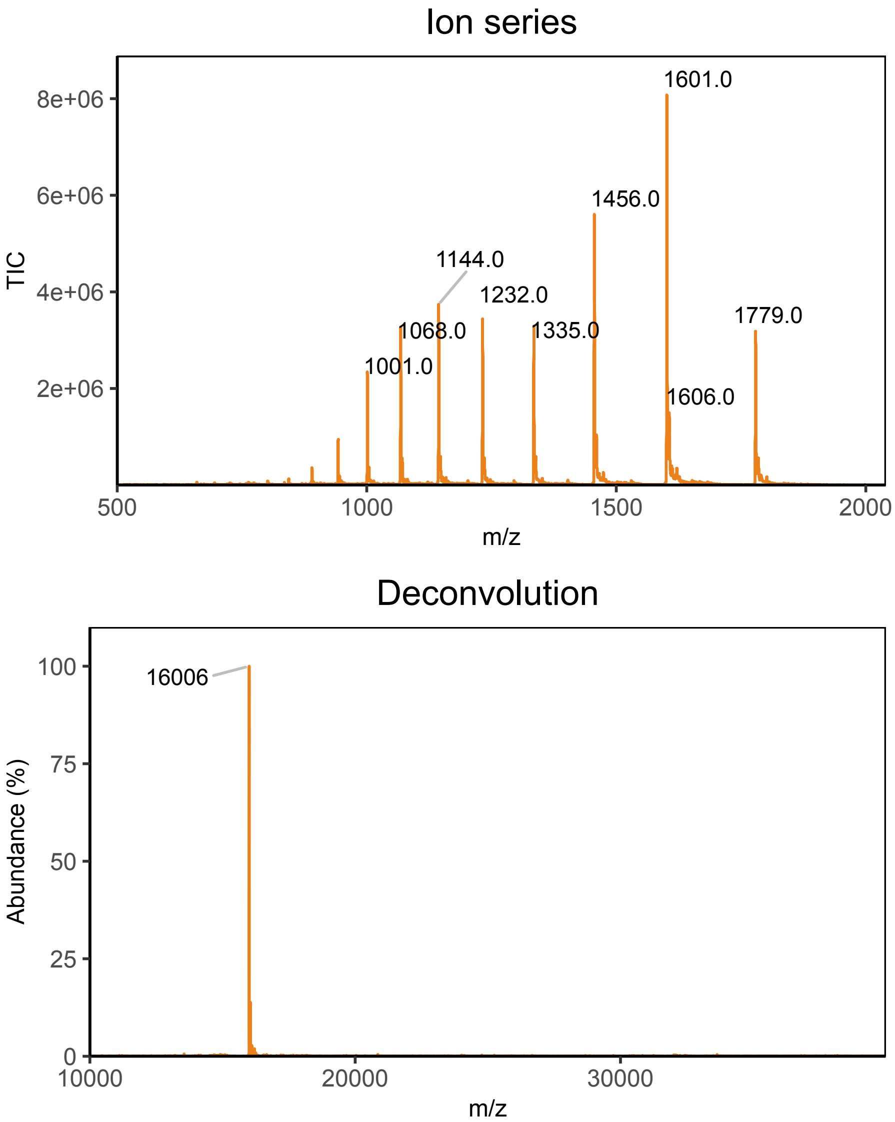

Figure S5: LC–MS spectra of C2-π; ion series and deconvoluted spectra (Calculated mass: 16006 kDa).

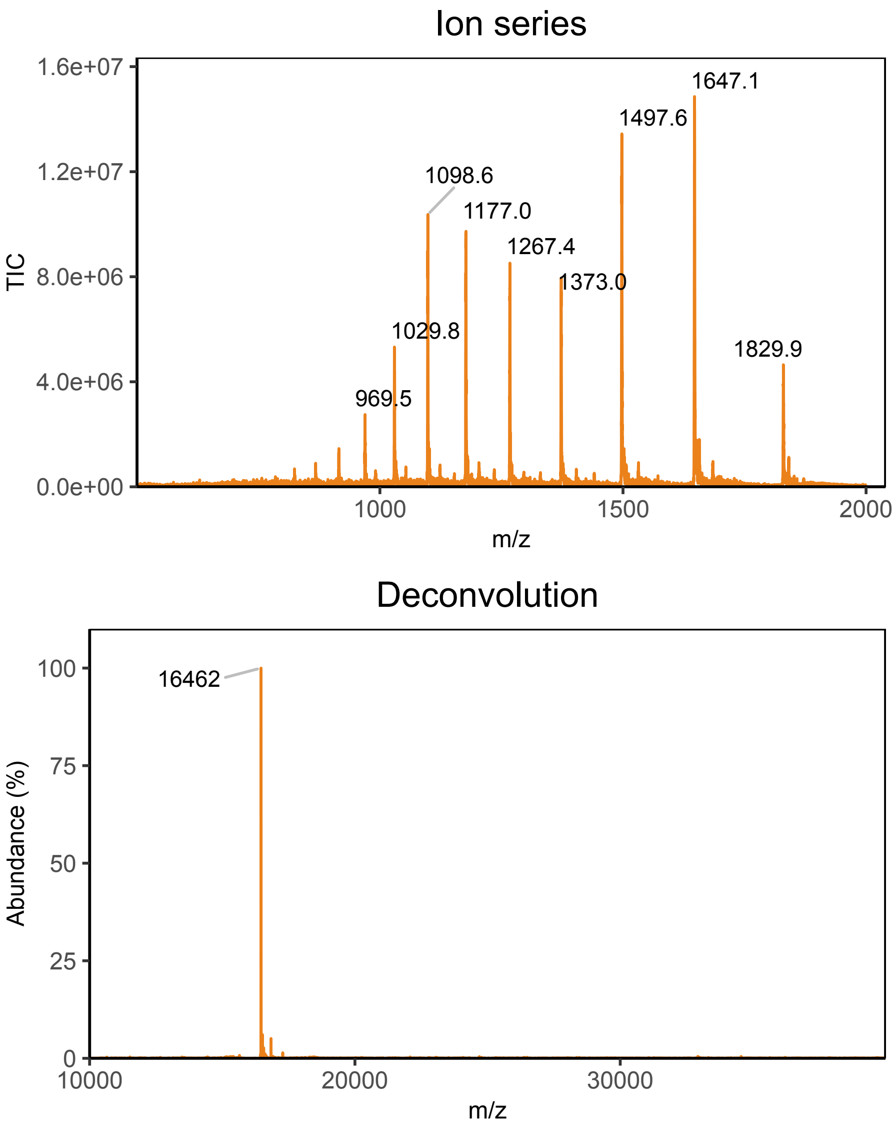

Figure S6: LC–MS spectra of C1-π-DCE; ion series and deconvoluted spectra (Calculated mass: 16464 kDa).

7.3 Test reactions

To desAbs C1 (a control devoid of a free cysteine residue), C1-DCE, C1-π, C1-ACPA and C2-π (~30 µM, ~50 µL) in NaPi (20 mM pH 8.0) or Tris-HCl (20 mM pH 8.5), a DMF solution of Linker 1 (0.5-50 equivalents, ~0.5-1% DMF, v/v) (5-50 mM stock in DMF) was added along with TCEP (20 equivalents, 20 mM stock in reaction buffer) and the reactions were left to incubate at 25 °C or 37 °C. Time points were taken at 1, 3 and 5 h (and 24 h for C1-DCE) with conversion determined by LC–MS analysis (Tables S1, S2, S3 and S4). Homodimerisation of C1-DCE (60 µM) was attempted with Linker 1 (0.5 equivalents ~0.5% DMF, v/v) (5 mM stock in DMF) in NaPi (20 mM pH 8.0) and Tris-HCl (20 mM pH 8.5) with TCEP (20 equivalents, 20 mM stock in reaction buffer) at 25 °C or 37 °C. Conversions were determined by relative percentages of total ion intesity of the devonvoluted spectra (LC–MS spectra of the final time point taken for each reaction is shown (Figures S7-S20)).

Table S1: Monomer test reactions with C1-DCE and Linker 1 at 25 °C in NaPi (20 mM pH 8.0), with conversions determined by relative ion intensity of LC–MS spectra.

Time (h)	Equivalents		
	2	10	50
	Conversion (%)		
1	-	-	-
3	-	-	<5
5	5	10	15
24	10	40	45

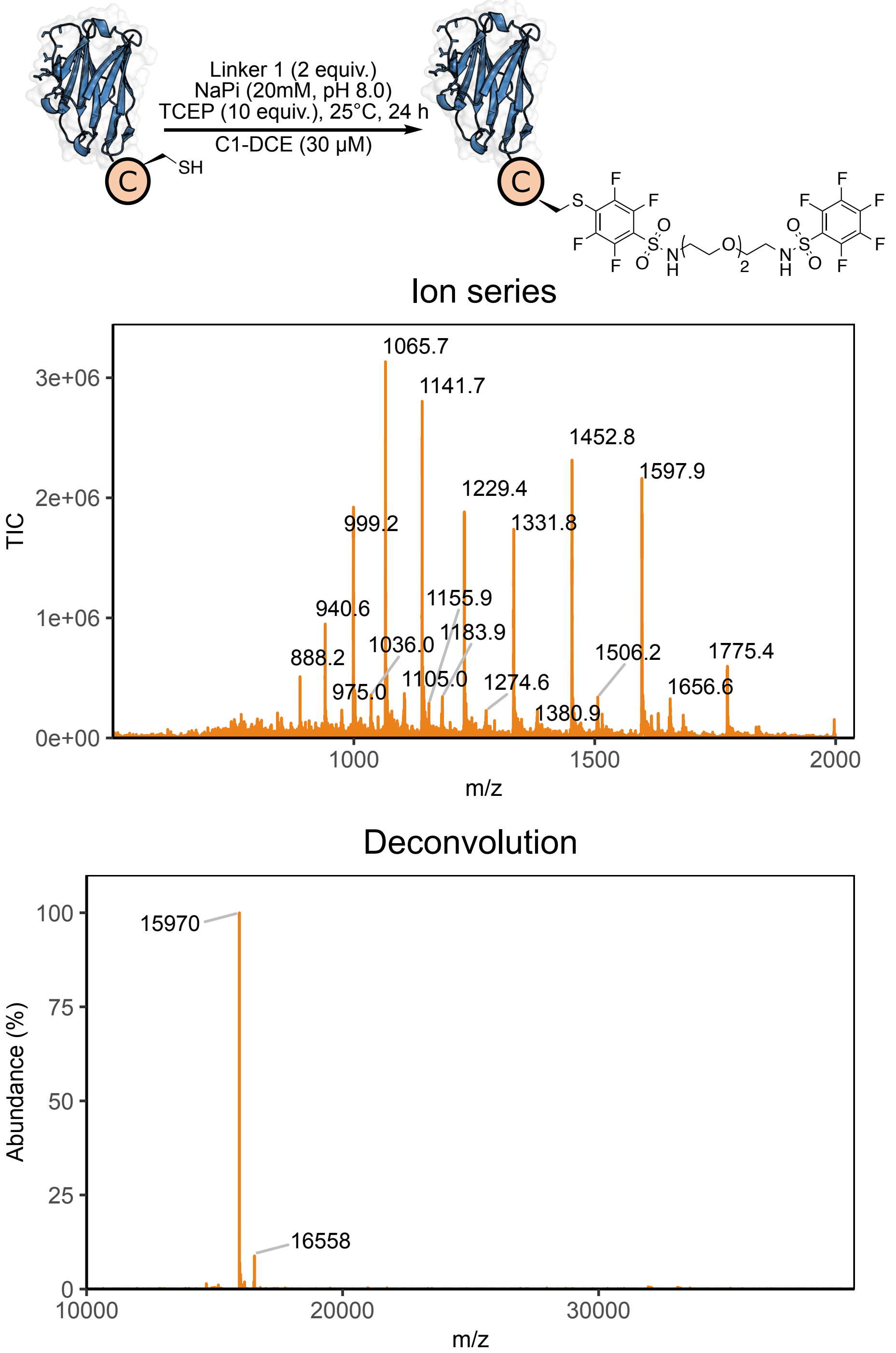

Figure S7: LC–MS spectra of C1-DCE incubated with Linker 1 (2 equivalents) at 25 °C in NaPi (20 mM pH 8.0) for 24 h; ion series and deconvoluted spectra (Calculated mass: 16557 kDa).

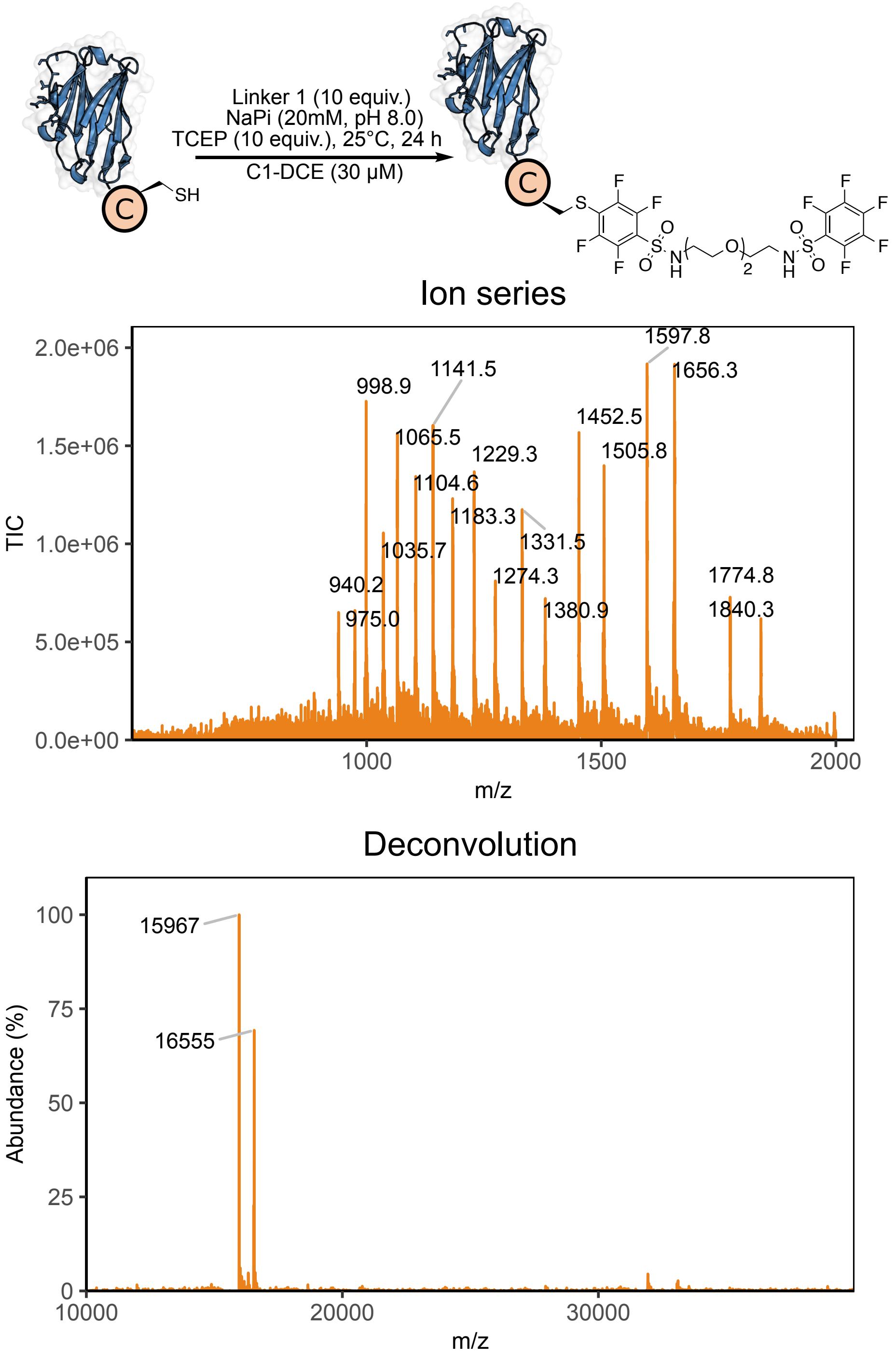

Figure S8: LC–MS spectra of C1-DCE incubated with Linker 1 (10 equivalents) at 25 °C in NaPi (20 mM pH 8.0) for 24 h; ion series and deconvoluted spectra (Calculated mass: 16557 kDa).

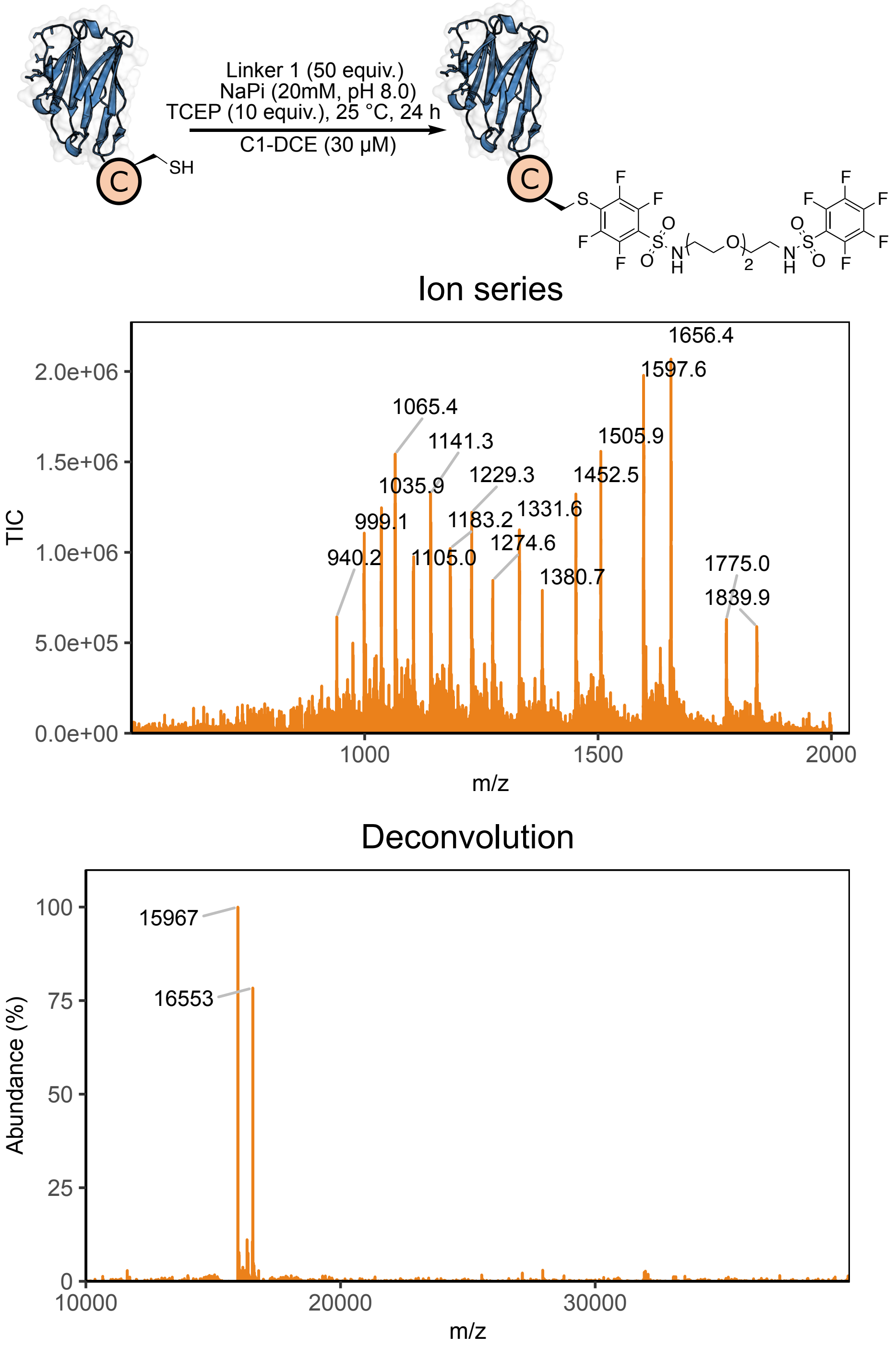

Figure S9: LC–MS spectra of C1-DCE incubated with Linker 1 (50 equivalents) at 25 °C in NaPi (20 mM pH 8.0) for 24 h; ion series and deconvoluted spectra (Calculated mass: 16557 kDa).

Table S2: Monomer test reactions with C1-DCE and Linker 1 at 25 °C and 37 °C in Tris-HCl (20 mM pH 8.5), with conversions determined by relative ion intensity of LC–MS spectra.

Time (h)	Equivalents			
	2^a	10^a	50^a	50^b
	Conversion (%)			
1	-	-	-	-
3	-	<5	10	10
5	5	10	15	15
24	15	55	60	60

[a]Reaction carried out at 25 °C. [b]Reaction carried out at 37 °C.

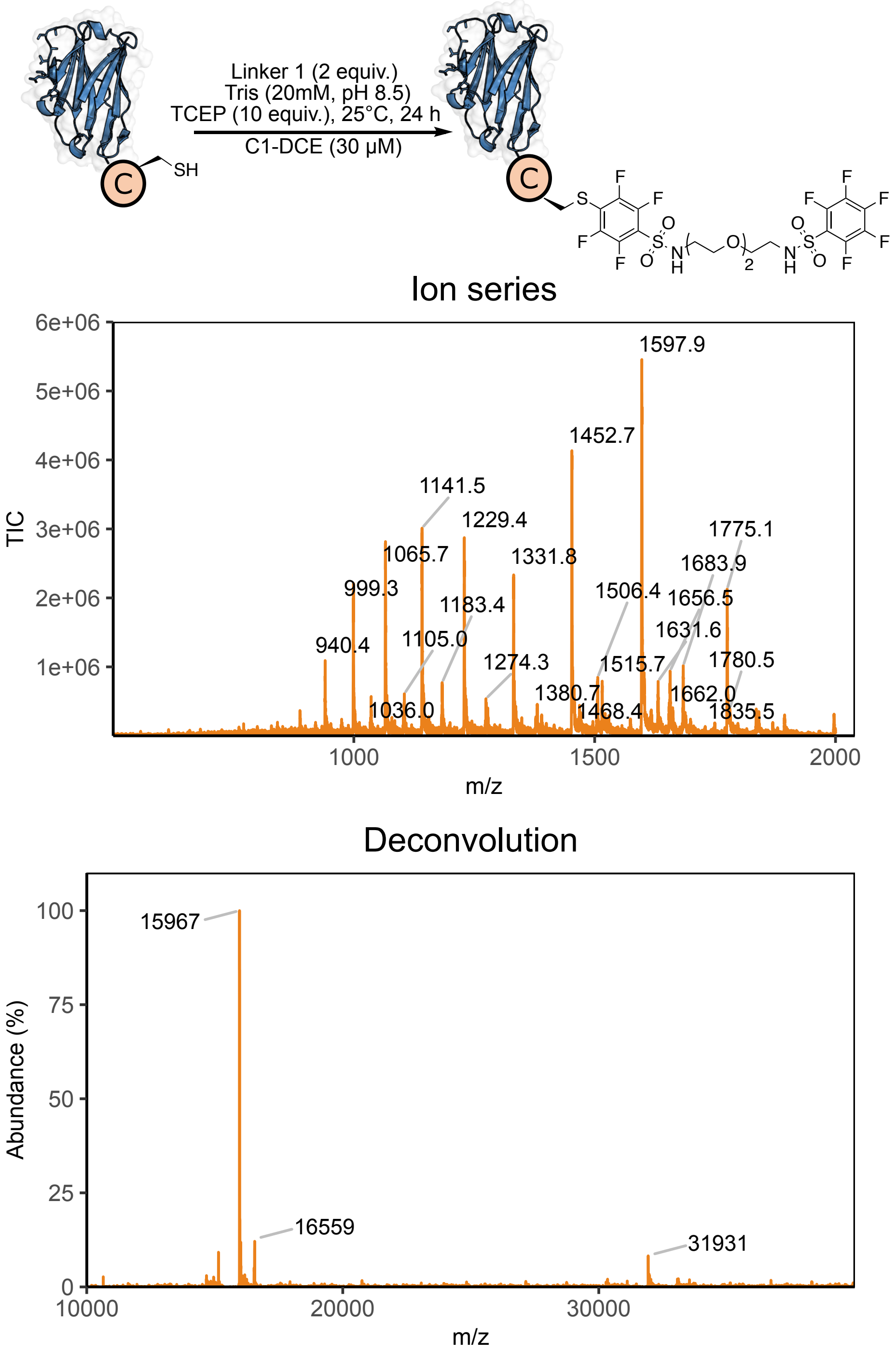

Figure S10: LC–MS spectra of C1-DCE incubated with Linker 1 (2 equivalents) at 25 °C in Tris-HCl (20 mM pH 8.5) for 24 h; ion series and deconvoluted spectra (Calculated mass: 16557 kDa).

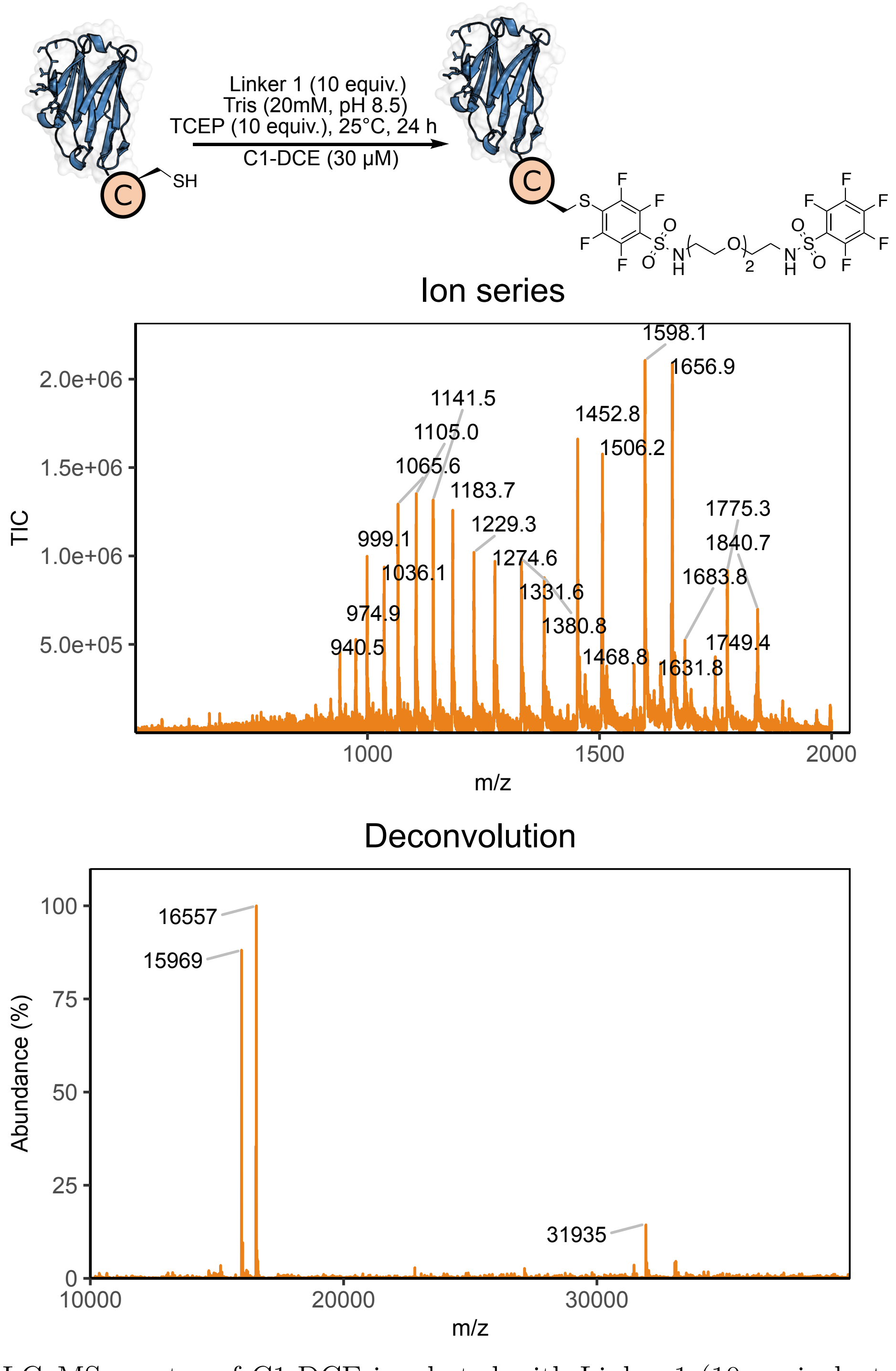

Figure S11: LC–MS spectra of C1-DCE incubated with Linker 1 (10 equivalents) at 25 °C in Tris-HCl (20 mM pH 8.5) for 24 h; ion series and deconvoluted spectra (Calculated mass: 16557 kDa).

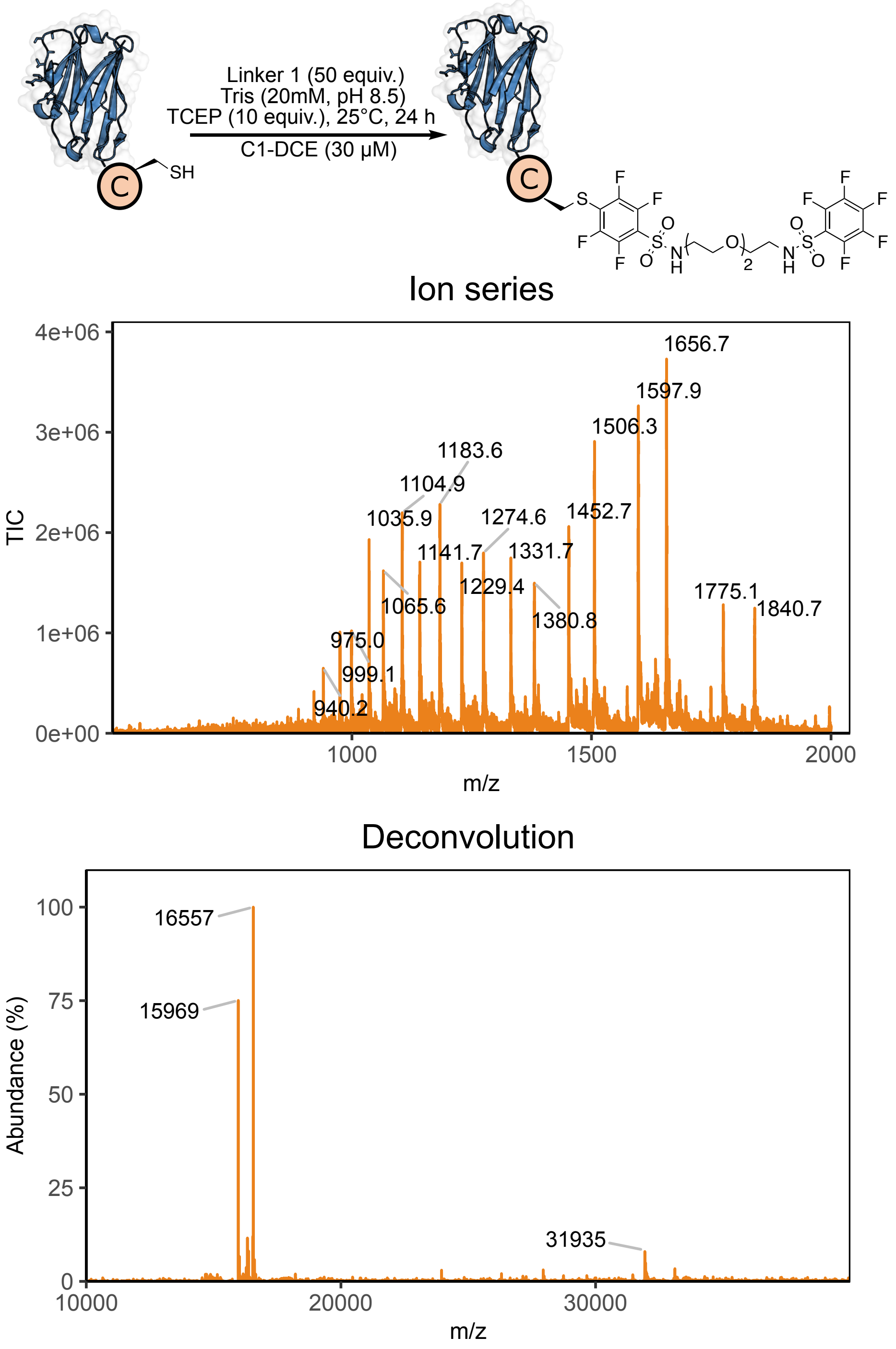

Figure S12: LC–MS spectra of C1-DCE incubated with Linker 1 (50 equivalents) at 25 °C in Tris-HCl (20 mM pH 8.5) for 24 h; ion series and deconvoluted spectra (Calculated mass: 16557 kDa).

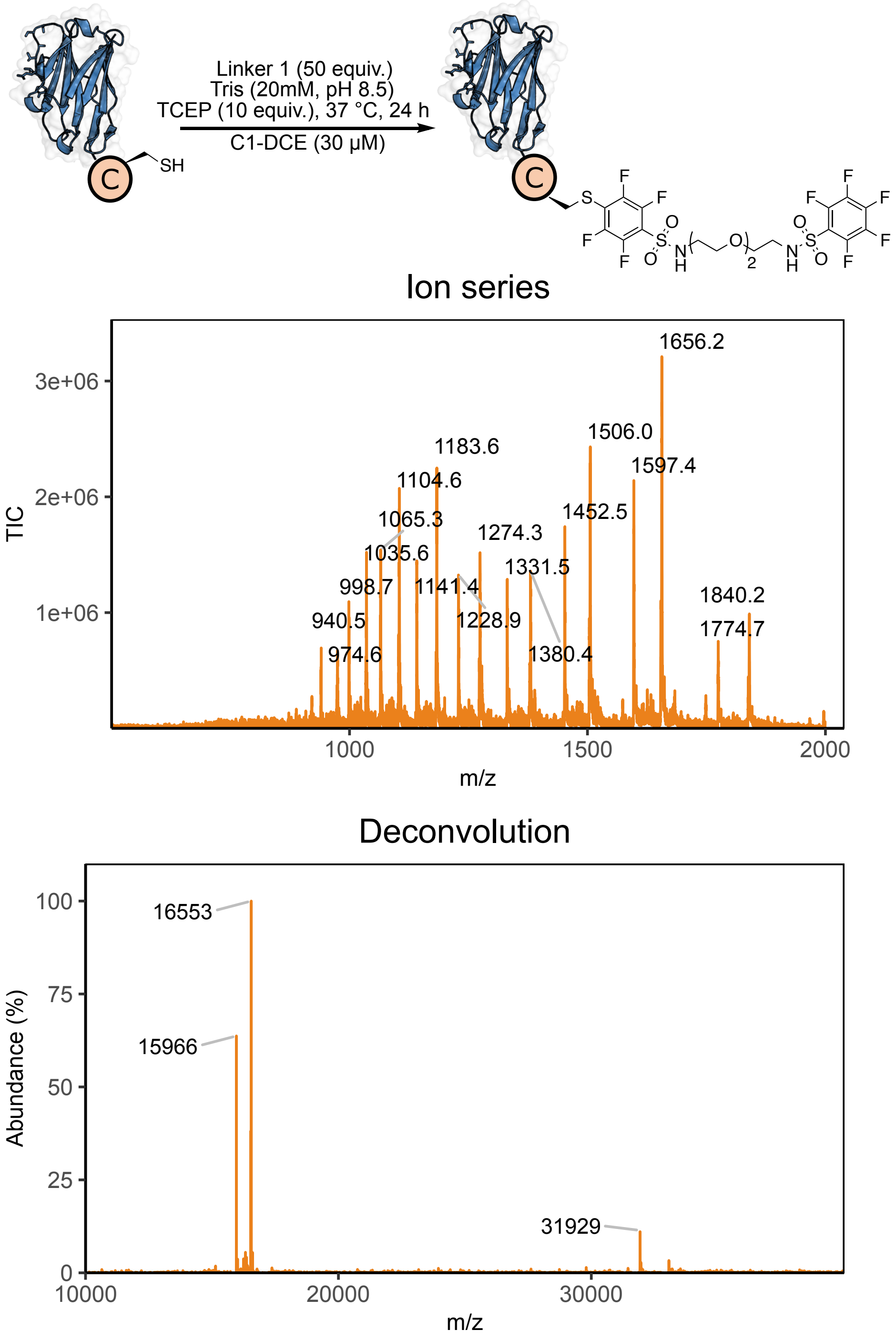

Figure S13: LC–MS spectra of C1-DCE incubated with Linker 1 (50 equivalents) at 37 °C in Tris-HCl (20 mM pH 8.5) for 24 h; ion series and deconvoluted spectra (Calculated mass: 16557 kDa).

Table S3: Dimer test reactions with C1-DCE and 0.5 equivalents Linker 1 in Tris-HCl (20 mM pH 8.5), with conversions determined by relative ion intensity of LC–MS spectra.

Time (h)	Temperature (°C)	
	25	37
	Conversion (%)	
1	-	-
3	-	-
5	-	-
24[a]	<10	<20

[a]%conversion to C1-DCE–L1 monomer (no dimer formation was observed).

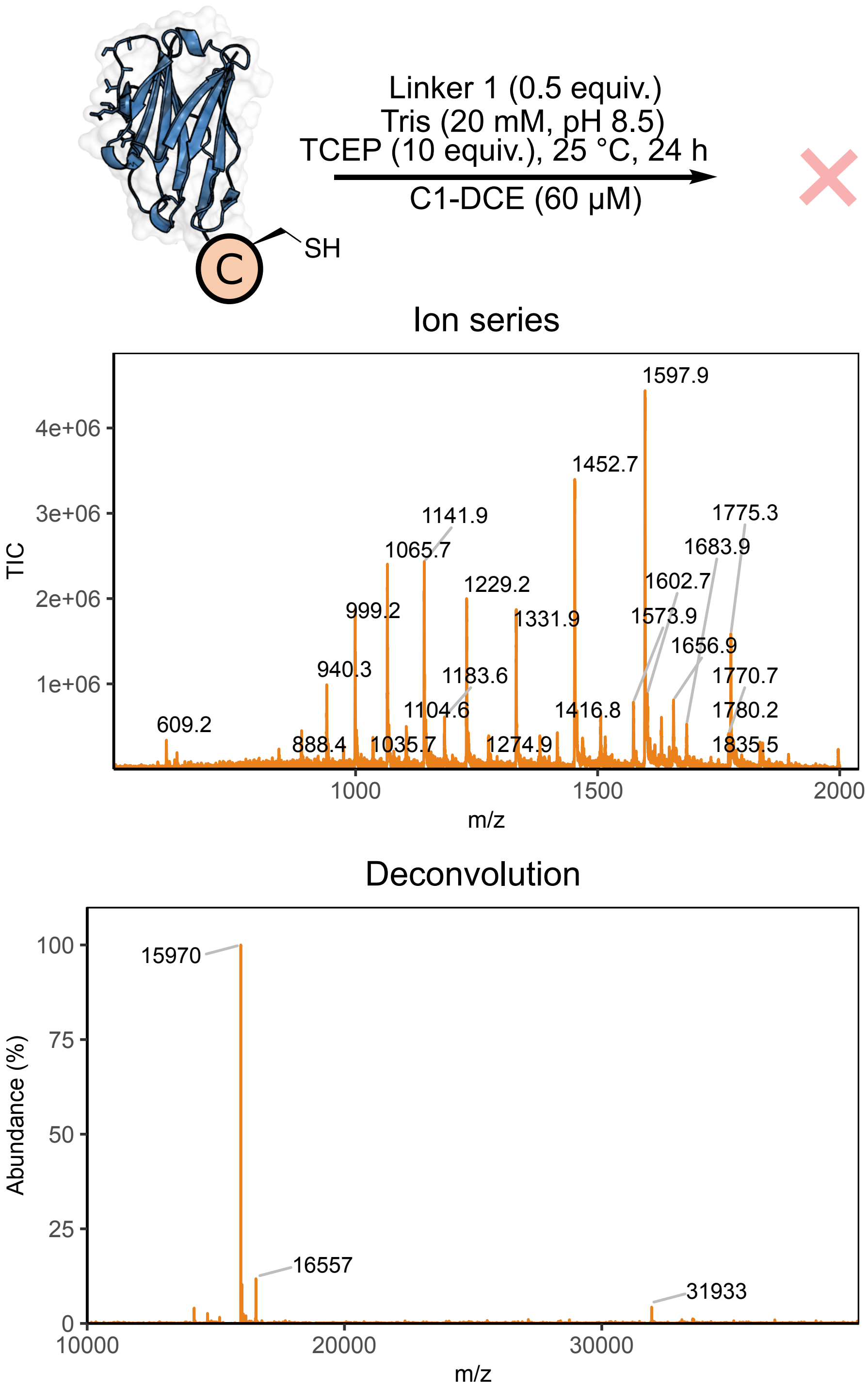

Figure S14: LC–MS spectra of C1-DCE incubated with Linker 1 (0.5 equivalents) at 25 °C in Tris-HCl (20 mM pH 8.5) for 24 h; ion series and deconvoluted spectra (Calculated mass: 32506 kDa).

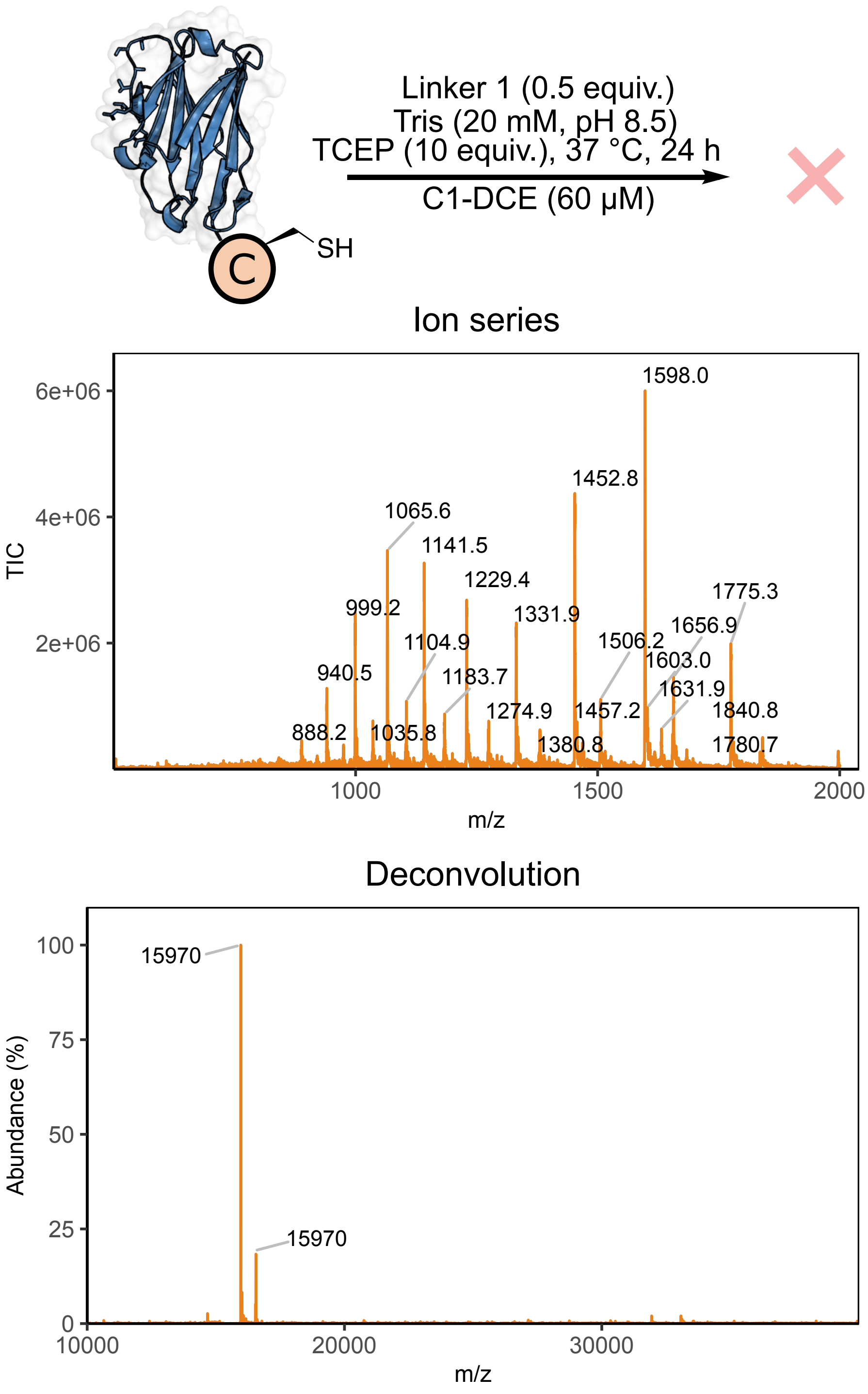

Figure S15: LC–MS spectra of C1-DCE incubated with Linker 1 (0.5 equivalents) at 37 °C in Tris-HCl (20 mM pH 8.5) for 24 h; ion series and deconvoluted spectra (Calculated mass: 32506 kDa).

Table S4: Monomer test reactions with C1-π and C1-ACPA desAbs at 25 °C in NaPi (20 mM pH 8.0) or Tris-HCl (20 mM pH 8.5) and 10 equivalents of Linker 1, with conversions determined by relative ion intensity of LC–MS spectra.

DesAb	Conversion (%)		
	Time (h)		
	1	3	5
C1-π	60	>95	>99
C2-π	60	>95	>99
C1-ACPA[a]	5	10	20
C1-ACPA[b]	5	10	25

[a] NaPi (20 mM pH 8.0) [b] Tris-HCl (20 mM pH 8.5).

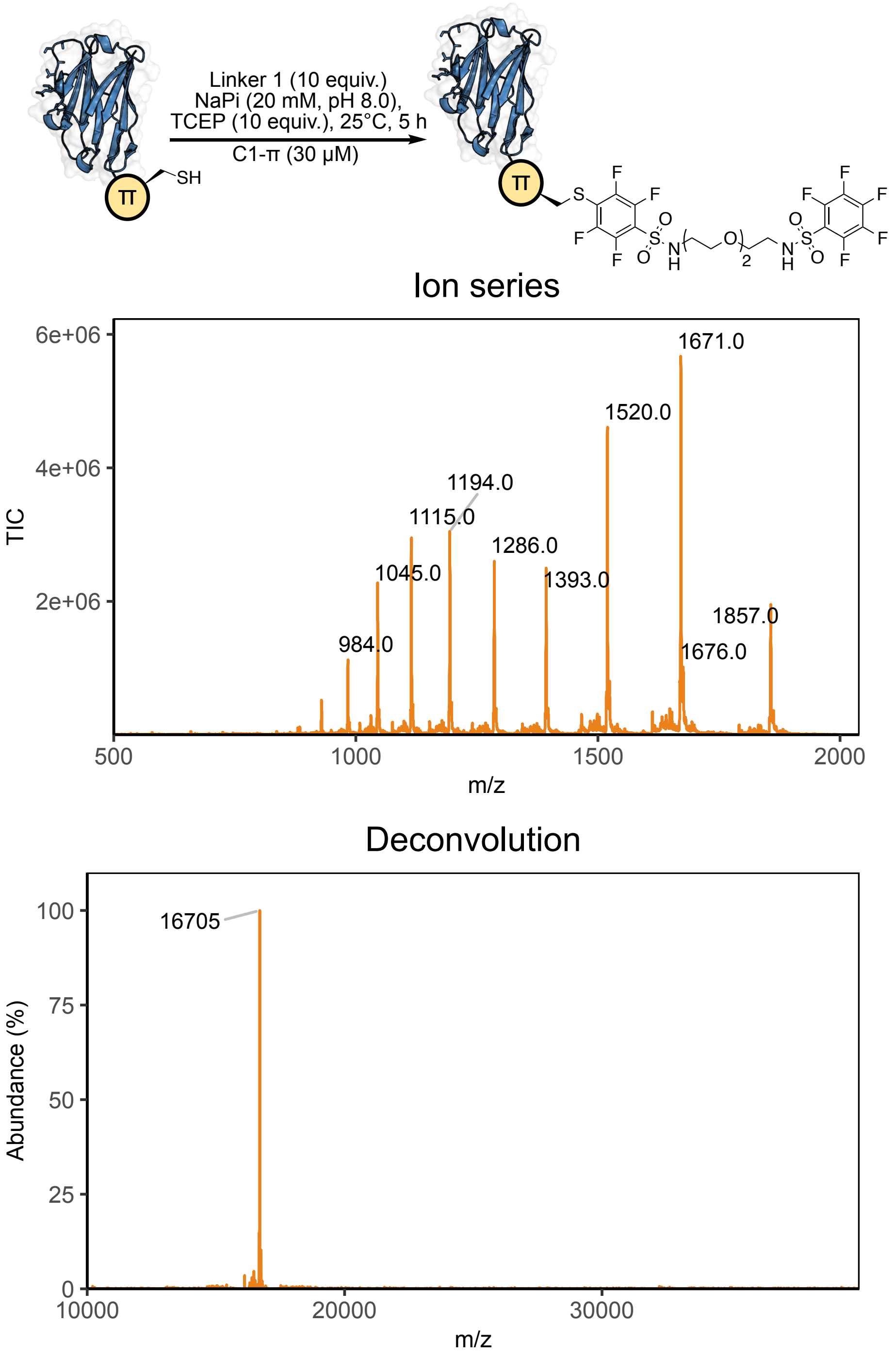

Figure S16: LC–MS spectra of C1-π incubated with Linker 1 (10 equivalents) at 25 °C in NaPi (20 mM pH 8.0) for 5 h; ion series and deconvoluted spectra (Calculated mass: 16705 kDa).

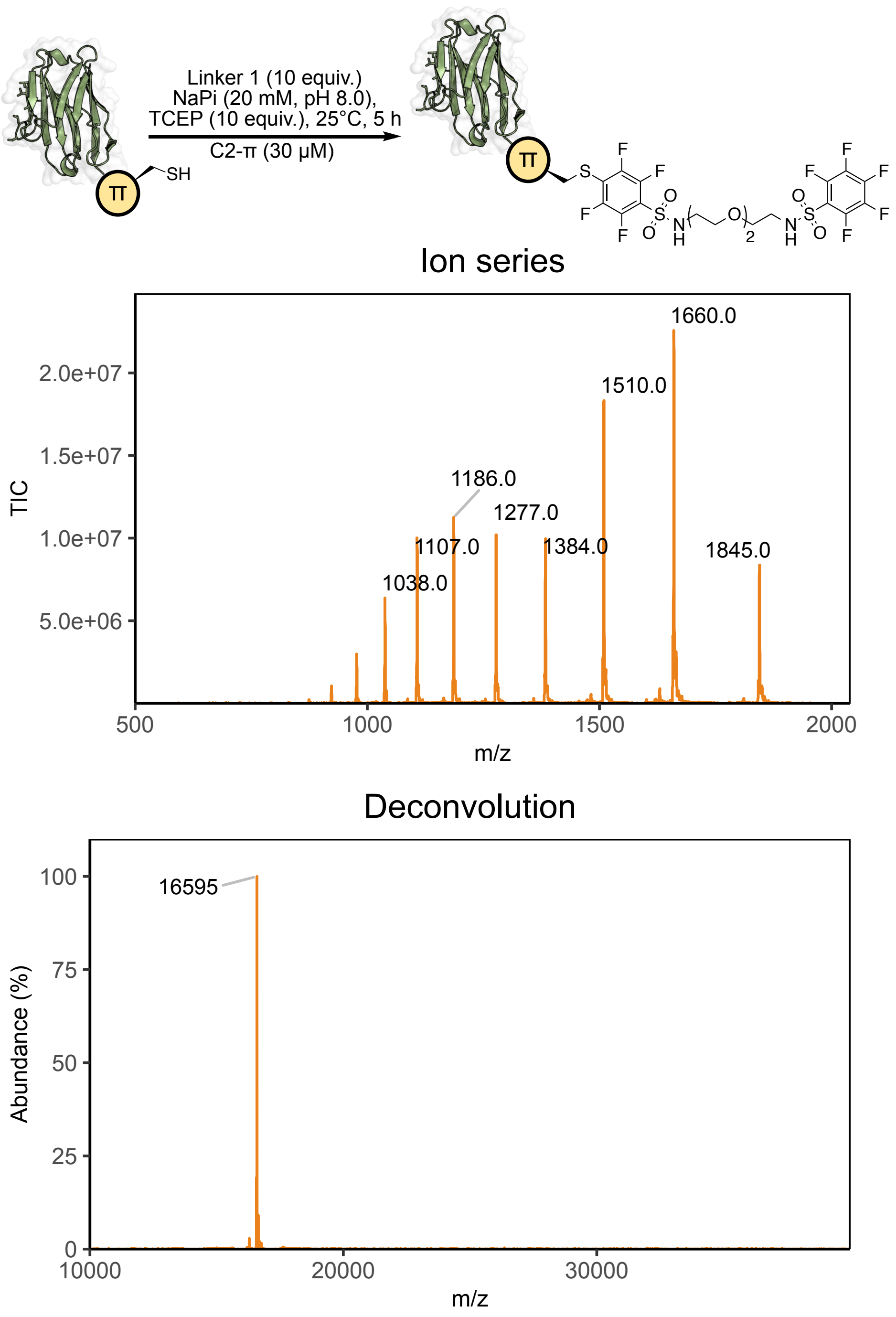

Figure S17: LC–MS spectra of C2-π incubated with Linker 1 (10 equivalents) at 25 °C in NaPi (20 mM pH 8.0) for 5 h; ion series and deconvoluted spectra (Calculated mass: 16594 kDa).

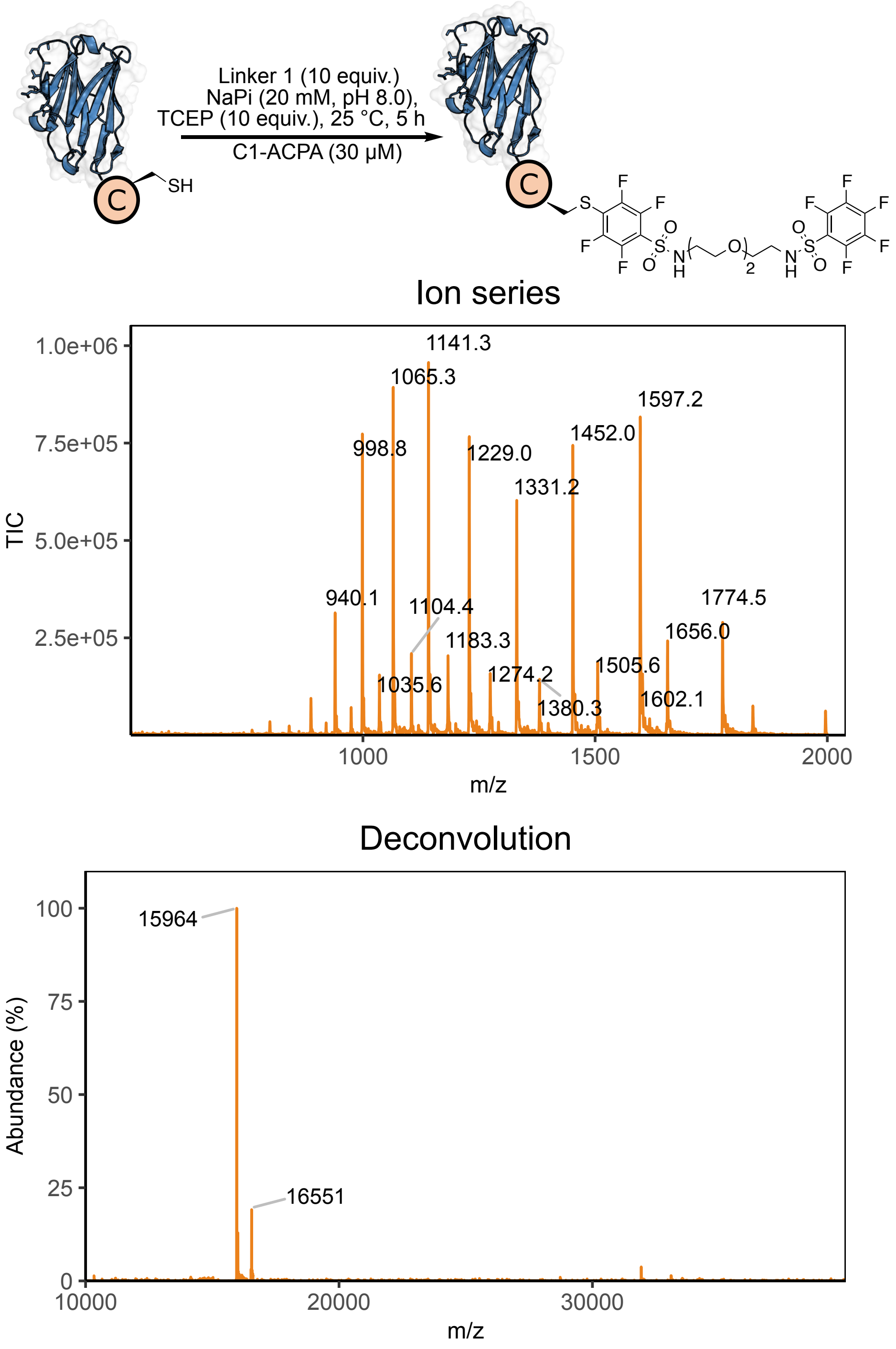

Figure S18: LC–MS spectra of C1-ACPA incubated with Linker 1 (10 equivalents) at 25 °C in NaPi (20 mM pH 8.0) for 5 h; ion series and deconvoluted spectra (Calculated mass: 16552 kDa).

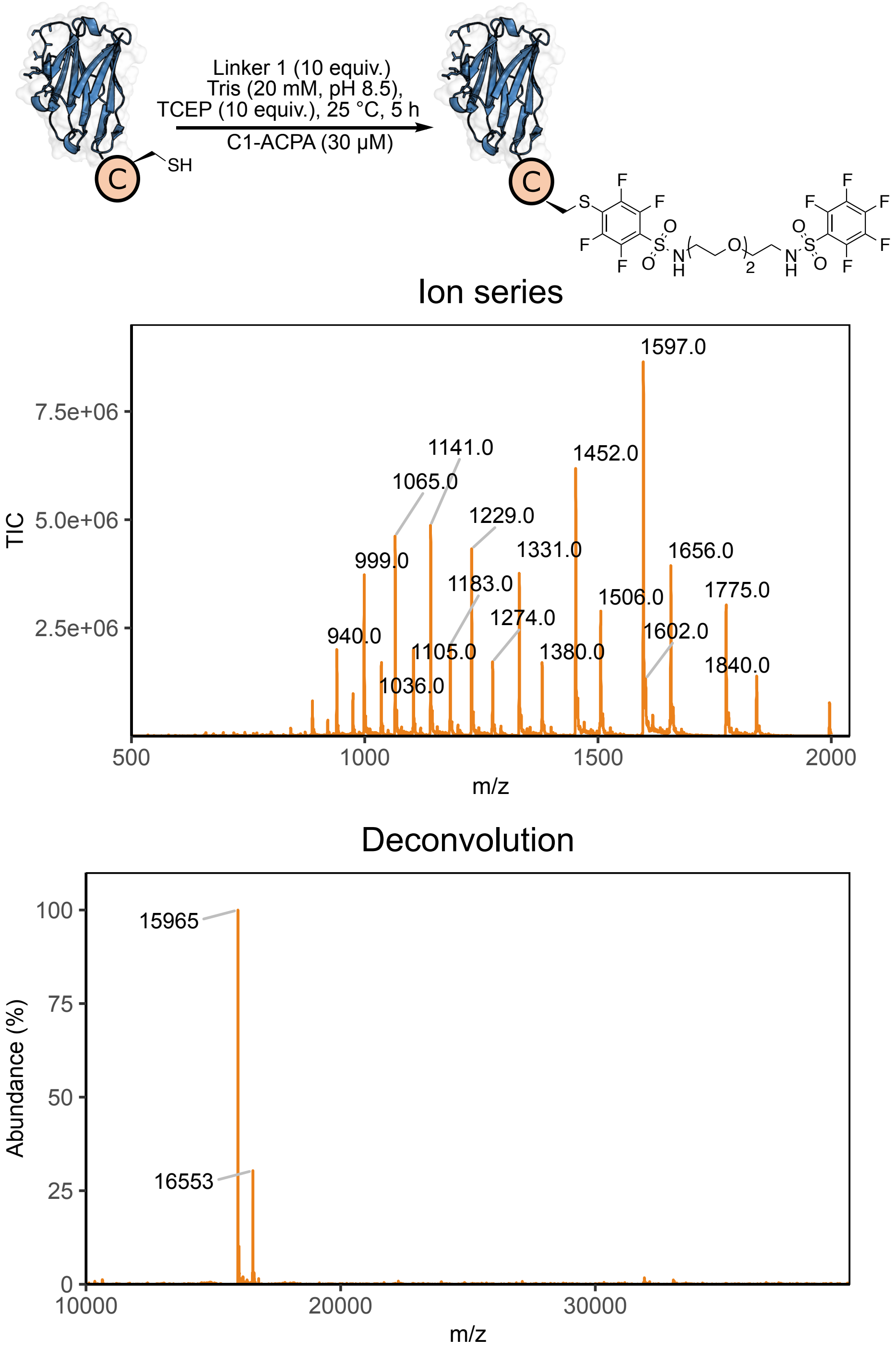

Figure S19: LC–MS spectra of C1-ACPA incubated with Linker 1 (10 equivalents) at 25 °C in Tris-HCl (20 mM pH 8.5) for 5 h; ion series and deconvoluted spectra (Calculated mass: 16552 kDa).

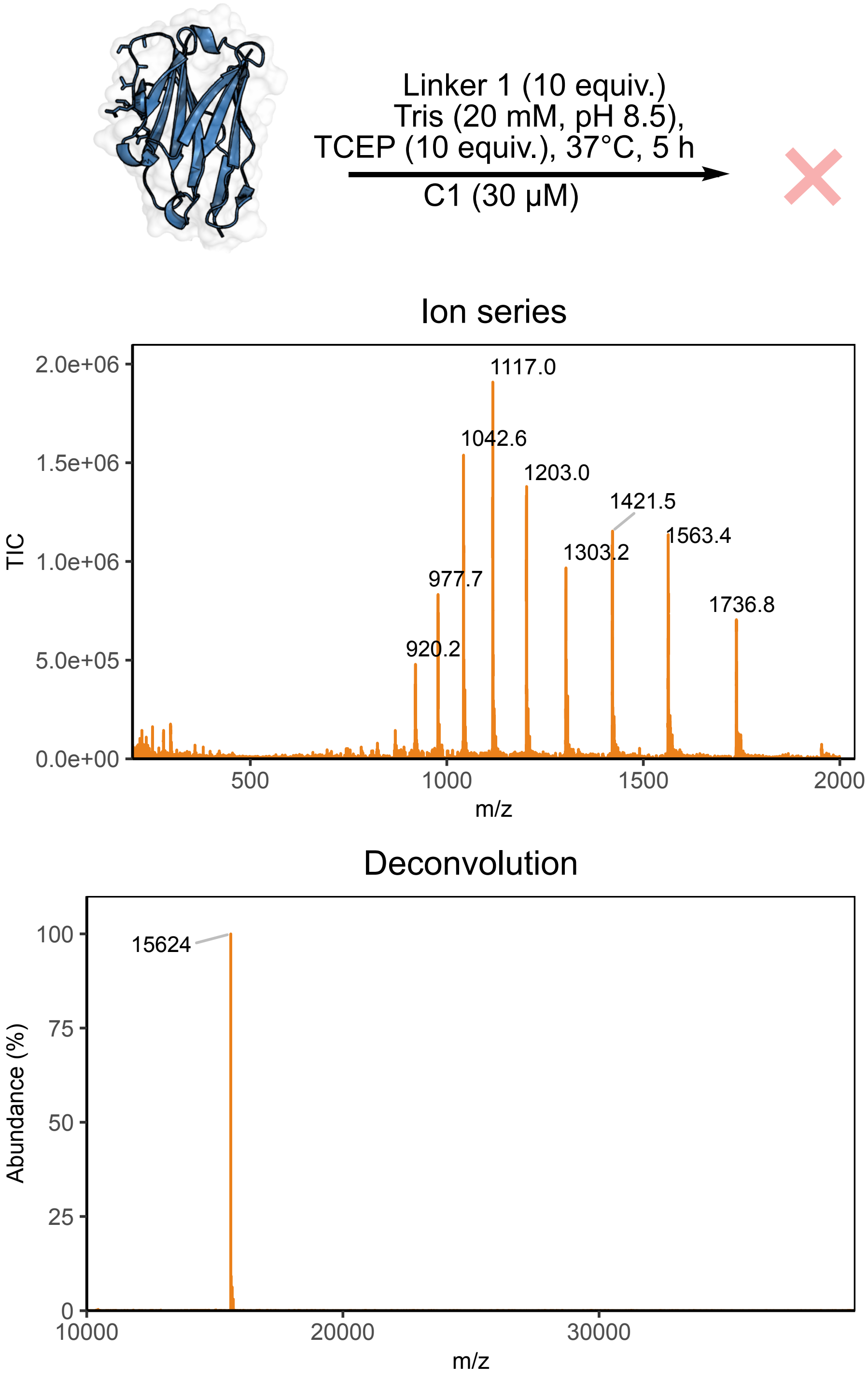

Figure S20: LC–MS spectra of C1 control antibody (no free cysteine) incubated with Linker 1 (10 equivalents) at 37 °C in Tris-HCl (20 mM pH 8.5) for 24 h; ion series and deconvoluted spectra (Calculated mass: 15622 kDa). Confirmation that a solvent exposed engineered cysteine residue is essential for conjugation using a PFP-sulfonamide functionality.

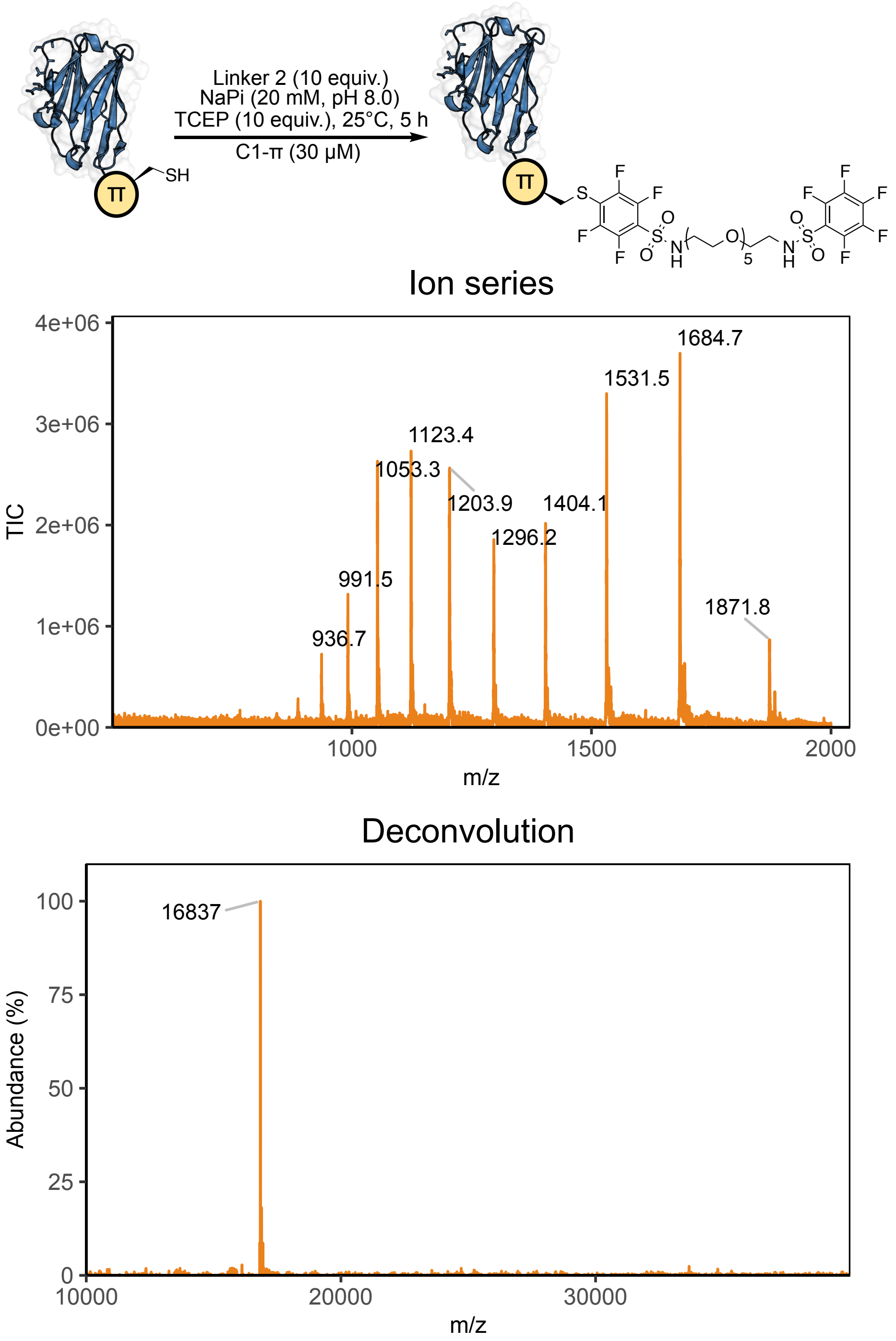

Figure S21: LC–MS spectra of C1-π incubated with Linker 2 (20 equivalents) at 25 °C in NaPi (20 mM pH 8.0) for 5 h; ion series and deconvoluted spectra (Calculated mass: 16837 kDa).

7.4 General homodimerisation procedure

To desAbs (~60-70 µM, ~200 µL) in NaPi (20 mM pH 8.0), a DMF solution of Linker 1 or Linker 2 (0.5 equivalents, ~0.5% DMF, v/v) (5 mM stock in DMF) was added along with TCEP (20 equivalents, 20 mM stock in reaction buffer) and the reactions were left to incubate at 25 °C for 24 h. Time points were taken at 0, 1, 3, 5, 8 and 24 h, and immediately flash frozen in liquid N_2. Samples were analysed by SDS–PAGE to observe conversion from monomer to dimer over this time period (Figure S22) and by LC–MS at 24 h (Figures S23-S26. Purification was carried out by SEC using a HiLoad 10/300 Superdex 75 pg column (Cytiva, Little Chalfont, UK) and PBS (pH 7.4) as an elution buffer. Dimer containing fractions were combined with concentration and corresponding isolated yields calculated using absorbance at 280 nm (Table S5). Purity was confirmed by SDS–PAGE (Figure S22) and LC–MS analyses (Figures S29-S31).

Table S5: Homodimerisation reaction conditions and isolated yields after SEC.

DesAb	Reaction conditions		Yield		%Yield
	Conc. (µM)	Vol. (µL)	Conc. (µM)	Vol. (µL)	
C1-ACPA (L1)	57 µM	220 µL	-	-	0
C1C1-L1 (C1-π)	60 µM	220 µL	2.44 µM	1500 µL	55
C1C1-L2 (C1-π)	60 µM	191 µL	1.93 µM	1500 µL	51
C2C2-L2 (C2-π)	71 µM	175 µL	1.31 µM	1500 µL	32

7.5 Heterodimerisation of C1 and C2

To desAb C1-π (28.15 µM, 600 µL) in NaPi (20 mM pH 8.0), a DMF solution of Linker 2 (6.76 µL, 20 equivalents, ~1% DMF, v/v) (50 mM, stock in DMF) was added along with TCEP (8.45 µL, 20 equivalents, 20 mM stock in 20 mM NaPi pH 8.0) and the reaction left to incubate at 25 °C. Reaction progress was monitored by LC–MS analysis until completion, around 4 h (Figure S21). Purification was carried out by SEC using a HiLoad 10/300 Superdex 75 pg column (Cytiva, Little Chalfont, UK) and NaPi (20 mM pH 8.0) as the elution buffer to afford pure modified monomer (5.36 µM, 1.5 mL, 48%). The linker presenting monomer (C1-L2) was concentrated to 73 µM and to 100 µL of C1-L2, C2-π was added

(51 µM, 171 µL, ~1.2 equivalents) (total protein concentration ~60 µM) along with TCEP (5 µL, 10 equivalents, 20 mM stock in 20 mM NaPi pH 8.0) (20 mM stock in 20 mM, NaPi pH 8.0) and the reaction was incubated at 25 °C for 24 h. Time points were taken at 0, 1, 3, 5, 8 and 24 h, and immediately flash frozen in liquid N_2. Samples were analysed by SDS–PAGE to observe conversion from monomer to dimer over this time period (Figure S22) and by LC–MS at 24 h (Figure S27). Purification was carried out by SEC using a HiLoad 10/300 Superdex 75 pg column (Cytiva, Little Chalfont, UK) and PBS (pH 7.4) as an elution buffer. Dimer containing fractions were combined and concentration calculated using absorbance at 280 nm (1.77 µM, 1.5 mL, 36%) (Overall yield: 17%). Purity was confirmed by SDS–PAGE (Figure S22) and LC–MS analyses (Figure S32). Additionally, following the same protocol as described above, a control reaction in which C1-DCE was added to C1-L2 (30 µM) of each) (Total protein concentration ~60 µM) along with TCEP (5 µL, 10 equivalents, 20 mM stock in 20 mM NaPi pH 8.0). As expected, no dimer linked by the PFP-sulfonamide ligand was generated under these conditions (Figure S28)

7.6 Homodimerisation using bis-maleimide linker

To C1-π (78.6 µM, 250 µL) in NaPi (20 mM pH 8.0), a DMF solution of 1,11-bismaleimido-triethyleneglycol (Thermofisher, catalogue number: 22337) (0.5 equivalents, ~0.5% DMF, v/v) (5 mM, stock in DMF) was added and the reaction was left to incubate at 25 °C for 8 h. Purification was carried out by SEC using a HiLoad 10/300 Superdex 75 pg column (Cytiva, Little Chalfont, UK) and PBS (pH 7.4) as an elution buffer. Dimer containing fractions were combined and concentration calculated using absorbance at 280 nm (2.50 µM, 1.5 mL, Isolated yield: 38%). Purity after SEC was confirmed by LC–MS analysis (Figure S33).

7.7 SDS–PAGE and LC–MS analysis of homo- and heterodimerisation reactions

7.7.1 SDS–PAGE analysis

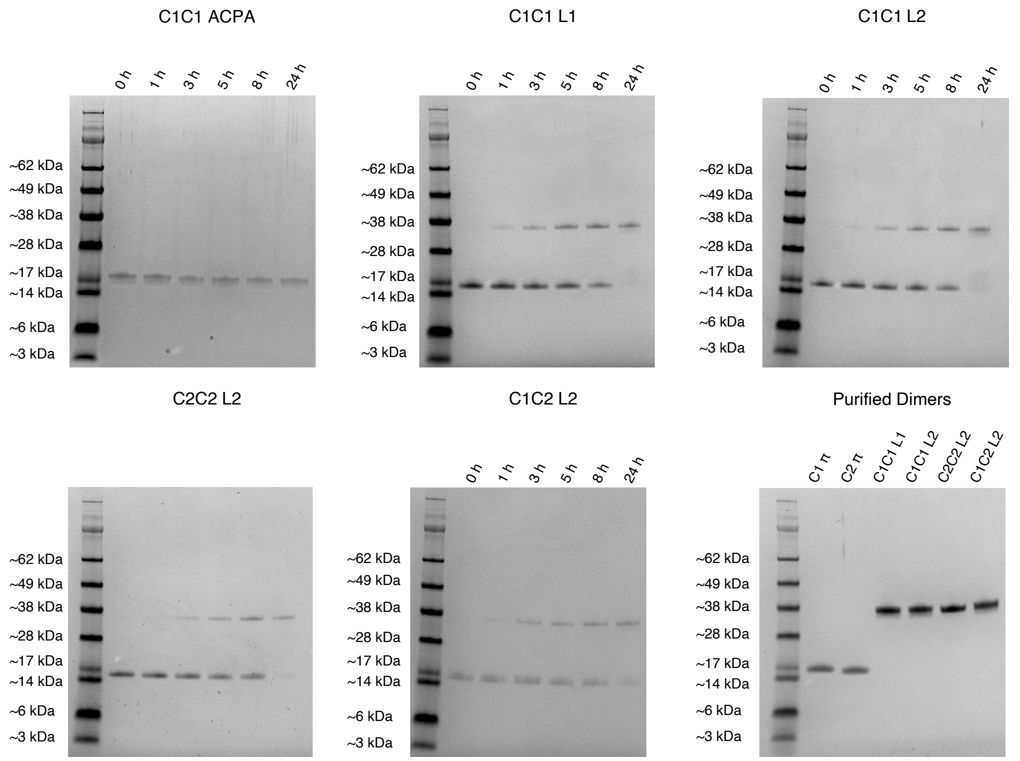

Figure S22: SDS–PAGE analysis of homo- and heterodimerisation reactions over 24h for C1-ACPA (Linker 1), C1C1-L1, C1C1-L2, C2C2-L2, C1C2-L2 and monomers and dimers purified by SEC.

7.7.2 LC–MS analysis after 24 h incubation

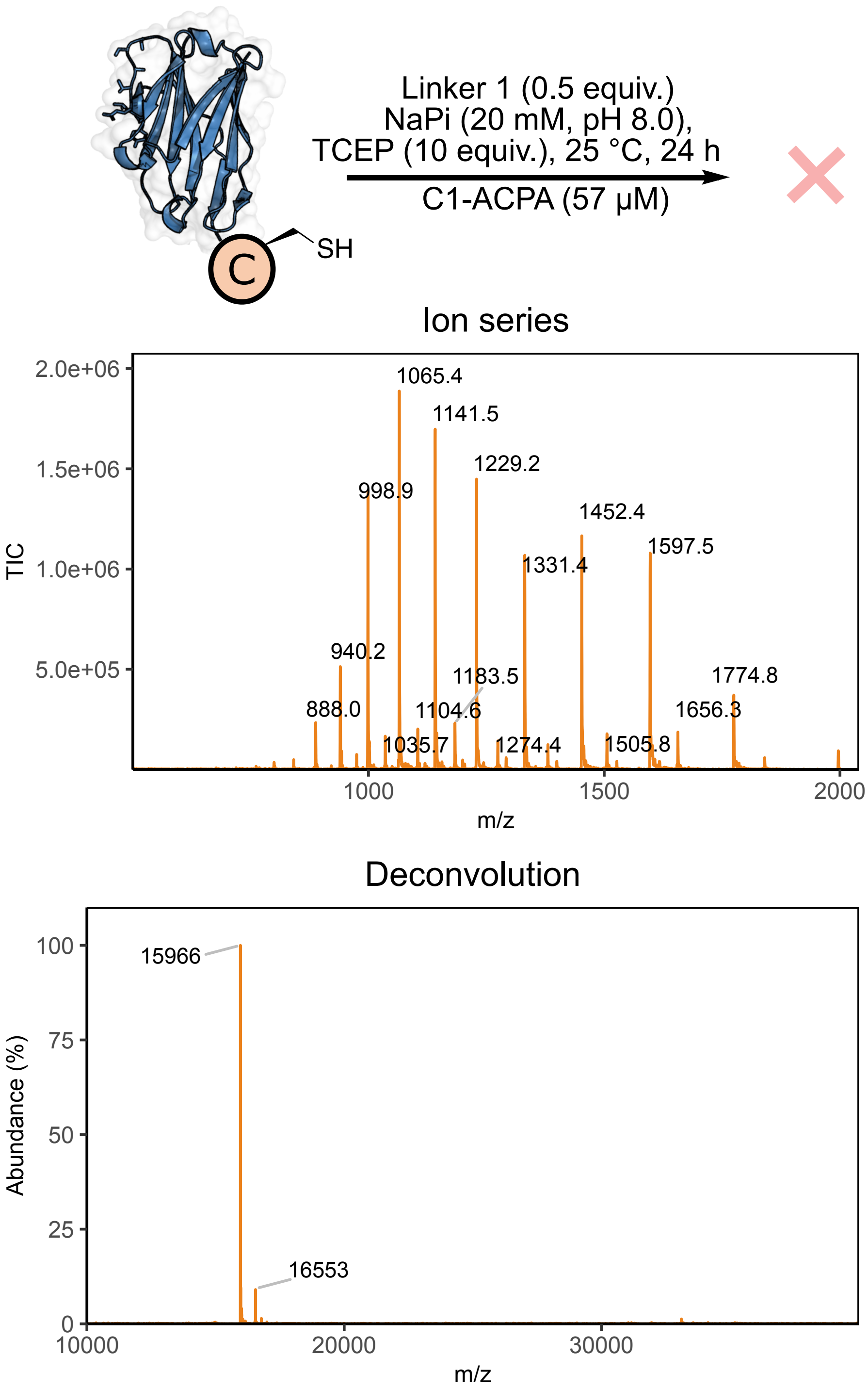

Figure S23: LC–MS spectra of C1-ACPA incubated with Linker 1 (0.5 equivalents) at 25 °C in NaPi (20 mM pH 8.0) for 24 h; ion series and deconvoluted spectra (Calculated mass: 32496 kDa).

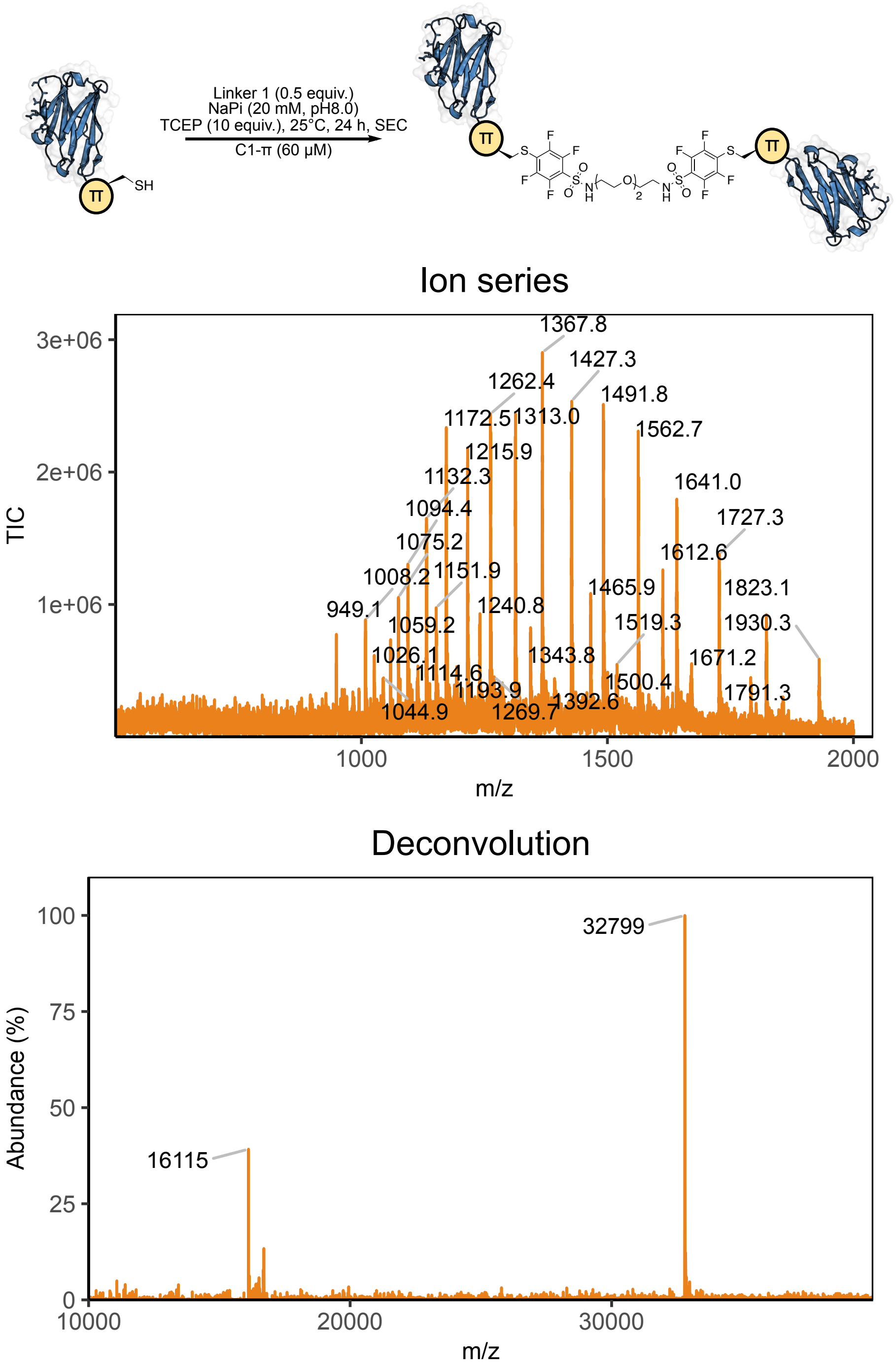

Figure S24: LC–MS spectra of C1-π incubated with Linker 1 (0.5 equivalents) at 25 °C in NaPi (20 mM pH 8.0) for 24 h; ion series and deconvoluted spectra (Calculated mass: 32801 kDa).

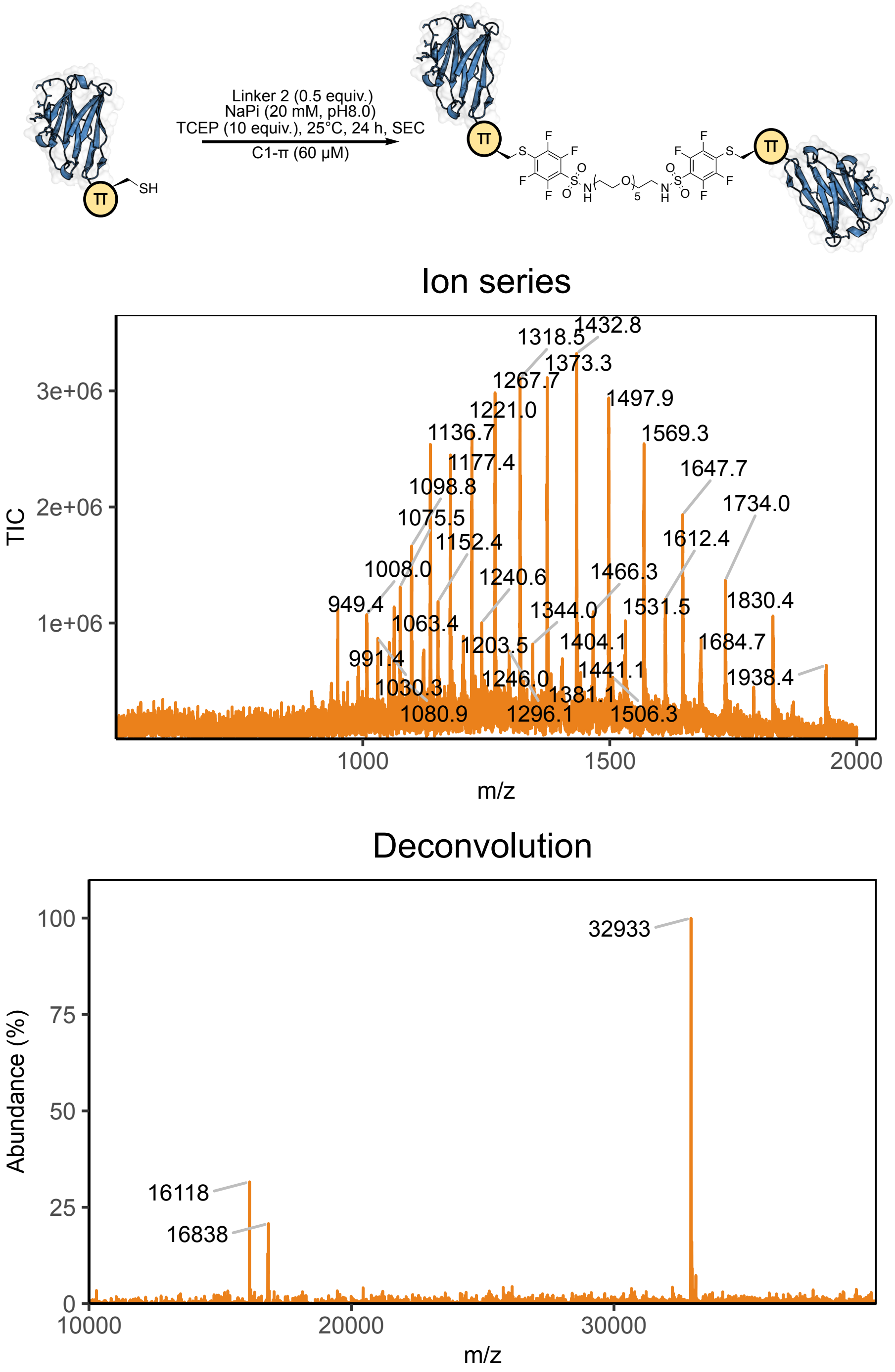

Figure S25: LC–MS spectra of C1-π incubated with Linker 2 (0.5 equivalents) at 25 °C in NaPi (20 mM pH 8.0 for 24 h; ion series and deconvoluted spectra (Calculated mass: 32934 kDa).

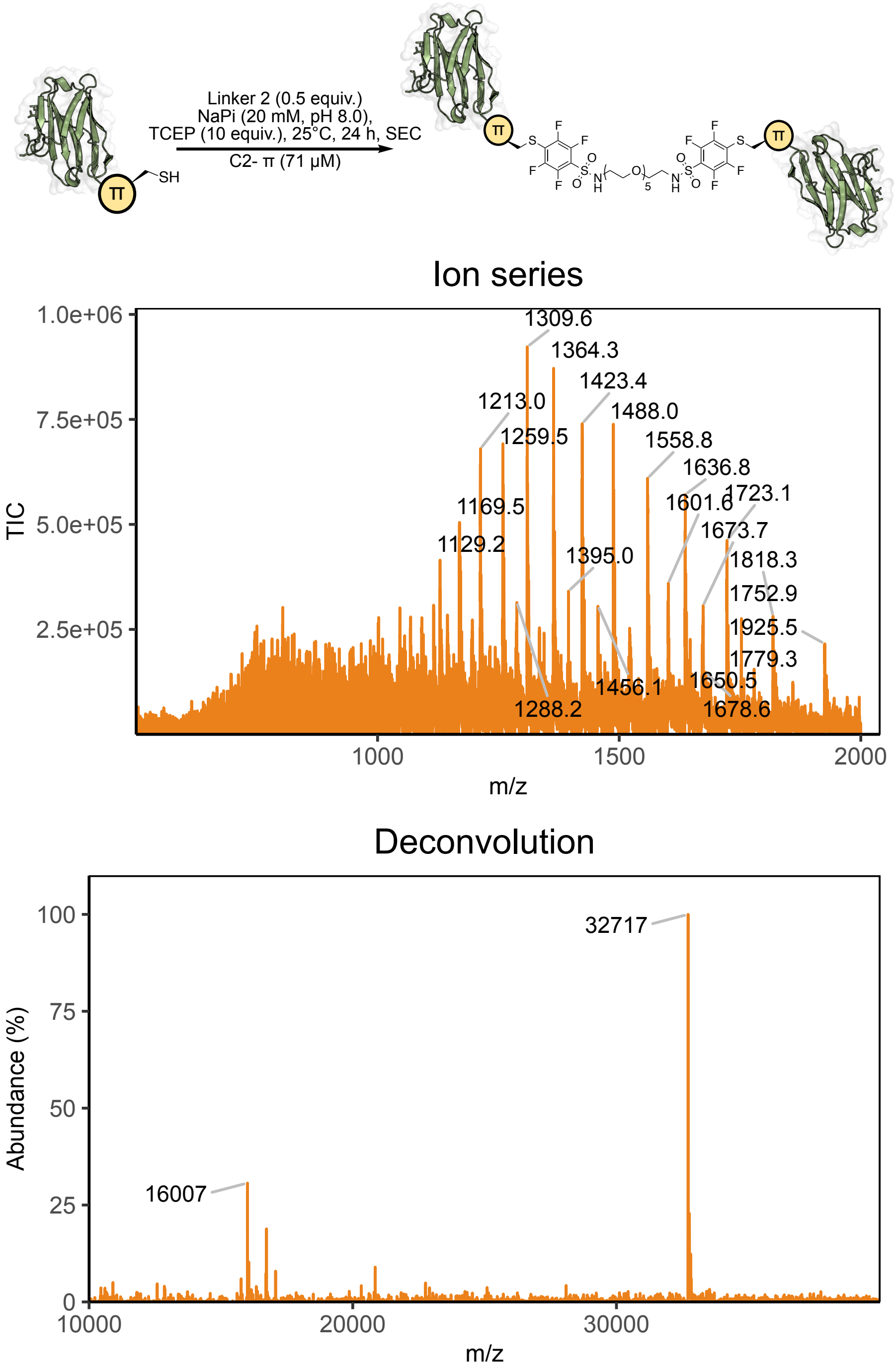

Figure S26: LC–MS spectra of C2-π incubated with Linker 2 (0.5 equivalents) at 25 °C in NaPi (20 mM pH 8.0 for 24 h; ion series and deconvoluted spectra (Calculated mass: 32716 kDa).

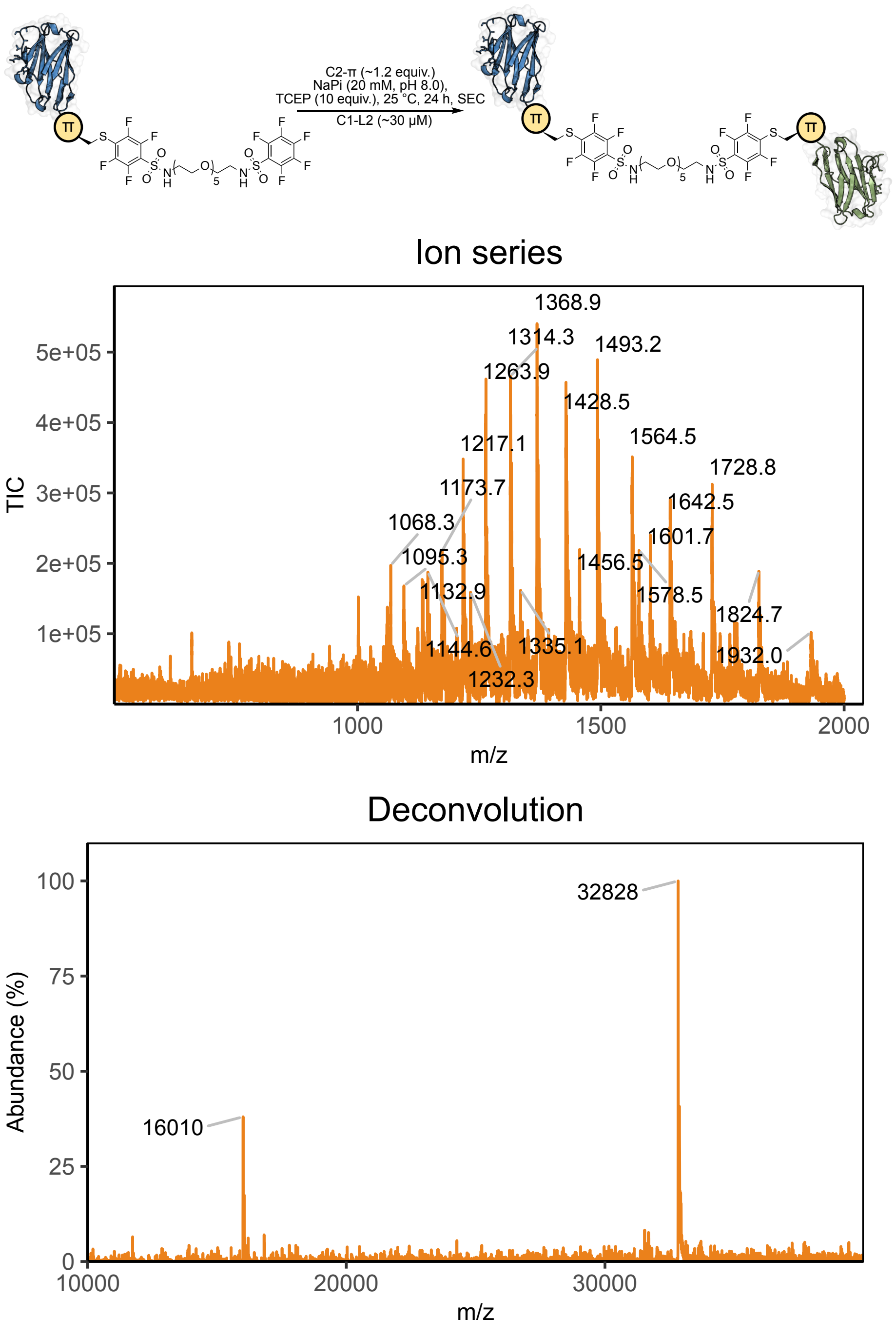

Figure S27: LC–MS spectra of C1-L2 incubated with C2-π (1 equivalents) at 25 °C in NaPi (20 mM pH 8.0 for 24 h; ion series and deconvoluted spectra (Calculated mass: 32825 kDa).

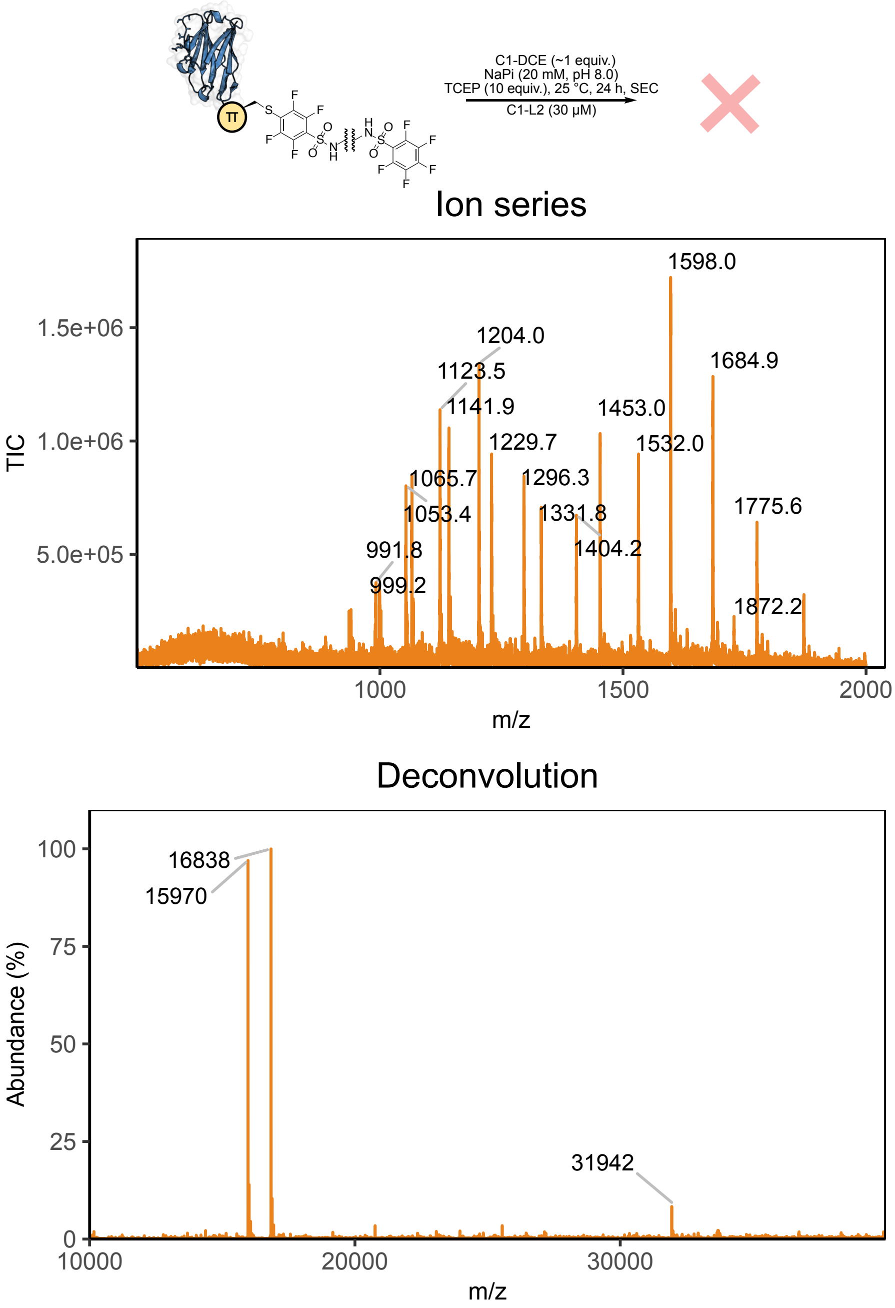

Figure S28: LC–MS spectrum of C1-π-L2 and C1-DCE control reaction to demonstrate necessity of a π-clamp cysteine residue for dimerisation; ion series and deconvoluted spectra (Calculated mass: 32786 kDa).

7.7.3 LC–MS analysis after SEC purification

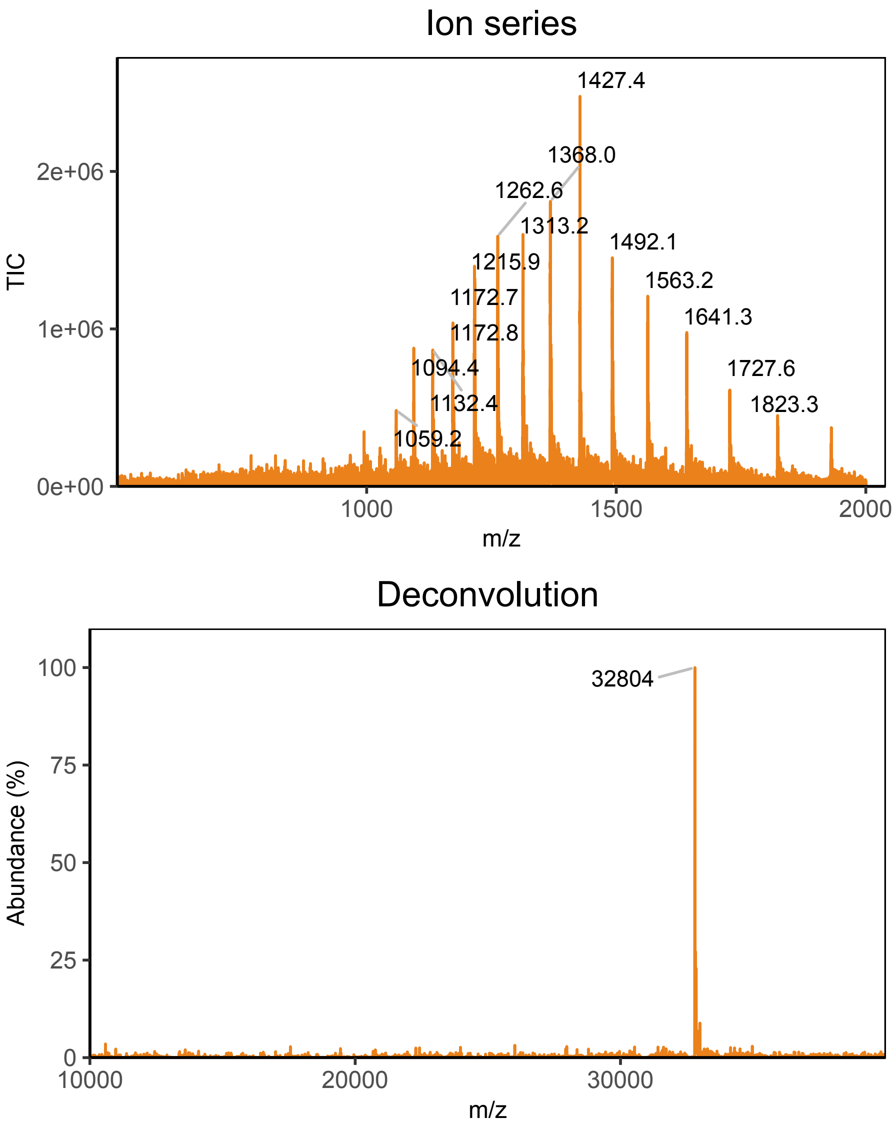

Figure S29: LC–MS spectrum of C1C1-L1 after SEC purification; ion series and deconvoluted spectra (Calculated mass: 32801 kDa).

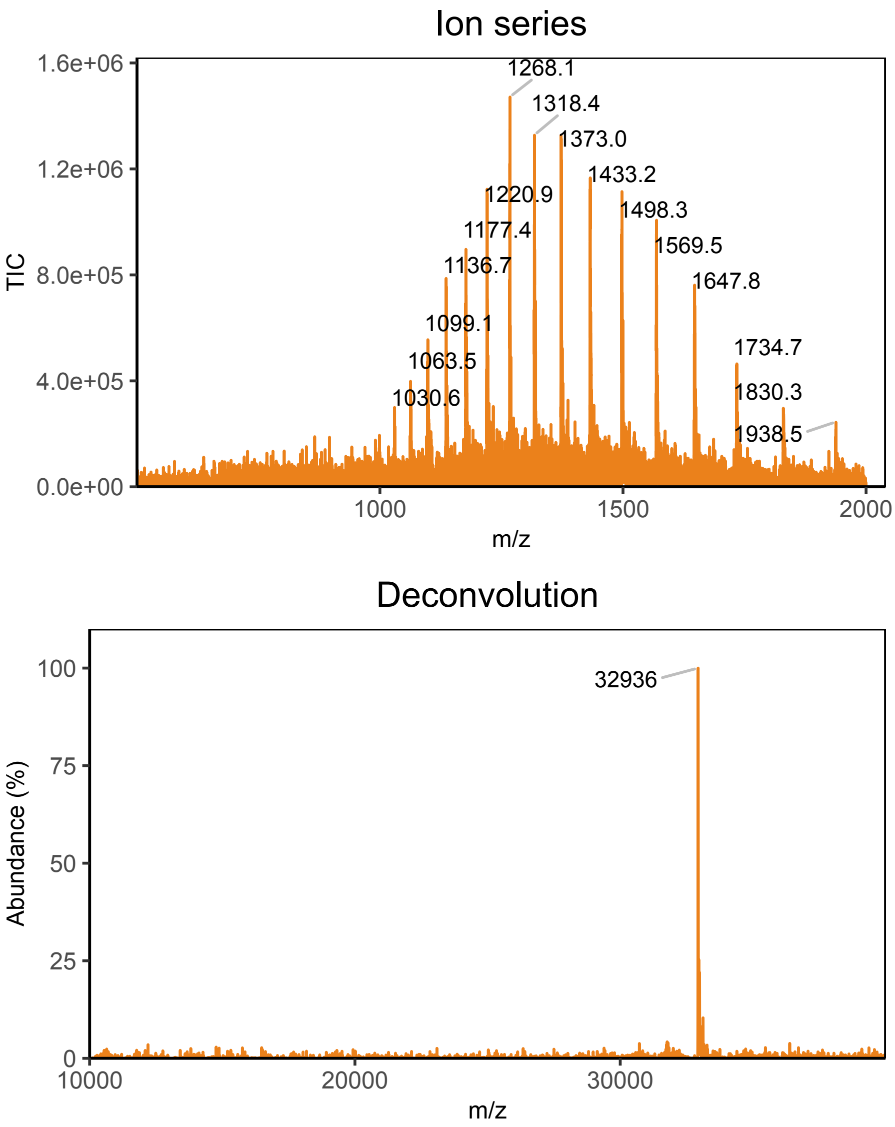

Figure S30: LC–MS spectrum of C1C1-L2 after SEC purification; ion series and deconvoluted spectra (Calculated mass: 32934 kDa).

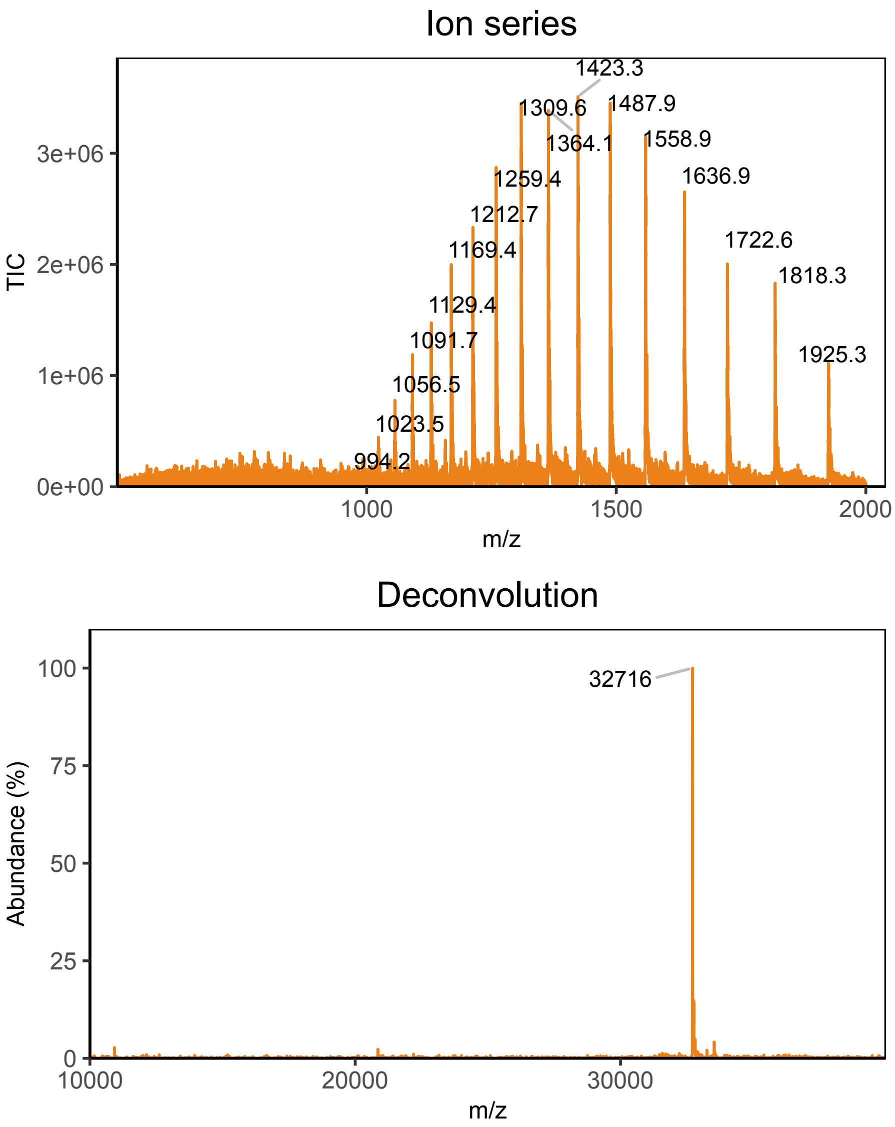

Figure S31: LC–MS spectrum of C2C2-L2 after SEC purification; ion series and deconvoluted spectra (Calculated mass: 32716 kDa).

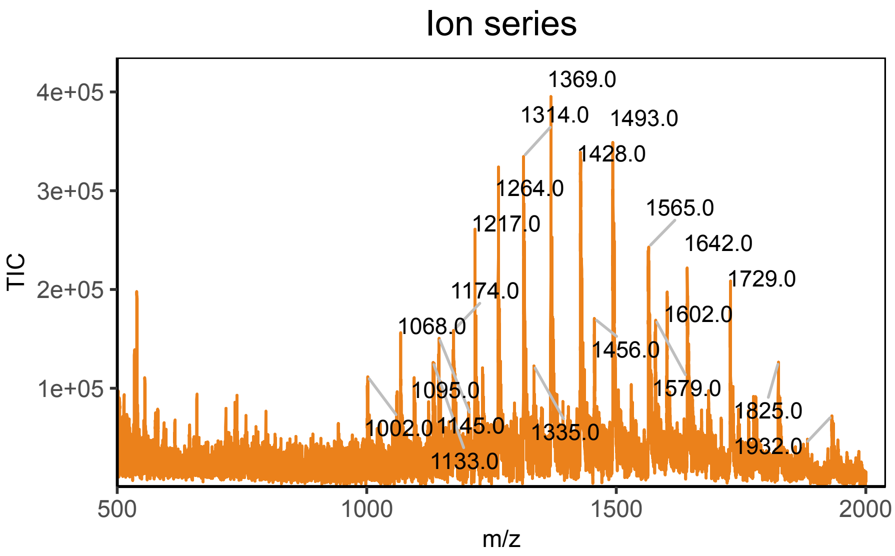

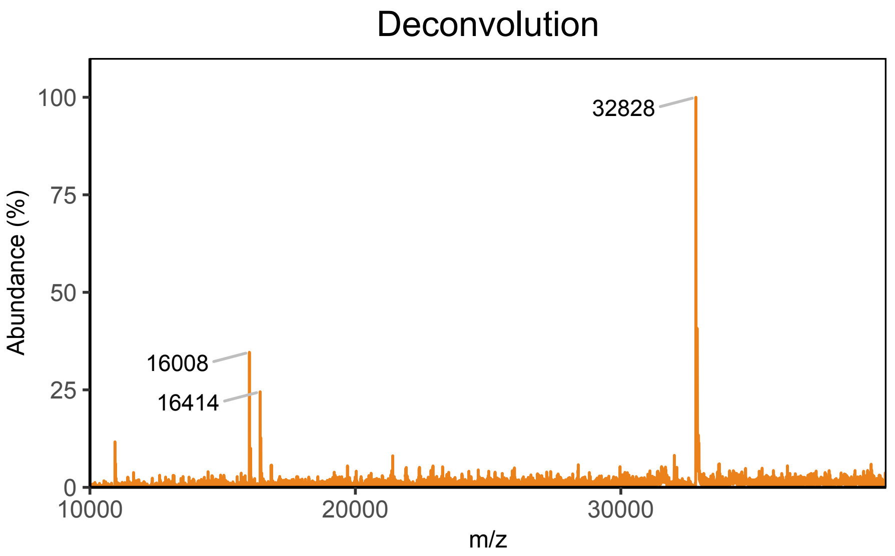

Figure S32: LC–MS spectrum of C1C2-L2 after SEC purification; ion series and deconvoluted spectra (Calculated mass: 32825 kDa).

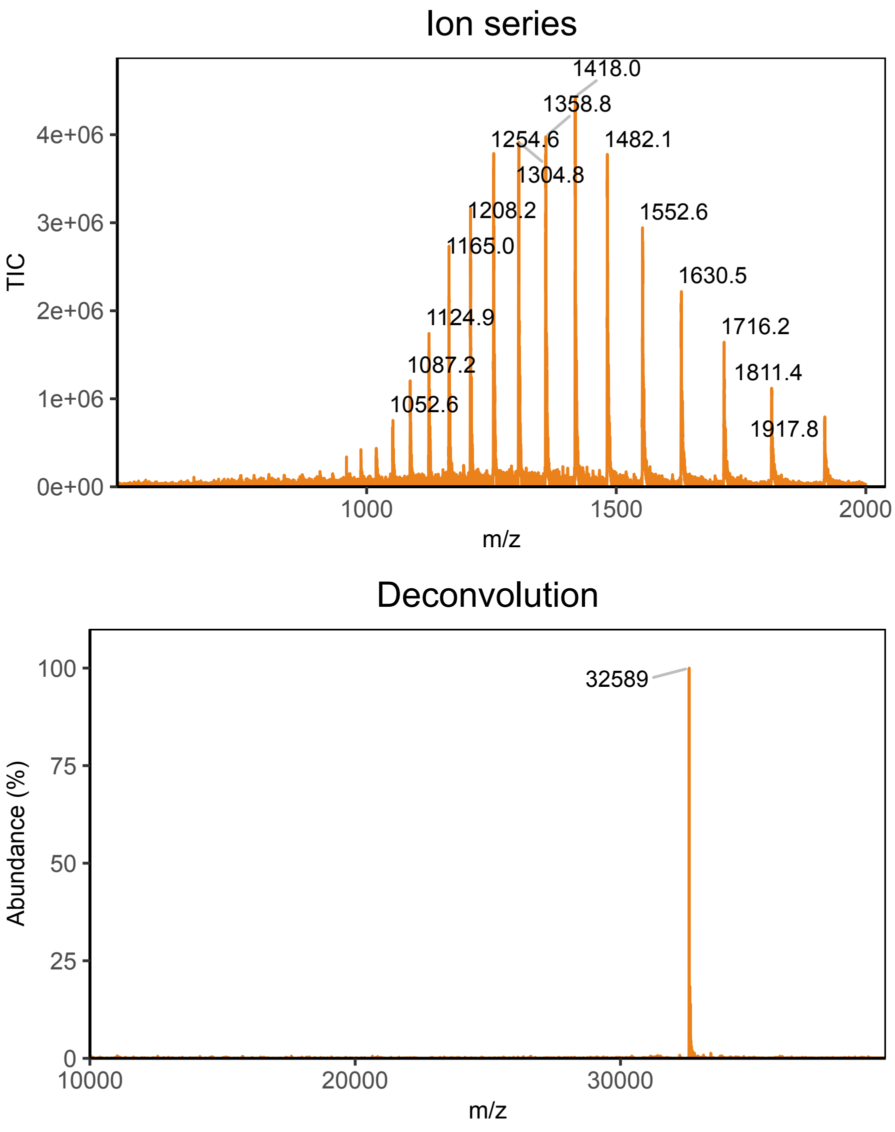

Figure S33: LC–MS spectrum of C1C1 maleimide dimer after SEC (Calculated mass: 32586 kDa).

7.8 Heterodimerisation of double cysteine containing variant

To DesAb C1-π (29.3 µM, 600 µL) in NaPi (20 mM pH 8.0), a DMF solution of Linker 2 (5.83 µL 20 equivalents, ~1% DMF, v/v) (50 mM, stock in DMF) was added along with TCEP (8.80 µL, 20 equivalents, 20 mM stock in 20 mM NaPi pH 8.0) and the reaction was left to incubate at 25 °C. Reaction progress was monitored by LC–MS analysis until completion, around 4 h (Figure S21. Purification was carried out by SEC using a HiLoad 10/300 Superdex 75 pg column (Cytiva, Little Chalfont, UK) and NaPi (pH 8.0) as the elution buffer to afford pure modified monomer (5.42 µM, 2.1 mL, 64%). The linker presenting C1-L2 monomer was concentrated to 64 µM and 116 µL, C1-π-DCE (66 µM, 112 µL, ~1 equivalents) (total protein concentration ~65 µM) was added along with TCEP (8 µL, ~20 equivalents, 20 mM stock in 20 mM NaPi pH 8.0) (20 mM stock in 20 mM, NaPi pH 8.0) and the reaction was incubated at 25 °C for 24 h. To the dimer, Alexa-647 C2 maleimide (Thermofisher, catalogue number: A20347) (16.35 µL, 10 mM in DMSO, 20 equivalents with respect to C1-π-DCE monomer) was added and the mixture incubated at 25 °C for 3 h. Purification and buffer exchange into TEV cleavage buffer (50 mM Tris-HCl pH 7.5, 0.5 mM EDTA, 0.4mM DTT) was carried out by SEC using a HiLoad 10/300 Superdex 75 pg column (Cytiva, Little Chalfont, UK). Dimer containing fractions were collected (1.40 µM, 750 µL, 33%) (overall yield, 21%) and purity was confirmed by LC–MS analysis (Figure S34). To this, 6 µL TEV protease (NEB) was added and incubated at 30 °C for 3 h. The digestion mixture was passed over Ni-NTA resin twice and the flow through was collected and analysed by LC–MS (Figure S35), and SDS–PAGE visualised by coomassie stain and an Alexa-647 filter (Figure 4).

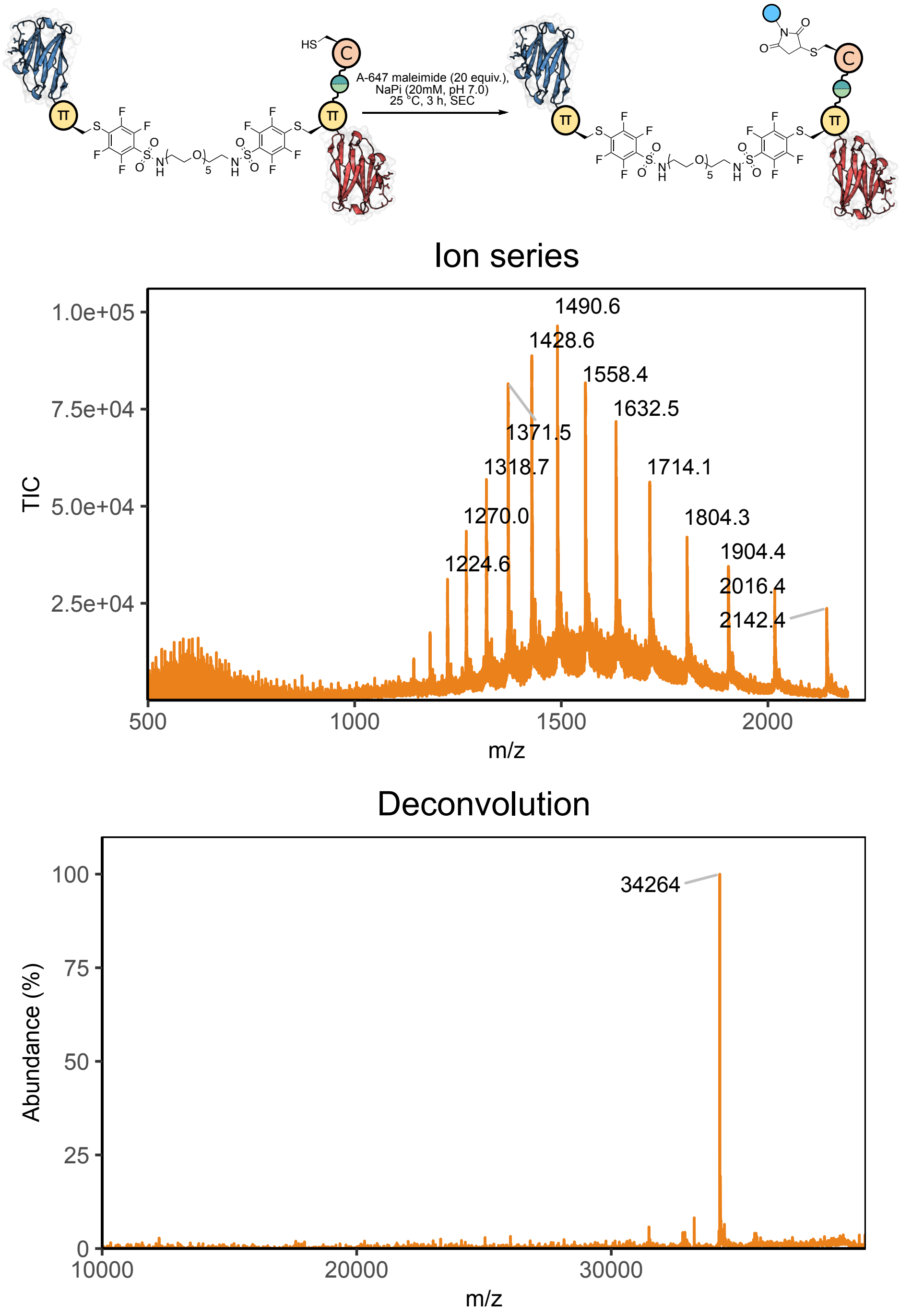

Figure S34: LC–MS spectrum of C1-π-C1-π-DCE L2 dimer labelled with Alexa-647 after SEC purification; ion series and deconvoluted spectra (Calculated mass: 34260 kDa).

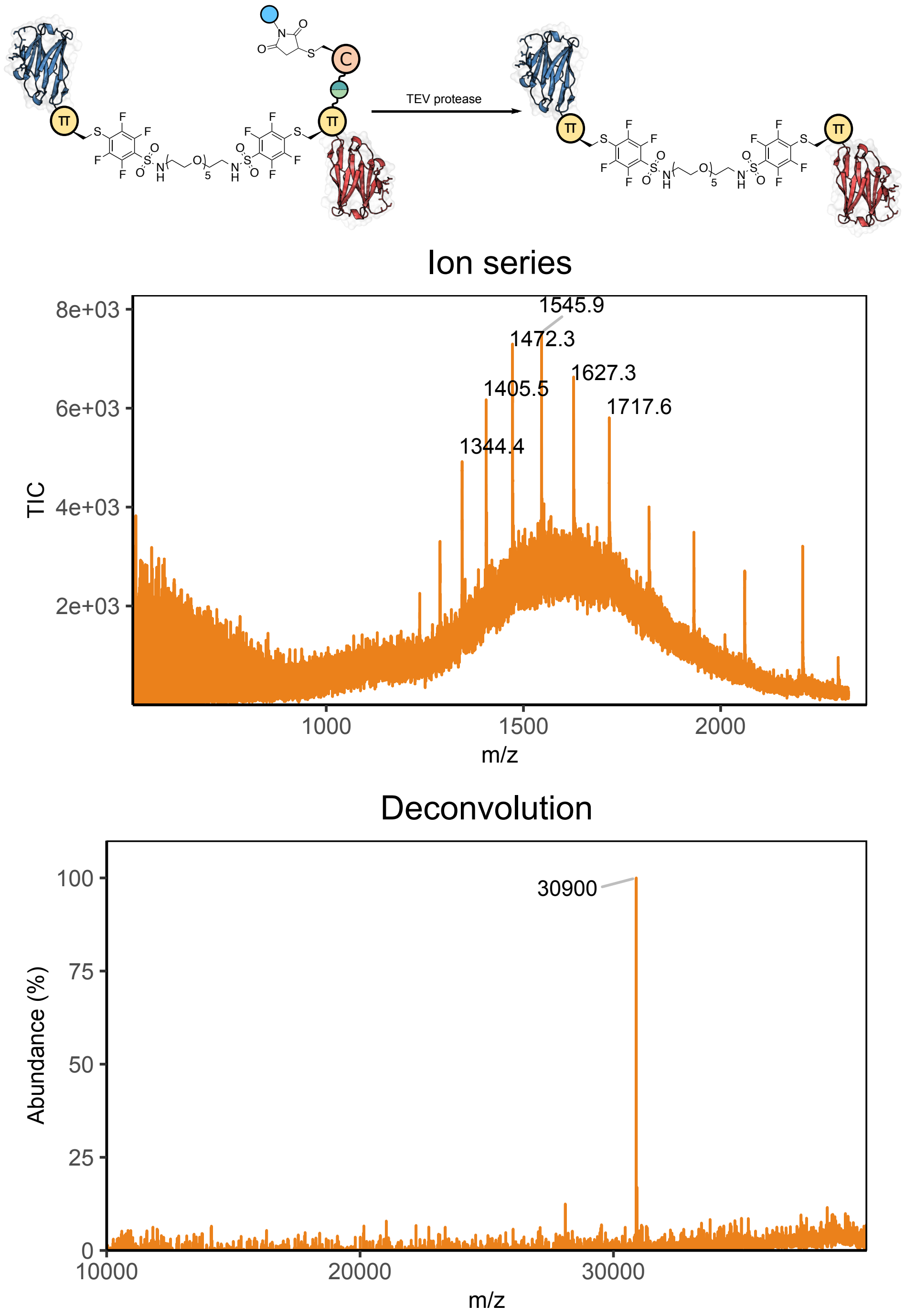

Figure S35: LC–MS spectrum of C1-π-C1-π-DCE L2 dimer labelled with Alexa-647 after SEC purification and TEV-cleavage to remove His-tags and Alexa-647; ion series and deconvoluted spectra (Calculated mass: 30899 kDa).

7.9 Glutathione stability assay

Samples of C1C1-L1 homodimer and C1C1 maleimide linker (95 µL, 2.5 µM, PBS pH 7.4) were prepared. 5 µL of a 40 mM glutathione (GSH) solution (6.14 mg GSH in 500 µL PBS, adjusted to pH 7.4) was added at room temperature and mixed thoroughly by pipetting up and down, resulting in a final GSH concentration of 2 mM. The resulting mixture was incubated at 37 °C. Time points were taken at 0, 24, 48 and 72 h and immediately flash frozen in liquid N_2. Samples were analysed by SDS–PAGE to observe thiol exchange of the linker over time and concomitant regeneration of monomeric species (Figure S36).

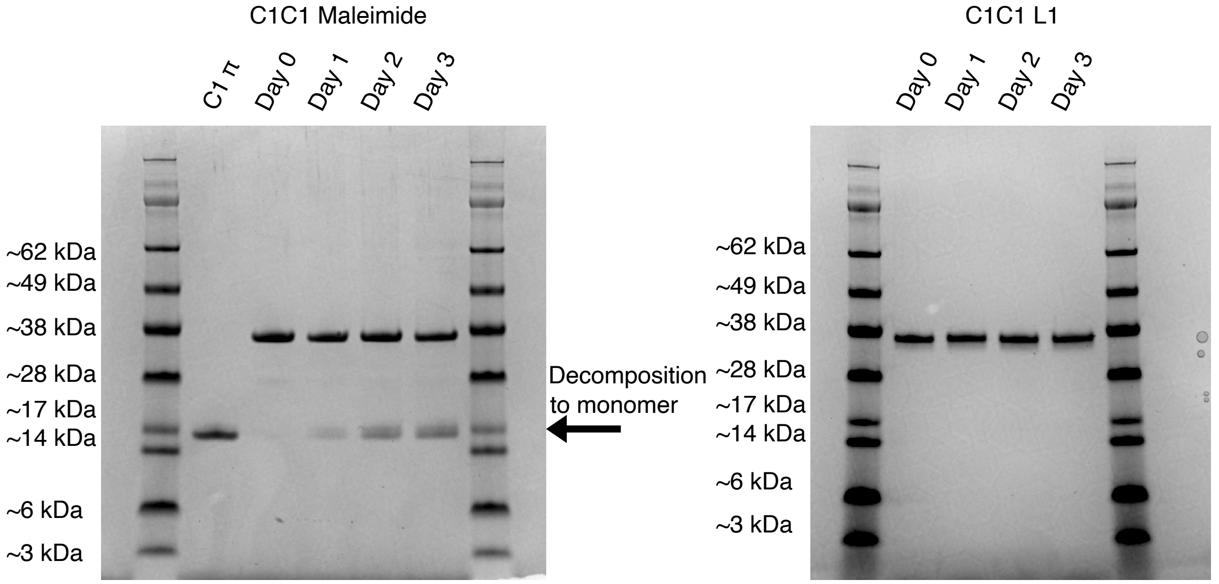

Figure S36: Stability analysis of C1C1-L1 vs C1C1-M by SDS–PAGE under reducing conditions incubated for 3 days in the presence of reduced GSH (2 mM) in PBS (pH 7.4) at 37 °C.

8 Linker extinction coefficient determination

To determine the effect of linker extinction coefficient on the desAbs at 280 nm a bicinchoninic acid assay (BCA) assay was carried out to measure total protein content of linker conjugated desAb constructs using a Pierce^{TM} BCA Protein Assay Kit (catalogue number: 23225). The unmodified desAb, C1-π, was used to generate a standard titration curve by plotting known C1-π concentrations (as determined by absorbance at 280 nm measured using a NanoDrop^{TM} 2000/2000c spectrophotometer and the theoretical ϵ = 31525 $M^{-1}cm^{-1}$, as calculated using ExPASy SIB Bioinformatics Resource Portal) against OD 562 nm.

Serial dilutions of modified monomeric C1-π-L1, and homodimeric C1C1-L1 were carried out in triplicate and the total protein concentration of the samples determined by interpolation of OD 562 nm values, following manufacturer's protocol. Absorbance of the samples at 280 nm was measured and the mean extinction coefficient for monomer C1-π-L1 was calculated to be 41385 $M^{-1}cm^{-1}$ and for dimer C1C1-L1 73356 $M^{-1}cm^{-1}$ using the Beer-Lambert Law. The additive effect of the small molecule linker upon the theoretical extinction coefficients were 9860 $M^{-1}cm^{-1}$ and 10306 $M^{-1}cm^{-1}$ for monomer and dimer, respectively (theoretical monomer ϵ = 31525 $M^{-1}cm^{-1}$ and theoretical dimer ϵ = 63050 $M^{-1}cm^{-1}$, as calculated using ExPASy SIB Bioinformatics Resource Portal). Considering the accuracy of the assay and instrumentation used, the conjugated small molecule linker was taken to have an extinction coefficient of 10000 $M^{-1}cm^{-1}$.

It was assumed that the PFP-sulfonamide groups of Linker 1 were responsible for absorbance at 280 nm of the linker molecules and that the additional PEG subunits of Linker 2 would not affect absorbance at 280 nm. Thus, Linker 1 was used as a representative model and 10000 $M^{-1}cm^{-1}$ was taken as the extinction coefficient for both conjugated PFP-sulfonamide Linkers 1 and 2.

9 Biophysical characterisation

9.1 Circular dichroism

Far ultraviolet (UV) circular dichroism (CD) spectroscopy of all constructs, measurements were recorded using a JASCO J-810 spectrophotometer equipped with thermally controlled Peltier holder using a quartz cuvette with a 0.1 cm path length. Protein samples were diluted in PBS to approximately 5 µM and 2.5 µM protein for monomers and dimers, respectively. CD spectra were obtained over a wavelength range of 200 to 250 nm at 25 °C with a bandwidth of 1 nm, a data pitch of 0.5 nm, a scanning speed of 50 nm/min and a response time of 4 s. All measurements were obtained as θ (mdeg) and the buffer spectrum was systematically subtracted. All data was subsequently normalised to mean residue ellipticity (MRE) (Eq. 1) in (deg·cm^{-2}·dmol^{-1}) to generate CD plots (Figure S37). Spectra were generated by averaging the data acquired from three technical replicates of 10 scans for each sample.

$$MRE = \frac{\theta(mdeg)}{10 \cdot C(M) \cdot l(cm) \cdot \#_{residues}} \tag{1}$$

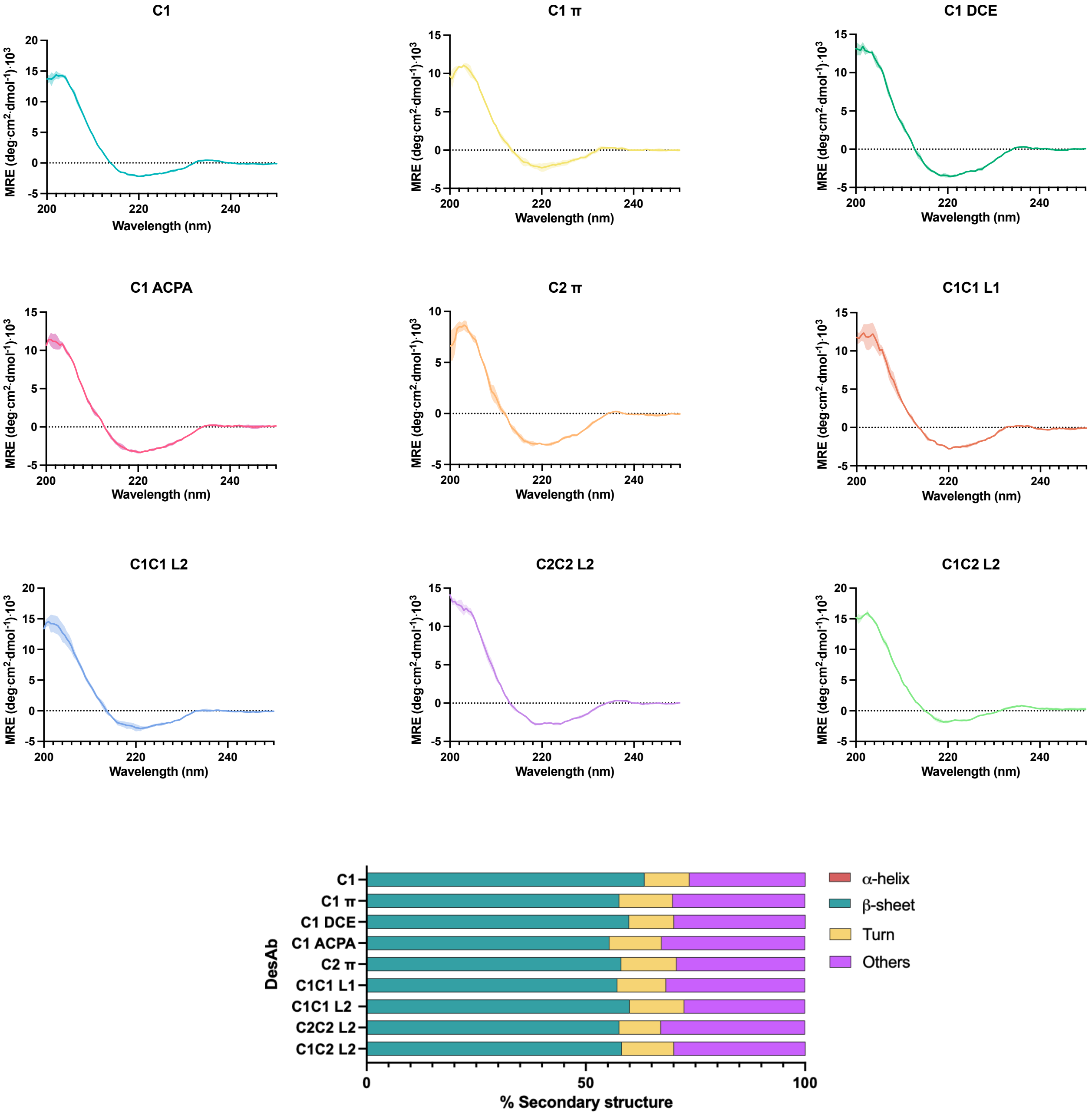

Figure S37: CD spectra of C1 and C2 desAb monomer and dimer constructs with expected minima at ∼218 nm characteristic of β-sheet secondary structure. Stacked barchart shows the percentage secondary structure content of each concstruct as determined using BeSt-Sel.[S3,S4]

9.2 Tycho thermal denaturation assays

Expressed and subsequently modified proteins' structural integrity were assessed using Tycho NT.6 (NanoTemper Technologies). All samples were measured in PBS buffer (pH 7.4) at approximately 5 µM and 2.5 µM protein for monomers and dimers, respectively. Intrinsic fluorescence of tryptophan and tyrosine residues was recorded at 330 nm and 350 nm on a 30 °C/min temperature ramp from 35–95 °C. The ratio of fluorescence intensity (350/330 nm) and the inflection temperature T_i were calculated by software on the Tycho NT.6 instrument (Figure S38 and S39).

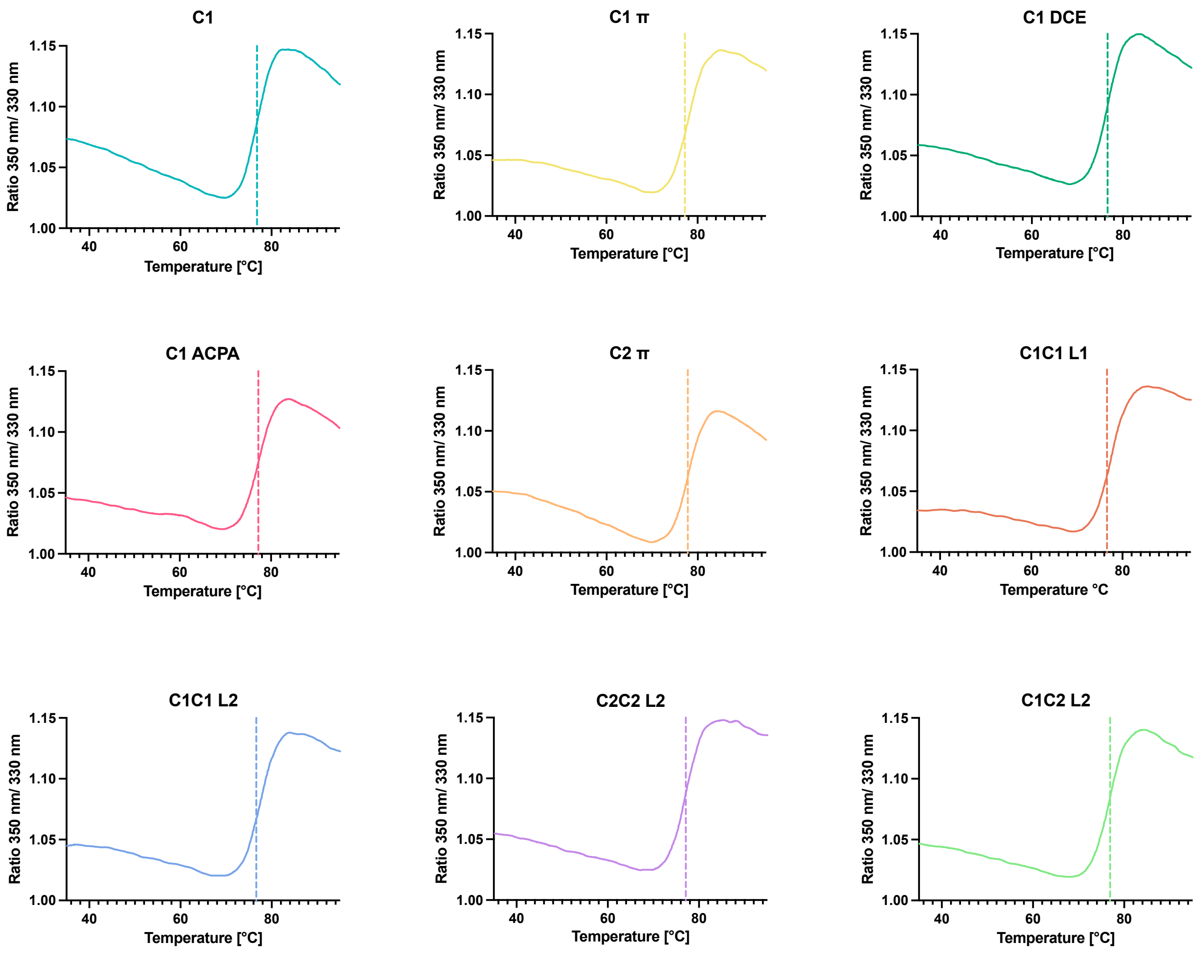

Figure S38: Tycho thermal denaturation profiles of C1 and C2 monomeric and dimeric constructs, with T_i indicated by a vertical line.

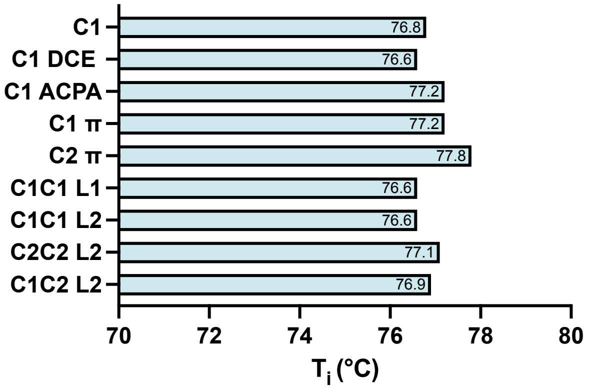

Figure S39: T_i values for C1 and C2 monomeric and dimeric constructs from Tycho thermal denaturation.

10 Antibody binding assays

10.1 Alexa-647 NHS ester labelling of the SARS-CoV-2 Spike protein for MST and R_h measurements

SARS-CoV-2 Spike protein (The Native Antigen Company: REC31871-100) was labelled with Alexa-647 NHS ester as follows. To the Spike protein (72 µL, 2.82 µM, PBS pH 7.4) sodium bicarbonate (8 µL, 1 M) was added to achieve a final total volume of 80 µL with 100 mM sodium bicarbonate. To this, a DMSO solution of Alexa-647 NHS ester (2.64 µL, 3.85 mM, 50 equivalents) was added to the Spike protein and incubated at room temperature in the dark for 2 h. Excess dye was removed by desalting thrice with 7 kDa MWCO Zeba^{TM} Spin Desalting Columns equilibrated with aqueous PBS buffer (pH 7.4). The resulting protein concentration and labelling efficiency were subsequently measured using a NanoDrop^{TM} 2000/2000c spectrophotometer and found to be an average of 13 Alexa-647 molecules per spike protein. As labelling was carried out using an excess of Alexa-647-NHS ester, this resulted in random labelling of exposed lysine residues. Considering the trimeric spike has >150 lysine residues, it was highly unlikely that the labelling would interfere with the accessibility of the target epitopes, which themselves do not contain any lysine residues.[S5]

10.2 Microscale thermophoresis (MST) binding affinity measurements

Starting from 2 µM of desAb dimer constructs, 14.4 µM of desAb monomer constructs C1 and C2, and 4 µM of desAb P2, 16 samples of 1:1 serial dilutions were incubated with 8 nm Alexa-647 labelled Spike protein for 1 h at room temperature. Samples were prepared in PBS pH 7.4 including 0.05% Tween-20. Subsequently, samples were run in triplicate in a Monolith NT.115 System (NanoTemper technologies) using 15% LED excitation power and 80% MST power, at 25 °C. Data was analysed and fitted using the Monolith System software assuming a 1:1 binding interaction. It should be noted that MST curves were fitted using a standard 1:1 binding model making K_D values apparent, due to the trimeric nature of the spike protein.

Table S6: K_Ds of C1, C2 and P2 constructs binding to the spike protein with corresponding errors (±S.D.) as measured by MST.

desAb	K_D (nM)
C1-π	146 ± 61
C2-π	581 ± 240
C1C1-L1	8 ± 4
C1C1-L2	15 ± 10
C2C2-L2	10 ± 5
C1C2-L2	10 ± 3
C1C1-M	11 ± 6
P2	-

10.3 Hydrodynamic radius measurements using Fluidity One W

The hydrodynamic radius (R_h) of Alexa-647 labelled Spike protein was measured alone and in the presence of C1-π and C1C1-L2 desAbs using the Fluidity one W instrument (Fluidity Analytics) based on microfluidic diffusional sizing.[S6] C1-π and C1C1-L2 were used as general representative models for monomeric and dimeric constructs, respectively. Ligand concentrations from the upper, lower and inflection regions of the MST binding curves were selected for both C1-π (2 µM, 100 nM, 0.2 nM) and C1C1-L2 (1 µM, 50 nM, 0.1 nM) and incubated with 8 nM Alexa-647 labelled Spike protein for 1 h at room temperature. Samples were prepared in PBS pH 7.4 with 0.05% Tween-20. 6 µL of each sample was used for each measurement which was completed between 3-6 times and plotted as the mean with standard deviation (Figure 5).

11 ^{1}H, ^{13}C and ^{19}F NMR Spectra

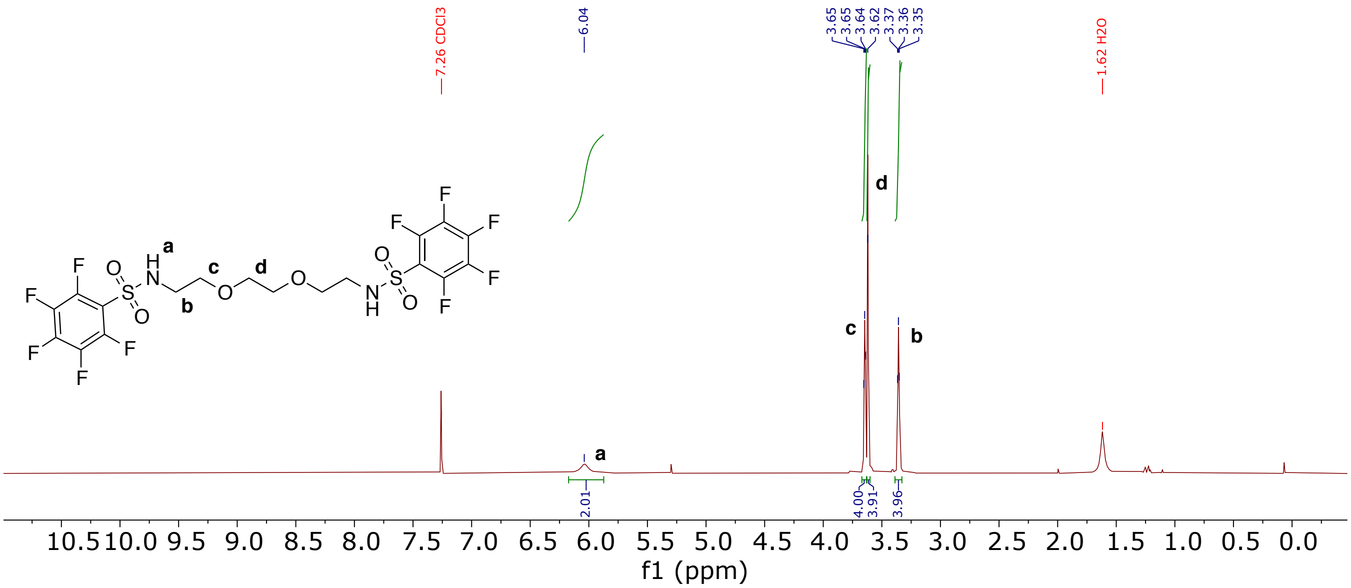

Figure S40: ^{1}H NMR spectrum of N,N′-((ethane-1,2-diylbis(oxy))-bis(ethane-2,1-diyl))bis(2,3,4,5,6-pentafluorobenzenesulfonamide) (Linker 1)

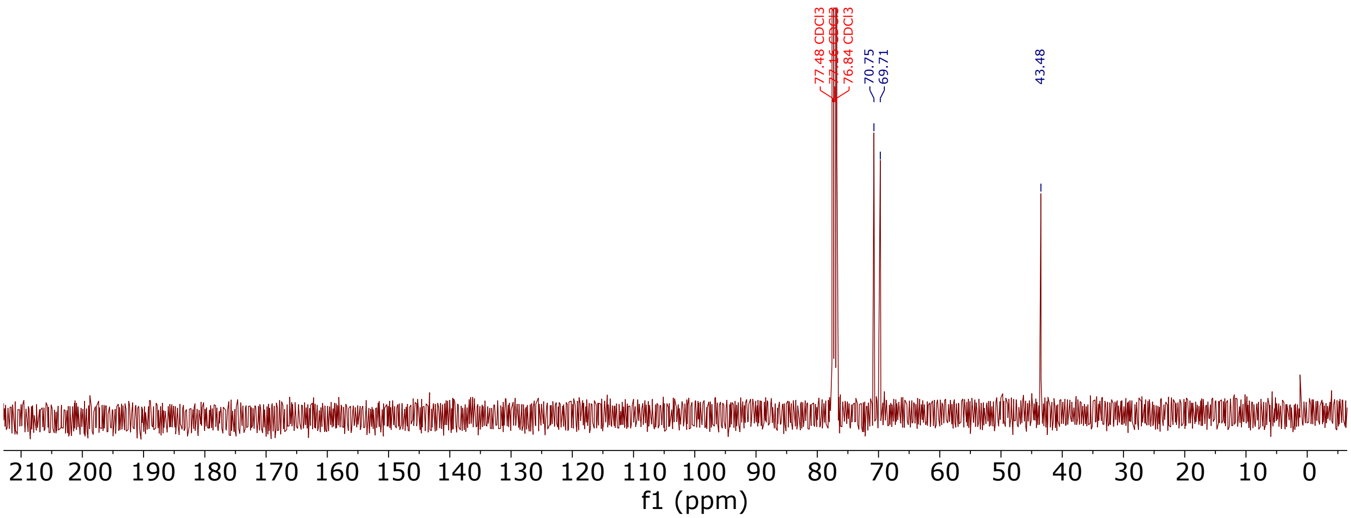

Figure S41: ^{13}C NMR spectrum of N,N′-((ethane-1,2-diylbis(oxy))-bis(ethane-2,1-diyl))bis(2,3,4,5,6-pentafluorobenzenesulfonamide) (Linker 1)

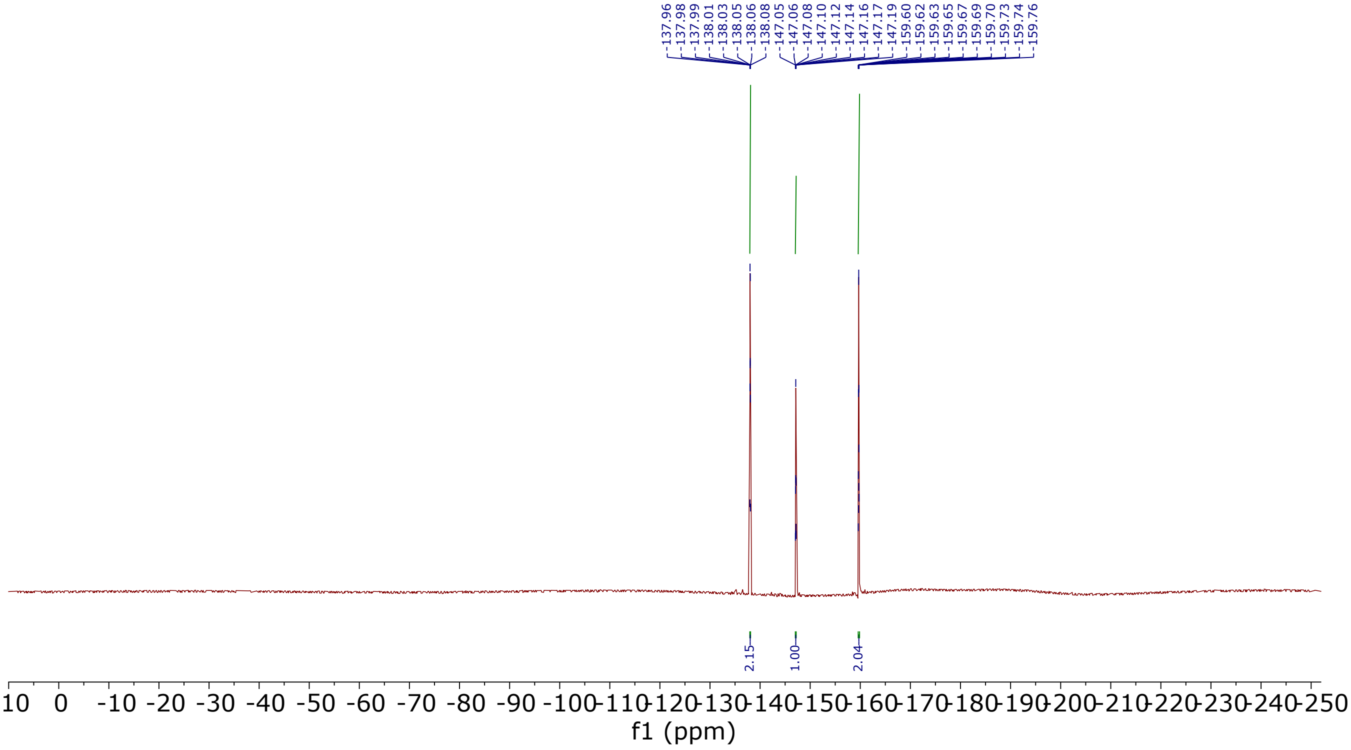

Figure S42: ^{19}F NMR spectrum of N,N′-((ethane-1,2-diylbis(oxy))-bis(ethane-2,1-diyl))bis(2,3,4,5,6-pentafluorobenzenesulfonamide) (Linker 1)

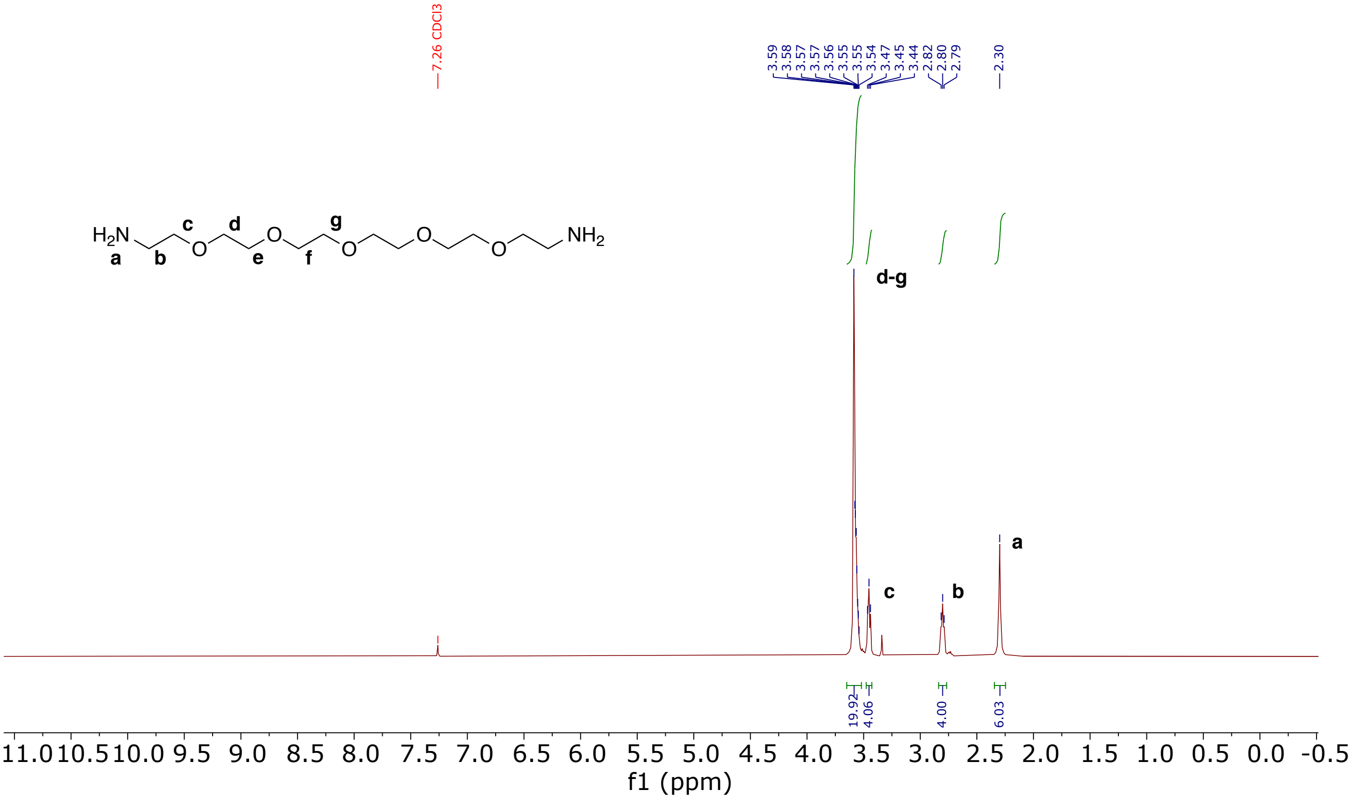

Figure S43: ^{1}H NMR of 3,6,9,12,15-pentaoxaheptadecane-1,17-diamine

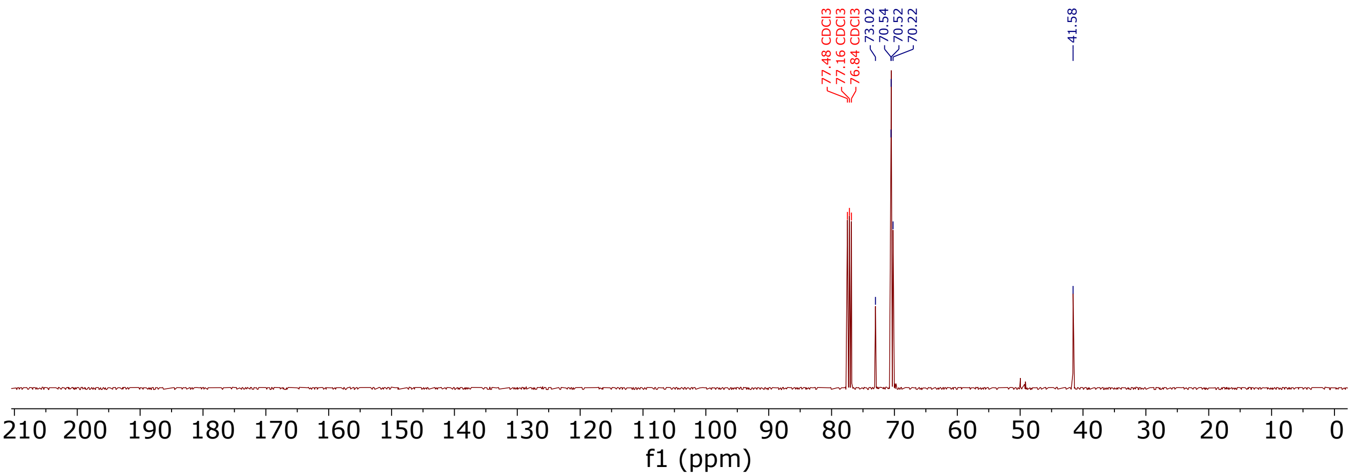

Figure S44: ^{13}C NMR of 3,6,9,12,15-pentaoxaheptadecane-1,17-diamine

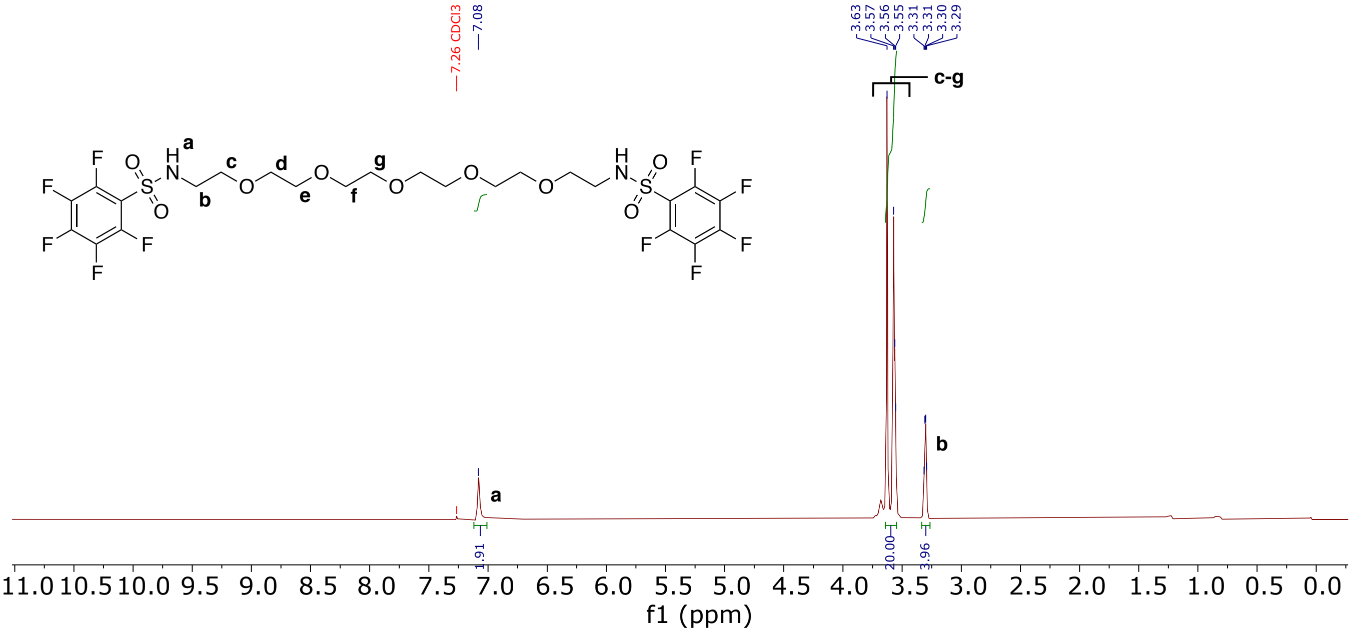

Figure S45: ^{1}H NMR of N,N′-(3,6,9,12,15-pentaoxaheptadecane-1,17-diyl)bis(2,3,4,5,6-pentafluorobenzenesulfonamide) (Linker 2)

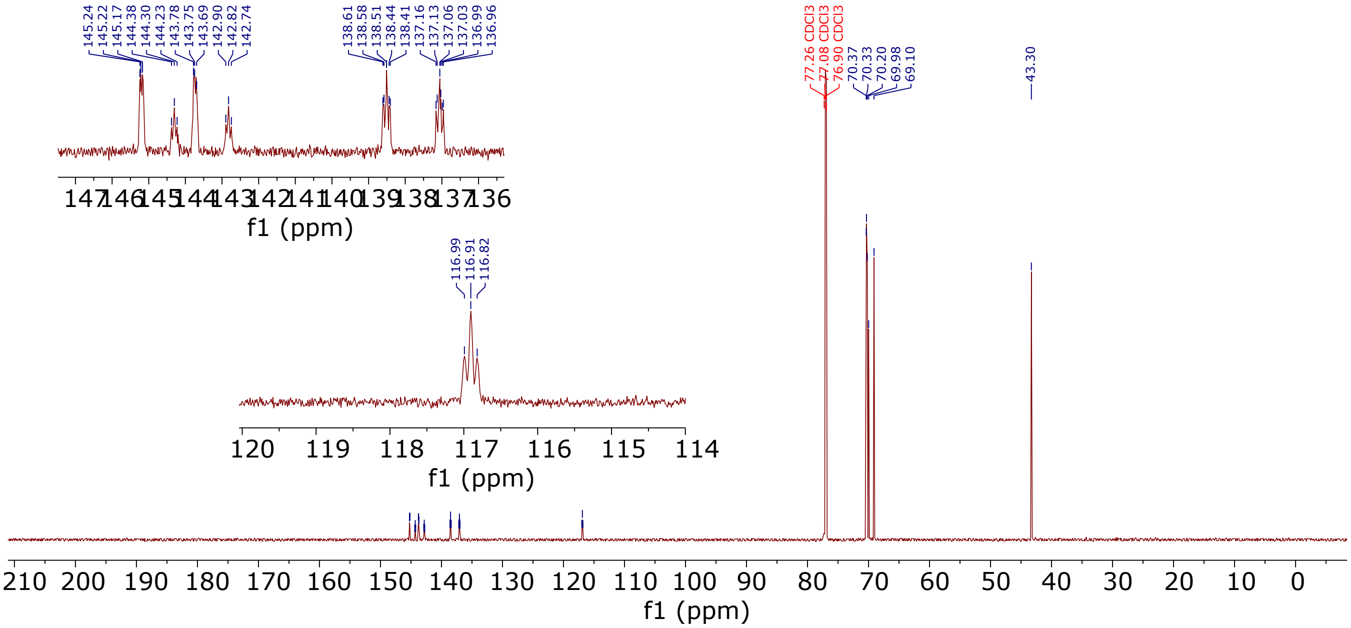

Figure S46: ^{13}C NMR N,N′-(3,6,9,12,15-pentaoxaheptadecane-1,17-diyl)bis(2,3,4,5,6-pentafluorobenzenesulfonamide) (Linker 2)

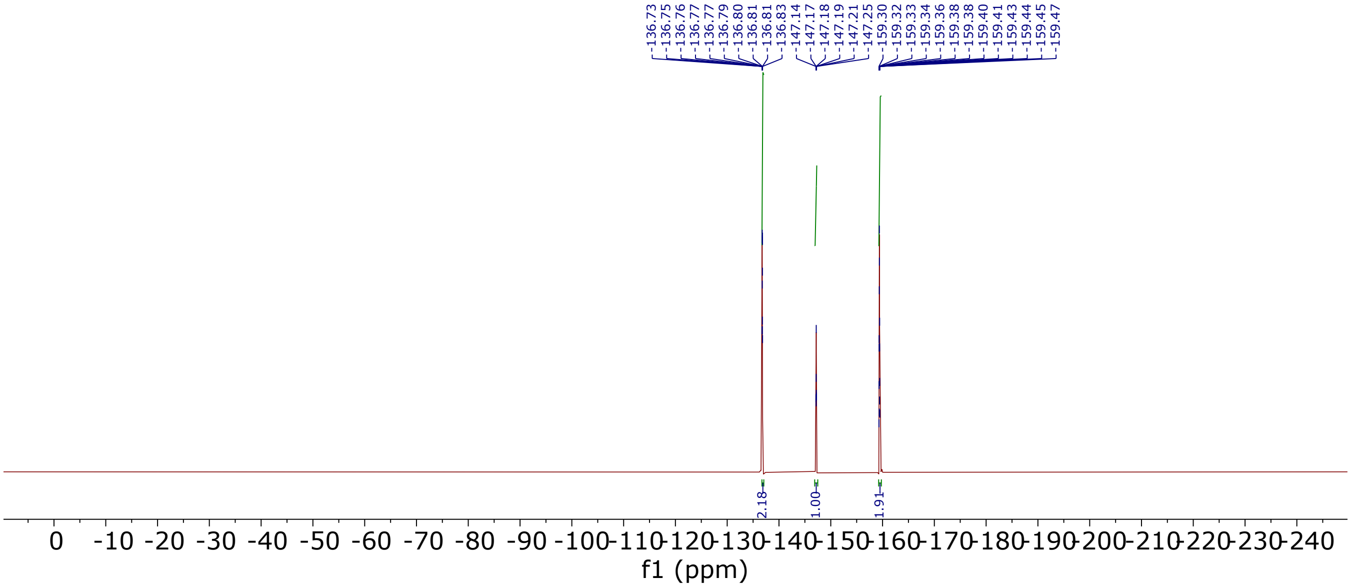

Figure S47: ^{19}F NMR N,N′-(3,6,9,12,15-pentaoxaheptadecane-1,17-diyl)bis(2,3,4,5,6-pentafluorobenzenesulfonamide) (Linker 2)

References

(S1) Rangel, M. A.; Bedwell, A.; Costanzi, E.; Taylor, R.; Russo, R.; Bernardes, G. J.; Ricagno, S.; Frydman, J.; Vendruscolo, M.; Sormanni, P. Fragment-based computational design of antibodies targeting structured epitopes. 2022-04-21. *bioRxiv.* `https://doi.org/10.1101/2021.03.02.433360`. (accessed 2022-07-07).

(S2) Bond, S. R.; Naus, C. C. RF-Cloning.org: an online tool for the design of restriction-free cloning projects. *Nucleic Acids Res.* **2012**, *40*, W209–W213.

(S3) Micsonai, A.; Wien, F.; Kernya, L.; Lee, Y.-H.; Goto, Y.; Réfrégiers, M.; Kardos, J. Accurate secondary structure prediction and fold recognition for circular dichroism spectroscopy. *Proc. Natl. Acad. Sci. U.S.A.* **2015**, *112*, E3095–E3103.

(S4) Micsonai, A.; Wien, F.; Bulyáki, E.; Kun, J.; Moussong, E.; Lee, Y.-H.; Goto, Y.; Réfrégiers, M.; Kardos, J. BeStSel: a web server for accurate protein secondary structure prediction and fold recognition from the circular dichroism spectra. *Nucleic Acids Res.* **2018**, *46*, W315–W322.

(S5) Wrapp, D.; Wang, N.; Corbett, K. S.; Goldsmith, J. A.; Hsieh, C.-L.; Abiona, O.; Graham, B. S.; McLellan, J. S. Cryo-EM structure of the 2019-nCoV spike in the prefusion conformation. *Science* **2020**, *367*, 1260–1263.

(S6) Yates, E. V.; Müller, T.; Rajah, L.; De Genst, E. J.; Arosio, P.; Linse, S.; Vendruscolo, M.; Dobson, C. M.; Knowles, T. P. J. Latent analysis of unmodified biomolecules and their complexes in solution with attomole detection sensitivity. *Nat. Chem.* **2015**, *7*, 802–809.
